# Spatial cellular order underlies locally-confined mechanisms of immune resistance in oropharyngeal cancer

**DOI:** 10.1038/s41467-026-74318-z

**Published:** 2026-06-13

**Authors:** Cem Sievers, Yvette Robbins, Marco Craveiro, Jay Friedman, Angel Huynh, Xinping Yang, Michael Kelly, James W. Hodge, Dilara Akbulut, Martha Quezado, Wojciech Mydlarz, Nyall R. London, Nancy Judd, John Deeken, Gopal Bajaj, Tian-Gen Chang, Clint T. Allen, Charalampos S. Floudas

**Affiliations:** 1 Head and Neck Section, Surgical Oncology Program, Center for Cancer Research, National Cancer Institute, National Institutes of Health, Bethesda, MD USA; 2 Bioinformatics Section, Surgical Oncology Program, Center for Cancer Research, National Cancer Institute, National Institutes of Health, Bethesda, MD USA; 3 Single Cell Analysis Facility, Center for Cancer Research, National Cancer Institute, National Institutes of Health, Bethesda, MD USA; 4 Center for Immuno-Oncology, Center for Cancer Research, National Cancer Institute, National Institutes of Health, Bethesda, MD USA; 5 Laboratory of Pathology, Center for Cancer Research, National Cancer Institute, National Institutes of Health, Bethesda, MD USA; 6 Department of Otolaryngology-Head and Neck Surgery, Johns Hopkins School of Medicine, Baltimore, MD USA; 7 Sinonasal and Skull Base Tumor Section, Surgical Oncology Program, Center for Cancer Research, National Cancer Institute, National Institutes of Health, Bethesda, MD USA; 8 Kaiser Permanente of Northern Virginia, Alexandria, VA USA; 9 Inova Schar Cancer Institute, Inova Health System, Fairfax, VA USA; 10 Cancer Data Science Laboratory, Center for Cancer Research, National Cancer Institute (NCI), National Institutes of Health (NIH), Bethesda, MD USA

**Keywords:** Tumour immunology, Immune evasion, Tumour heterogeneity

## Abstract

Oropharyngeal squamous cell carcinomas (OPSCCs) frequently result from oncogenic human papilloma virus (HPV) infections (HPV-OPSCC). The mechanisms underlying effective immune escape, despite abundant viral antigens, are incompletely understood. Here, we performed single-cell spatial gene expression profiling of HPV-OPSCC to characterize cellular organization and mechanisms of immune resistance. We describe distinct tumor-parenchymal immune foci that differ in cytokine expression, spatial location, immune cell infiltration and cancer cell states. Furthermore, immune foci display profound differences related to co-inhibitory receptor signaling and immunosuppressive myeloid cells, suggesting that different tumor-parenchymal regions may be dominated by distinct, locally-confined mechanisms of immunosuppression. Additionally, senescent-like HPV-OPSCC cells lacking HPV transcripts (HPVoff) are evident across the tumor parenchyma and able to evade HPV-specific T cell-mediated immunity in vitro. HPVoff cells are enriched within hypoxic regions and near IFN-γ producing T cells suggesting that both hypoxia and IFN-γ signaling can promote the HPVoff phenotype. In conclusion, our findings highlight a complex cellular interplay underlying heterogeneous cancer cell states, spatial immune cell organization, and diverse mechanisms of immune escape.

## Abstract (web summary)

Mechanisms underlying effective immune escape in HPV-associated oropharyngeal cancers despite the presence of immune cells are incompletely understood. Here, authors use single-cell spatial analysis to show that tumors form distinct local immune niches, including hypoxic regions and chemokine foci, that shape immune cell composition and support cancer cell states able to evade HPV-specific T cell responses.

## Introduction

Malignant transformation following chronic HPV infection is the most common cause of oropharyngeal squamous cell carcinoma (HPV-OPSCC)^1^. Pathogenesis of HPV-associated malignancies involves HPV genes E6 and E7, which effectively inhibit the tumor suppressors p53 and Rb, respectively^2,3^. How HPV-OPSCC evades anti-tumor immunity, despite the presence of viral antigens, is less well understood. Single-cell RNA-sequencing (scRNA-seq) analyses have described extensive diversity related to cell types and cellular states within HPV-OPSCC^4–6^. In addition to immune diversity, HPV-OPSCC cells lacking detectable HPV transcripts (HPVoff), characterized by reduced cell-cycle activity and epithelial senescence, have been described^6^. The mechanisms underlying HPVoff cancer cell formation, their spatial interactions with immune cell types, and their significance in the context of HPV-OPSCC pathogenesis are still unclear.

Here, we performed single-cell spatial gene expression (SGE) profiling in combination with scRNA-seq to better characterize spatial cellular relationships within HPV-OPSCC. We describe classes of immune foci that exhibit differences in cytokine expression, spatial location, immune cell composition, and cancer cell states. Furthermore, immune foci differ in co-inhibitory receptor signaling and infiltration of immunosuppressive myeloid cells, suggesting that effective immune evasion may be mediated through different, locally confined mechanisms of immunosuppression. In addition, our analysis implicates both hypoxia and IFN-γ signaling in reduced HPV gene expression and the formation of HPVoff cells. In summary, our study reveals detailed insights into the spatial cellular order within HPV-OPSCC underlying the development of heterogeneous immune phenotypes, cancer cell states, and diverse modes of immune evasion.

## Results

### Combined single-cell SGE profiling and scRNA-seq in HPV-OPSCC

**Fig. 1:**
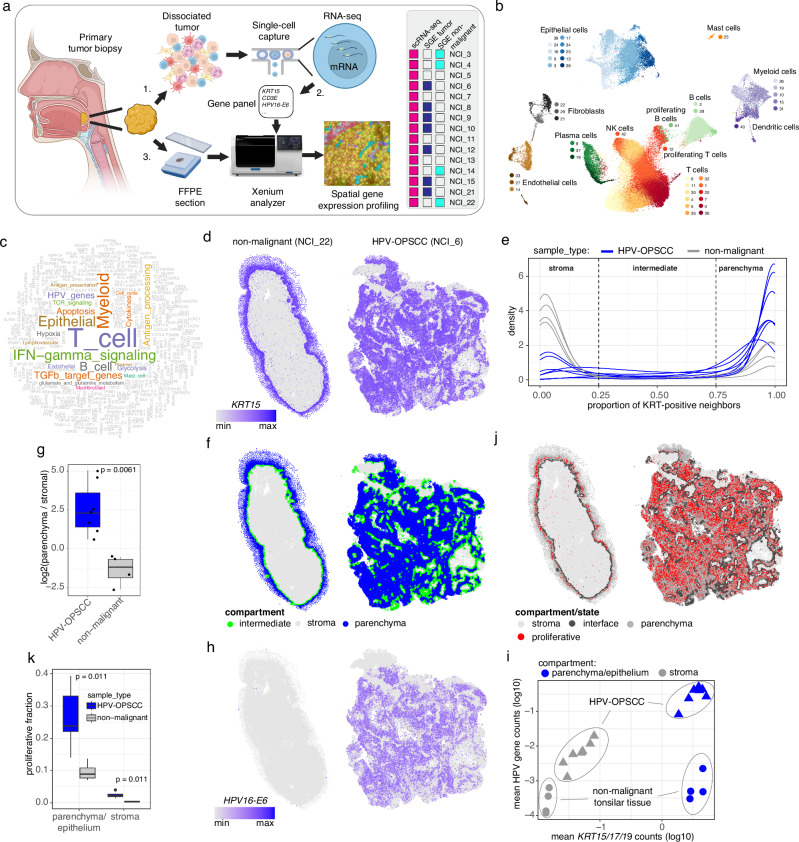
Classification of tumor parenchymal and stromal compartments. **a** Schematic illustrating the data-generating process. Colored squares (right) indicate data availability by patient identity. Created in BioRender (https://BioRender.com/asaomg7). **b** Scatter plot showing uniform manifold approximation and projection (UMAP) embedding of cells obtained from HPV-OPSCC scRNA-seq, colored by cluster identity and inferred cell type. **c** Word cloud showing genes selected for spatial gene expression profiling in gray and the associated functional terms in color. Font size scales with the number of genes associated with the respective term. **d**, **f**, **h**, **j** Scatter plots showing cells within non-malignant epithelial (left) and HPV-OPSCC tissues (right). Colors correspond to KRT15 expression (**d**), compartment classification (**f**), HPV16E6 expression (**h**), or to compartment classification, additionally indicating proliferative cells (**j**). Source data are provided as a Source Data file (**f**, **j**). **e** A line graph showing the probability density of keratin (KRT) – positivity representing the fraction of KRT-positive cells within the 20 nearest neighbors for all cells in a tissue sample. Cells were considered KRT-positive if at least one transcript of KRT15, KRT17, or KRT19 was detected. **g** Box plot showing the ratio of parenchymal to stromal cells for HPV-OPSCC (*n* = 7) and non-malignant epithelial (*n* = 4) samples. The box corresponds to the interquartile range (IQR), the horizontal line inside the box indicates the median, whiskers (vertical bars) extend to the smallest and largest data points within 1.5*IQR from the lower and upper quartiles, respectively, and data points beyond the boundaries of whiskers reflect outliers. *P*-value; two-sided Wilcoxon rank-sum test. Source data are provided as a Source Data file. **i** Scatter plot showing mean KRT15, KRT17, and KRT19 counts and mean HPV16 gene counts separately computed for stromal and parenchymal cells of each sample. Source data are provided as a Source Data file. **k** Box plot showing proportion of proliferative cells for each compartment within HPV-OPSCC (*n* = 7) and non-malignant epithelial (*n* = 4) samples. The box corresponds to the interquartile range (IQR), the horizontal line inside the box indicates the median, whiskers (vertical bars) extend to the smallest and largest data points within 1.5*IQR from the lower and upper quartiles, respectively, and data points beyond the boundaries of whiskers reflect outliers. *P*-value; two-sided Wilcoxon rank-sum test; **: *p* ≤ 0.01. Source data are provided as a Source Data file.

To study the spatial order of distinct cell types within the tumor microenvironment (TME), we obtained biopsies from 15 patients with newly diagnosed, treatment-naïve HPV-OPSCCs harboring wild-type *TP53* (Supplementary Table 1). We performed scRNA-seq followed by single-cell SGE profiling for a subset of patients (Fig. 1a). scRNA-seq was performed using whole biopsy digests to study all cell types present within the TME, resulting in transcriptome data from 105,136 cells (Supplementary Fig. 1a). HPV16 or HPV33 gene expression was detectable within scRNA-seq data from each sample (Supplementary Fig. 1b). All cells were grouped into 42 distinct clusters using graph-based clustering (Supplementary Fig. 1c). The resulting cell clusters were assigned cell types, based on expression of established lineage and cell markers (Fig. 1b and Supplementary Fig. 1c, d). We next used scRNA-seq data to derive a custom probe panel targeting 297 genes that reflect distinct cell types and physiological states, including HPV genes (Fig. 1c, Supplementary Dataset 1), for SGE profiling at single-cell resolution using the 10X Genomics Xenium platform^7^. The resulting probe set was used for single-cell SGE profiling of seven HPV16-positive HPV-OPSCC samples and, for comparison, four pathologically annotated regions of adjacent non-malignant mucosa.

### HPV-OPSCC displays parenchymal expansion and proliferative dysregulation

Analysis of keratin genes, well-established epithelial markers, revealed dichotomous expression across profiled tissues consistent with compartmentalization into tumor parenchyma, mucosal epithelium, and stroma (Fig. 1d and Supplementary Fig. 2a, b). To classify individual cells as either stromal or epithelial/parenchymal, we considered the proportion of keratin-positive cells among the nearest neighbors. The distribution of keratin-positivity over all cellular neighborhoods displayed bimodality, indicating spatially confined keratin expression (Fig. 1e). We classified individual cells with low or high proximal keratin-positivity as stromal or parenchymal cells, respectively, in agreement with histologic sample annotation and expression of epithelial and stromal marker genes (Fig. 1e, f, Supplementary Figs. 2a–c, 3). HPV-OPSCC samples were characterized by an increased number and proportion of cells classified as parenchymal compared to non-malignant mucosa, consistent with an expansion of the tumor parenchyma (Fig. 1g, Supplementary Fig. 2d).

HPV gene expression was evident in HPV-OPSCC parenchyma and largely undetectable in stroma and non-malignant mucosa (Fig.1h, i, and Supplementary Fig. 4a, b). Proliferation marker expression, which was restricted to a relatively thin layer proximal to the basement membrane in non-malignant mucosa, exhibited substantial delocalization across the HPV-OPSCC parenchyma (Fig. 1j, Supplementary Fig. 4a, b). Notably, HPV-OPSCC displayed a significantly greater proportion of proliferative cells in both stroma and parenchyma compared to the stroma and epithelium in non-malignant mucosa (Fig. 1k, Supplementary Fig. 4a–c). Thus, malignant HPV-OPSCC is characterized by expansion of the tumor parenchyma, widespread HPV gene expression, and proliferative dysregulation.

### Cell-type classification within single-cell SGE data

**Fig. 2:**
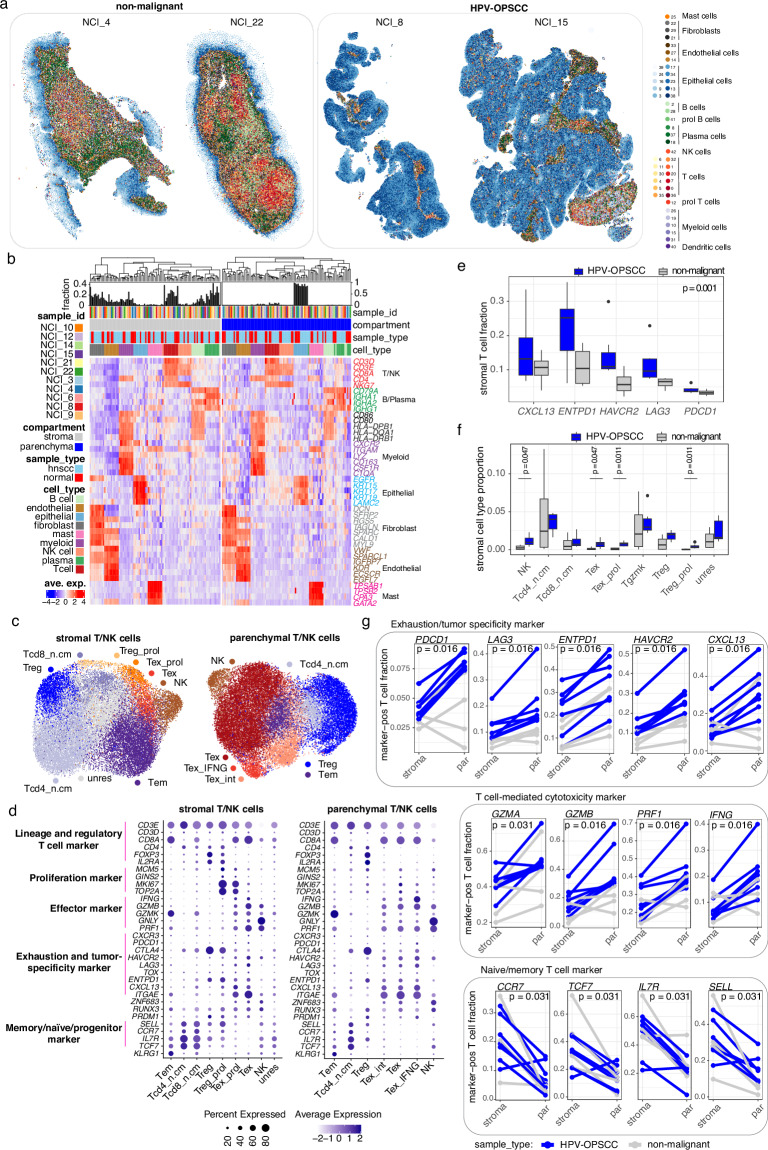
T cell markers of tumor specificity are enriched in the HPV-OPSCC tumor parenchyma. **a** Scatter plots showing cells within non-malignant epithelial (left) and HPV-OPSCC tissues (right). Colors reflect scRNA-seq-based cell-type classification. **b** Heatmap showing expression of cell-type marker genes within aggregate gene expression profiles of individual cell types resulting from scRNA-seq-based cell-type classification. For each sample, aggregate gene expression profiles were computed for each major cell type, considering stromal and parenchymal cells separately. Color corresponds to the scaled average expression. The top bar graph illustrates the fraction of a given cell type for each sample and compartment. **c** Scatter plots showing UMAP embedding of stromal (left) and parenchymal (right) T/NK cells identified within single-cell spatial gene expression data, colored by cluster identity. **d** Dot plots showing expression of select marker genes associated with T cell function for stromal (left) and parenchymal T/NK clusters shown in (**c**). For each gene, circle color and size correspond to scaled average expression and fraction of cells with non-zero expression, respectively. **e** Box plot showing the fraction of stromal T/NK cells with detectable expression of indicated genes within non-malignant epithelial (*n* = 4) and HPV-OPSCC (*n* = 7) tissues. The box corresponds to the interquartile range (IQR), the horizontal line inside the box indicates the median, whiskers extend to the smallest and largest data points within 1.5*IQR from the lower and upper quartiles, respectively, and data points beyond the boundaries of whiskers reflect outliers. *P*-values; two-way ANOVA. **f** A box plot showing the stromal proportions of indicated T/NK cell subsets within HPV-OPSCC (*n* = 7) and non-malignant epithelial (*n* = 4) samples. The box corresponds to the interquartile range (IQR), the horizontal line inside the box indicates the median, whiskers (vertical bars) extend to the smallest and largest data points within 1.5*IQR from the lower and upper quartiles, respectively, and data points beyond the boundaries of whiskers reflect outliers. *P*-value; two-sided Wilcoxon rank-sum test. Source data are provided as a Source Data file. **g** Line graphs showing fraction of stromal and parenchymal (par) T/NK cells with detectable expression of indicated genes within HPV-OPSCC (*n* = 7) and non-malignant epithelial (*n* = 4) samples. *P*-values; two-sided Wilcoxon rank-sum test using HPV-OPSCC samples.

To characterize the spatial distribution of distinct cell types across tissues, we generated custom reference gene expression profiles corresponding to each scRNA-seq cell-type cluster (Fig. 1b) to assign cell types to individual cells in the single-cell SGE data (Fig. 2a, Supplementary Fig. 5a). Expression of established lineage markers agreed with the resulting cell-type classification (Fig. 2b). HPV-OPSCC and non-malignant epithelium displayed clear differences related to epithelial cell-type classification possibly explained by variable proportions of malignant and non-malignant epithelial cells within individual scRNA-seq clusters as suggested by different HPV gene expression levels (Supplementary Fig. 5b). Keratin-high epithelial cells were enriched in regions classified as parenchyma/epithelium, and non-epithelial cell types such as myeloid cells and fibroblasts were detected more frequently in the stroma. We also observed focal aggregates of T and B/plasma cells reminiscent of germinal centers in the stroma of non-malignant mucosa and HPV-OPSCC (Fig. 2a).

To spatially characterize distinct myeloid phenotypes, 16,979 stromal and 15,931 parenchymal myeloid cells were clustered separately (Supplementary Fig. 6a–d). Graph-based clustering revealed distinct myeloid subsets that expressed marker genes of plasmacytoid dendritic cells (pDC), monocytes and macrophages (MoMa), or neutrophils (Neu). We observed a cluster that displayed a hybrid phenotype characterized by the expression of both MoMa and Neu marker genes (MoMa_Neu; Supplementary Fig. 6a, b), as observed by others^8,9^. Furthermore, we observed phenotypic variability in the MoMa populations reflected by differential expression of one or more genes, such as *MAFB*, *TREM2*, *CCR6,* or *IDO1*.

Next, we focused on the 29,549 stromal and 26,800 parenchymal T cells (Fig. 2c, Supplementary Fig. 6e, f). We identified regulatory T cell (Treg) clusters characterized by *CD4*, *FOXP3*, *IL2RA,* and *CTLA4* expression in both stroma and parenchyma, some of which exhibited expression of proliferation markers (Treg_prol; Fig. 2c, d). Naïve/central memory *CD4+* or *CD8* + T cells expressing *CCR7*, *IL7R,* and *TCF7* (Tcd4_n.cm; Tcd8_n.cm) were observed in stroma and parenchyma or stroma alone, respectively^10^ (Fig. 2c,d). A *CD8+* effector memory population (Tem) expressing *IL7R*, *TCF7*, *GZMK,* and *KLRG1* was evident in stroma and, albeit reduced, parenchyma (Fig. 2c, d). We observed distinct exhausted T cell subsets (Tex) that displayed expression of multiple co-inhibitory receptors such as *HAVCR2*, *LAG3*, *CTLA4,* and *PDCD1* and cytotoxic effector genes, such as *PRF1* and granzymes (Fig. 2c, d), indicative of tumor-reactivity^11–14^. Stromal and parenchymal Tex cell populations displayed variable expression of exhaustion (Tex_int) and proliferation markers (Tex_prol) or *IFNG* (Tex_IFNG; Fig. 2c, d). NK cells expressing *GNLY* were also present.

### Exhausted and regulatory T cell populations are associated with malignancy

Next, we evaluated immune-related differences between HPV-OPSCC and non-malignant mucosa. We observed a greater proportion of Tex and Tex_prol cells in HPV-OPSCC stroma compared to the mucosal stroma (Fig. 2e, f). Increased T cell exhaustion within the carcinoma-associated stroma was further supported by multiplex immunofluorescence, potentially reflecting enhanced T cell TCR engagement and activation (Supplementary Fig. 7). While NK and Treg_prol cells were also enriched in the HPV-OPSCC stroma, memory/naïve populations did not display significant differences between malignant and non-malignant stroma (Fig. 2f). The enrichment of proliferative T cell subsets may in part explain the increased proportion of proliferating cells within the HPV-OPSCC stroma (Fig. 1k). A similar comparison of parenchymal T cells revealed an enrichment of Tregs within HPV-OPSCC compared to non-malignant mucosal epithelium (Supplementary Fig. 6g, h).

We next assessed quantitative differences in T cell phenotype composition between HPV-OPSCC stroma and parenchyma. A greater proportion of T cells expressing exhaustion and effector genes was observed in the parenchyma compared to the stroma (Fig. 2g), suggesting that tumor-specific T cells are enriched in the tumor parenchyma. Genes associated with memory/naïve phenotypes exhibited higher expression in stromal T cells (Fig. 2g). These findings suggest that the tumor parenchyma, possibly through increased tumor antigen exposure and tumor-specific T cell TCR engagement, may enhance the development of the exhausted T cell phenotype.

### T and myeloid cell attracting chemokines display focal expression within the HPV-OPSCC parenchyma

**Fig. 3:**
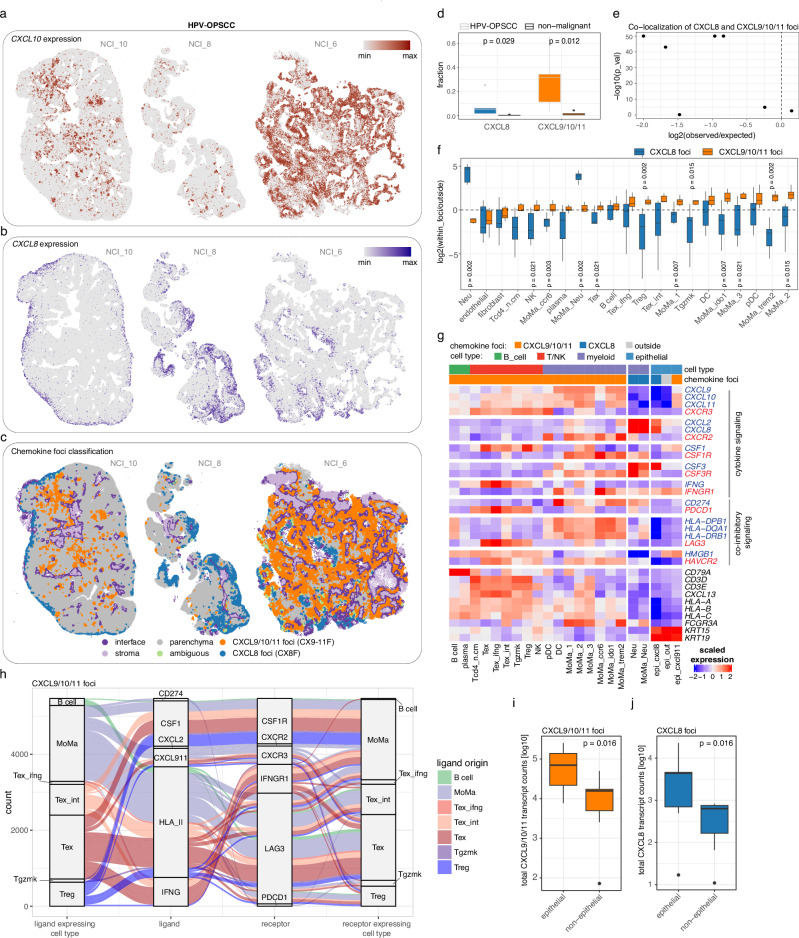
Chemokine foci underlie spatial immune cell heterogeneity in the tumor parenchyma. **a**–**c** Scatter plots showing three HPV-OPSCC tissues. Colors correspond to expression of CXCL10 (**a**), CXCL8 (**b**), or chemokine compartment classification (**c**). **d** A box plot showing the fraction of parenchymal cells classified as CXCL8 or CXCL9/10/11 foci for non-malignant epithelial (*n* = 4) and HPV-OPSCC (*n* = 7) tissues. Box corresponds to interquartile range (IQR), horizontal line inside the box indicates median, whiskers extend to the smallest and largest data points within 1.5*IQR from lower and upper quartiles, respectively, and data points beyond boundaries of whiskers reflect outliers. *P*-values; two-sided Wilcoxon rank-sum test. **e** A scatter plot showing the ratio of the observed and expected proportion of cells classified as both CXCL9/10/11 and CXCL8 foci and the corresponding *p*-values for each HPV-OPSCC sample. *P*-values; Fisher’s exact test. **f** Box plot showing the log2-transformed ratio of the frequencies of the indicated cell types within chemokine foci and the remaining parenchyma, i.e., parenchymal regions neither classified as CXCL8 nor CXCL9/10/11, for HPV-OPSCC tissues (*n* = 7). Box corresponds to interquartile range (IQR), horizontal line inside box indicates the median, whiskers extend to the smallest and largest data points within 1.5*IQR from the lower and upper quartiles, respectively, and data points beyond boundaries of whiskers reflect outliers. *P*-values; two-sided Wilcoxon rank-sum test. Source data are provided as a Source Data file. **g** Heatmap showing expression of genes within aggregate gene expression profiles corresponding to cells assigned to indicated cell types and chemokine foci. Gene names of ligands and receptors of select cytokine and co-inhibitory signaling pathways are shown in blue and red, respectively. Color corresponds to row-scaled average expression. **h** Alluvial diagram corresponding to all immune cell pairs that express cognate receptor-ligand pairs and satisfy a nearest neighbor relationship. More specifically, for each ligand-expressing cell, a k-nearest neighbor graph was computed (*k* = 5) and used to identify cognate receptor-expressing neighboring cells. The width of the alluvia/flows between the first and second axes or the third and fourth axes represents the number of cells of a given cell type that express a specific ligand or receptor, respectively. The alluvia between the second and third axes corresponds to the number of instances the indicated ligand/receptor-expressing cells localize in close proximity to satisfy the nearest neighbor relationship. Box plots showing the cumulative CXCL9/10/11 (**i**) and CXCL8 (**j**) transcript counts associated with epithelial and non-epithelial cells within CXCL9/10/11 (**i**) or CXCL8 foci (**j**) of HPV-OPSCC tissues (*n* = 7). The box corresponds to the interquartile range (IQR), the horizontal line inside the box indicates the median, whiskers extend to the smallest and largest data points within 1.5*IQR from the lower and upper quartiles, respectively, and data points beyond the boundaries of whiskers reflect outliers. *P*-values; two-sided Wilcoxon rank-sum test.

Despite stromal enrichment, a subset of lymphocytes and myeloid cells infiltrated the HPV-OPSCC parenchyma (Fig. 2b-c, Supplementary Fig. 6a). To better understand this process, we evaluated parenchymal expression of chemokines *CXCL9, CXCL10,* and *CXCL11* (*CXCL9/10/11*) that mediate CXCR3-dependent T cell chemotaxis. We observed localized/focal, as opposed to uniform, *CXCL9/10/11* expression within the HPV-OPSCC parenchyma (Fig. 3a, Supplementary Fig. 8a). This focal *CXCL9/10/11* expression varied in magnitude and pattern across HPV-OPSCC samples. In addition, parenchymal expression of the chemokine *CXCL8*, involved in the CXCR2-dependent recruitment of neutrophils and mononuclear myeloid cells^15^, also displayed variable, localized, non-uniform patterns (Fig. 3b, Supplementary Fig. 8a). Based on chemokine expression of neighboring cells, we assigned individual cells to *CXCL8* or *CXCL9/10/11* foci (Fig. 3c, Supplementary Figs. 8a, b and 9a, b). We observed substantial variability in chemokine foci extent across HPV-OPSCC samples (Supplementary Fig. 9c). Notably, focal chemokine expression was largely absent in non-malignant mucosal epithelium, suggesting that formation of chemokine foci is a pathologic feature in HPV-OPSCC (Fig. 3d, Supplementary Fig. 9d, e). *CXCL8* and *CXCL9/10/11* foci predominantly displayed spatial separation and mutual exclusivity across HPV-OPSCC samples (Fig. 3e). Consistently, *CXCL9/10/11* foci were generally close to or associated with the stroma, whereas *CXCL8* foci frequently formed in areas of the parenchyma further from the stroma (Fig. 3c, Supplementary Figs. 8b and 9f).

We next studied cell-type composition within chemokine foci. Most cells within both *CXCL8* and *CXCL9/10/11* foci were tumor cells (Supplementary Fig. 9g). We compared frequencies of non-tumor cell types within chemokine foci to frequencies observed in the remaining parenchyma (neither *CXCL8* nor *CXCL9/10/11* foci). This revealed that various monocyte/macrophage and T cell subsets were enriched within *CXCL9/10/11* foci and depleted in *CXCL8* foci (Fig. 3f). In contrast, Neu and MoMa_Neu neutrophilic cells were significantly enriched within *CXCL8* foci. To characterize intercellular signaling events within chemokine foci, we evaluated expression of different ligands and their cognate receptors across different cell types and parenchymal compartments (Fig. 3g). We observed relatively high expression of *CXCL9/10/11* by mononuclear myeloid and tumor cells within *CXCL9/10/11* foci, while the cognate receptor *CXCR3* was predominantly expressed on T cell subsets as expected^16^. *CSF1* expression was relatively high in multiple T cell subsets, while *CSF1R* was expressed in MoMa cells, suggesting that T and mononuclear myeloid cell accumulation in *CXCL9/10/11* foci may result from reciprocative production of attracting chemokines. In contrast, expression of *CXCL8* and *CXCL2*, encoding CXCR2 ligands, was largely restricted to neutrophilic and epithelial cells within *CXCL8* foci; elevated *CXCR2* expression was observed in *CXCL8*-foci neutrophils and *CXCL9/10/11*-foci myeloid cells, potentially indicating interactions between *CXCL8* and *CXCL9/10/11* foci. *CSF3* expression was highest in *CXCL8*-foci neutrophilic and tumor cells, and the cognate receptor *CSF3R* was expressed at relatively high levels on *CXCL8*-foci Neu and MoMa_Neu cells, potentially indicating a role of CSF3 in chemotaxis of neutrophilic cells within *CXCL8* foci (Fig. 3g).

Variable *IFNG* expression was observed across T cell clusters with an exhausted phenotype (Fig. 3g). In contrast, the receptor *IFNGR1* was predominantly expressed in different myeloid and tumor cells, supporting a role for IFN-γ in the induction of *CXCL9/10/11* gene expression and formation of *CXCL9/10/11* foci. We also evaluated expression of co-inhibitory receptors and their cognate ligands. *PDCD1* and *LAG3* exhibited the greatest relative expression on T cell subsets, while their respective ligands *CD274* and HLA class II displayed the highest expression on myeloid cells, indicating a potentially immunosuppressive role of a subset of myeloid cells in *CXCL9/10/11* foci.

We also considered potential direct cell-to-cell and paracrine signaling interactions by analyzing ligand-receptor expression on spatially proximal immune cells within the distinct chemokine foci. This analysis revealed a complex regulatory landscape within *CXCL9/10/11* foci (Fig. 3h). Notably, co-localization of HLA class II-expressing MoMa cells and *LAG3*-expressing T cells was observed in numerous instances and at a greater frequency than *CD274*-expressing myeloid cells and *PDCD1*-expressing T cells, suggesting that LAG3-mediated signaling may be a dominant mechanism of T cell suppression in HPV-OPSCC. Consistently, HLA class II-expressing carcinoma cells displayed increased spatial association with different T cell subsets (Supplementary Fig. 10a). *CSF1*-expressing T cells were detected near *CSF1R*-expressing MoMa cells and *IFNG* produced by T cells may affect proximal *IFNGR1*-expressing MoMa cells (Fig. 3h). Further supporting these findings, similar ligand-receptor interactions were also identified within scRNA-seq data (Supplementary Fig. 10b). In contrast, the number of potential proximal signaling interactions was substantially reduced in *CXCL8* foci and the remaining parenchyma (Supplementary Fig. 10b, c), possibly reflecting reduced immune cell density and diversity.

We also evaluated absolute cell-type-specific contributions of chemokine transcripts to the chemokine foci. Consistent with the large proportion of epithelial tumor cells within chemokine foci (Supplementary Fig. 9g), we observed the greatest absolute contribution of chemokine transcripts from epithelial tumor cells in both *CXCL8* and *CXCL9/10/11* foci (Fig. 3i, j), suggesting that, in addition to different immune cell populations^17–19^, cancer cells produce substantial amounts of chemokines and play an important role in the formation of these foci.

### Cancer cells within CXCL9/10/11 and CXCL8 foci express conserved gene programs

**Fig. 4:**
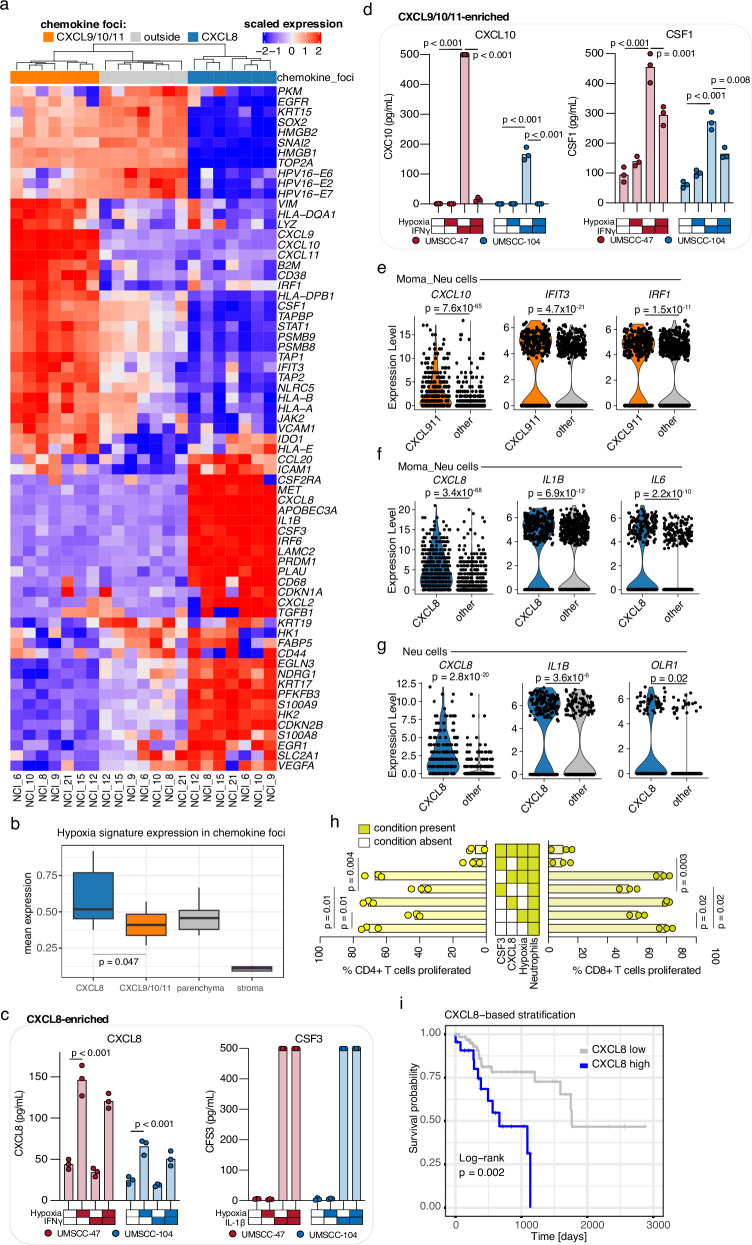
Gene expression characteristics associated with chemokine foci. **a** Heatmap showing expression of differentially expressed genes within aggregate gene expression profiles corresponding to epithelial cells associated with different chemokine foci. Color corresponds to average gene expression scaled within each sample. **b** Box plot showing the mean expression of a hypoxia signature within HPV-OPSCC samples (*n* = 7) for the indicated chemokine and anatomical compartments. The box corresponds to the interquartile range (IQR), the horizontal line inside the box indicates the median, whiskers (vertical bars) extend to the smallest and largest data points within 1.5*IQR from the lower and upper quartiles, respectively, and data points beyond the boundaries of whiskers reflect outliers. *P*-value; two-sided Wilcoxon rank-sum test. Bar graphs showing expression of soluble CXCL8 and CSF3 (**c**) or CXCL10 and CSF1 (**d**) in patient-derived HPV-positive cell lines UM-SCC-47 and UM-SCC-104 treated with a combination of hypoxia and IFN-γ or IL-1β as indicated below using colored rectangles in three biological replicates. *P*-values; ANOVA with multiple comparisons. **e** A violin plot showing expression of select differentially expressed genes resulting from comparison of MoMa-Neu cells within CXCL9/10/11 foci to remaining parenchymal MoMa-Neu cells. *P*-values; Wilcoxon rank-sum test; adjusted for multiple testing. **f**, **g** Violin plot showing expression of select differentially expressed genes resulting from comparison of MoMa-Neu cells within CXCL8 foci to remaining parenchymal MoMa-Neu cells (**g**) or Neu cells within CXCL8 foci to remaining parenchymal Neu cells (**i**). *P*-values; two-sided Wilcoxon rank-sum test; adjusted for multiple testing. **h** A bar graph showing the fraction of activated CD4+ and CD8 + T cells undergoing proliferation during co-culture with healthy donor neutrophils. Prior to co-culture with T cells, neutrophils were treated with different combinations of hypoxia, CXCL8, and CSF3 as indicated below using yellow rectangles. *P*-values are based on paired two-sided Wilcoxon rank-sum test; *: *p* ≤ 0.05; **: *p* ≤ 0.01. **i** Line graph showing survival probabilities over time resulting from *CXCL8* expression-based stratification of TCGA HPV-HNSCC samples. *P*-values are based on the log-rank test.

To further study tumor-cell-intrinsic mechanisms contributing to chemokine foci formation, we identified genes that are differentially expressed between cancer cells associated with different chemokine foci. Gene expression-based clustering, using differentially expressed genes, revealed a clear and consistent separation of cancer cell expression profiles by chemokine foci as opposed to patient identity (Fig. 4a), suggesting that features underlying the formation of these chemokine foci are conserved across patients. Tumor-cell HPV gene expression was substantially reduced in *CXCL8* foci. Similarly, *SNAI2*, *EGFR*, *SOX2,* and *TOP2A* were highly expressed outside of chemokine foci, potentially indicating enhanced epithelial-to-mesenchymal transition and proliferative capacity of these cells. *CXCL9/10/11*-foci cancer cells expressed relatively high levels of IFN-γ-response genes involved in antigen processing and presentation, such as *TAP2*, *TAPBP*, *B2M, HLA* genes, potentially reflecting increased levels of IFN-γ in this compartment. In contrast, *CXCL8*-foci cancer cells were characterized by relatively high expression of *CXCL8*, *CSF3*, *CXCL2*, *CCL2*, and *IL1B* (Fig. 4a). Furthermore, we observed reduced expression of HPV genes *E6*, *E7*, cell-cycle gene *TOP2A* and a concomitant increase in expression of cyclin-dependent kinase inhibitors *CDKN1A*/p21 and *CDKN2B*/p15, potentially indicating reduced viral gene activity and increased cell-cycle arrest. Increased expression of *CDKN1A*, a direct target of p53-mediated transcriptional activation, may reflect increased p53 activity in *CXCL8*-foci cancer cells^20^. In addition, we observed elevated expression of hypoxia-inducible genes *VEGFA* and *EGLN3* and glycolysis-related genes *HK2* and *PFKFB3*, suggesting that *CXCL8* foci may be predominantly embedded in hypoxic regions of the parenchyma^21–23^. Consistently, cells within *CXCL8* foci were characterized by greater expression of gene signatures related to hypoxia and glycolysis (Fig. 4b, Supplementary Fig. 11a). In agreement with the single-cell SGE data, analysis of HPV-OPSCC scRNA-seq data revealed increased IFN-γ signature expression within *CXCL9/10/11*-positive epithelial cells, while *CXCL8* expression was associated with reduced cell-cycle scores and elevated hypoxia signature levels (Supplementary Fig. 11b–e). Importantly, analysis of copy number alterations in tumor cells within distinct chemokine foci did not reveal a clear association between clonal structure and foci classification (Supplementary Fig. 11b), suggesting that chemokine foci development is likely driven by physicochemical TME features, such as hypoxia and IFN-γ, as opposed to spatially clustered cancer cell clones.

### Hypoxia and IFN-γ induce gene expression patterns characteristic of *CXCL8* and *CXCL9/10/11* foci, respectively

To further characterize potential mechanisms underlying the observed cancer cell gene expression differences between chemokine compartments, we studied two patient-derived p53-wild-type HPV-OPSCC cell lines^24^. When cultured under hypoxic conditions, both cell lines displayed significant upregulation of secreted CXCL8 (Fig. 4c), suggesting that hypoxia contributes to the formation of *CXCL8* foci. In contrast, expression and secretion of CSF3, another cytokine associated with *CXCL8* foci, was unaffected by hypoxia (Supplementary Fig. 11g), but induced upon exposure to IL-1β (Fig. 4c), also highly expressed in *CXCL8* foci. In contrast, expression of CXCL10 and CSF1 was induced by IFN-γ (Fig. 4d). IFN-γ-induced CXCL10 and CSF1 were significantly reduced in hypoxic conditions, suggesting that hypoxia may antagonize the formation of *CXCL9/10/11* foci. Similarly, HLA class I and II were upregulated in response to IFN-γ, but significantly reduced in the presence of hypoxia, consistent with reduced HLA class I and II gene expression in *CXCL8* foci (Supplementary Figs. 11h, 4a). In addition, analysis of multiple independent published HNSCC spatial transcriptomic data sets further supports the link between hypoxia and IFN-γ signaling in the formation of *CXCL8* and *CXCL9/10/11* foci, respectively (Supplementary Fig. 12a–c). Collectively, these results suggest that physicochemical conditions in the TME, namely hypoxia and IFN-γ, may play important and possibly contrasting roles in the development of *CXCL8* or *CXCL9/10/11* foci.

### Conditions within *CXCL8* foci drive the development of immunosuppressive myeloid cells and predict reduced overall survival in patients with HPV-OPSCC

We next focused on MoMa_Neu and Neu cells located within different chemokine foci. Differential gene expression analysis revealed elevated expression of multiple IFN-γ response genes within *CXCL9/10/11*-foci MoMa_Neu cells (Fig. 4e), including *IRF1*, which has recently been implicated in neutrophil-mediated tumor control^25^. In contrast, MoMa_Neu and Neu cells located within *CXCL8* foci exhibited elevated expression of genes associated with immunosuppression, such as *IL1B*, *IL6,* and *OLR1* (Fig. 4f, g)^9,26,27^. Potent suppression of T cell proliferation by neutrophilic cells isolated from an independent cohort of HPV-OPSCC tumors was observed (Supplementary Fig. 13a, b). To explore conditions within *CXCL8* foci that may promote neutrophil-mediated immunosuppression, we assessed T cell suppressive capacity of healthy donor blood neutrophils following exposure to hypoxia, CXCL8, and CSF3 alone or in combination. While exposure to CXCL8 alone did not enhance immunosuppressive capacity in neutrophiles, exposure to hypoxia or CSF3 was sufficient to induce a modestly immunosuppressive state (Fig. 4h). Furthermore, neutrophils concurrently exposed to hypoxia and CSF3 displayed marked T cell suppressive capacity (Fig. 4h). Consistently, T cells within *CXCL8* foci displayed a significant reduction in proliferative proportion compared to other regions within the parenchyma (Supplementary Fig. 13c). To further explore the clinical significance of distinct chemokine foci, we analyzed additional HPV-HNSCC samples from The Cancer Genome Atlas (TCGA). Correlation analysis of chemokine expression agreed well with expression patterns within our SGE data (Supplementary Fig. 13d, e). Furthermore, we did not observe a correlation between *CXCL10* and *CXCL8* expression within the HPV-HNSCC TCGA cohort, potentially indicating independent formation of different chemokine foci classes (Supplementary Fig. 13f). Notably, chemokine expression-based survival analysis showed that elevated expression of both *CXCL8* and *CXCL2*, alone or in combination, associates with reduced overall survival in HPV-HNSCC (Fig. 4i, Supplementary Fig. 13g). These findings indicate that conditions within *CXCL8* foci may interfere with effective anti-tumor immunity through the recruitment of neutrophils that undergo polarization toward an immunosuppressive state.

### Hypoxia and IFN-γ contribute to the formation of the HPVoff phenotype

**Fig. 5:**
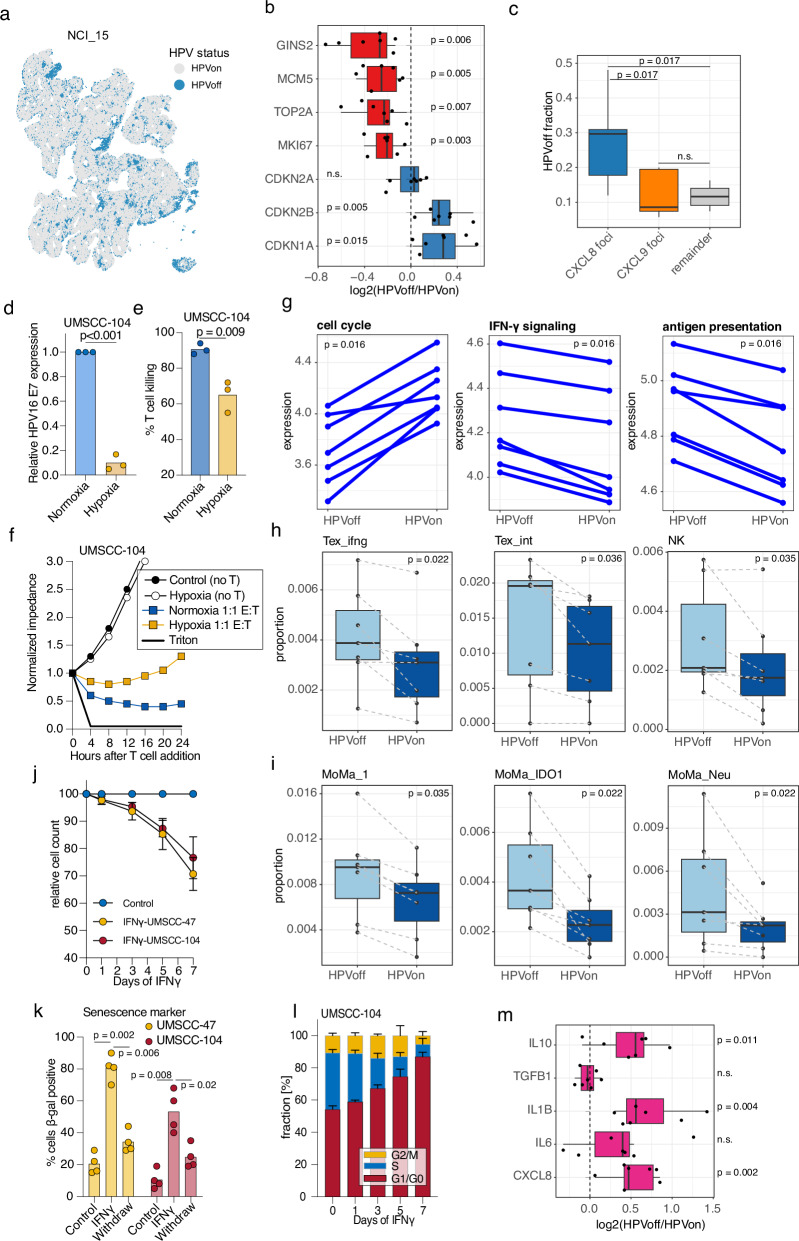
Hypoxia and IFN-γ promote the induction of characteristics associated with the HPVoff phenotype. **a** Scatter plot showing cells of a representative HPV-OPSCC sample. Colors reflect detection of HPV transcripts. **b** Box plot showing fold change of indicated pro- and anti-proliferative genes comparing average expression in HPVoff and HPVon cells located within neither CXCL9/10/11 nor CXCL8 foci for each HPV-OPSCC sample (*n* = 7). The box corresponds to the interquartile range (IQR), the horizontal line inside the box indicates the median, whiskers extend to the smallest and largest data points within 1.5*IQR from the lower and upper quartiles, respectively, and data points beyond the boundaries of whiskers reflect outliers. *P*-values; two-sided Student’s *t*-test. Source data are provided as a Source Data file. **c** Box plot showing the fraction of HPVoff cells within distinct parenchymal compartments within HPV-OPSCC samples (*n* = 7). The box corresponds to the interquartile range (IQR), the horizontal line inside the box indicates the median, whiskers extend to the smallest and largest data points within 1.5*IQR from the lower and upper quartiles, respectively, and data points beyond the boundaries of the whiskers reflect outliers. *P*-values; two-sided Wilcoxon rank-sum test. Source data are provided as a Source Data file. Bar graphs showing HPV16E7 expression under normoxia and hypoxia (**d**) and quantification of T cell-mediated killing of UM-SCC 104 cells following 16 h of T cell and target cell co-culture (**e**). *P*-values; two-tailed *t*-test. **f** Line graphs showing normalized impedance of patient-derived HPV-OPSCC cell line UM-SCC-104, co-cultured with HPV16E7-specific T cells. UM-SCC-104 cells were cultured under normoxic or hypoxic conditions, prior to T cell co-culture under normoxic conditions. E:T Effector-target ratio; no T no T cells. **g** Line graphs showing average expression of cell-cycle (left), IFN-γ signaling (center), and antigen presentation (right) gene signatures in HPVon and HPVoff HPV-OPSCC epithelial cells (*n* = 7). *P*-values; two-sided, paired Wilcoxon rank-sum test. Box plots showing the proportions of Tex_infg (left), Tex_int (center), and NK cells (right; **h**) or MoMa_1 (left), MoMa_IDO1 (center), and MoMa_Neu cells (right; **i**) within nearest neighbors of HPVoff and HPVon cells. For each HPVon and HPVoff cell, the five nearest neighbors were considered. HPVon and HPVoff cells corresponding to the same HPV-OPSCC sample (*n* = 7) are connected by dashed lines. The box corresponds to the interquartile range (IQR), the horizontal line inside the box indicates the median, whiskers extend to the smallest and largest data points within 1.5*IQR from the lower and upper quartiles, respectively, and data points beyond the boundaries of the whiskers reflect outliers. *P*-values; two-sided Wilcoxon rank-sum test. **j** Line graph showing mean relative cell count of UM-SCC-47 and UM-SCC-104 treated with IFN-γ in three biological replicates. Error bars correspond to the standard deviation. **k** Bar graphs showing fraction of cells expressing β-galactosidase in UM-SCC-47 and UM-SCC-104 cell lines, untreated, following 7 days of IFN-γ treatment, and 7 days post-IFN-γ withdrawal in four biological replicates. *P*-values: paired, two-sided Wilcoxon rank-sum test. **l** The bar graph showing the mean proportions of cells within indicated phases of the cell cycle in UM-SCC-104 cells subjected to increasing IFN-γ treatment durations using three biological replicates. Error bars correspond to the standard deviation. **m** The box plot shows fold change of indicated cytokine genes comparing average expression in HPVoff and HPVon cells located within neither CXCL9/10/11 nor CXCL8 foci for each HPV-OPSCC sample (*n* = 7). The box corresponds to the interquartile range (IQR), the horizontal line inside the box indicates the median, whiskers extend to the smallest and largest data points within 1.5*IQR from the lower and upper quartiles, respectively, and data points beyond the boundaries of whiskers reflect outliers. *P*-values; two-sided Student’s *t*-test. Source data are provided as a Source Data file.

HPV-OPSCC cells characterized by a lack of detectable HPV gene expression (HPVoff), reduced cell-cycle gene expression, and a concomitant upregulation of an epithelial senescence signature were recently described^6^. Using our single-cell SGE data, we detected HPVoff cells in all HPV-OPSCC samples at varying proportions (Fig. 5a, Supplementary Fig. 14a, b). Consistently, HPVoff cells displayed a substantial reduction in proliferative proportion (Supplementary Fig. 14c), reduced cell-cycle gene expression and increased expression of CDK inhibitors, established markers of cellular senescence (Fig. 5b). HPVoff cells also displayed a tendency toward reduced HLA class II expression, consistent with previous observations^28^ (Supplementary Fig. 14d). Spatial analysis revealed a significantly greater proportion of HPVoff cells within *CXCL8* foci (Fig. 5c). The enrichment of HPVoff cells within *CXCL8* foci is further supported by our scRNA-seq analysis (Supplementary Fig. 14e). Considering the increased hypoxia gene signature expression within CXCL8 foci, we evaluated HPV gene expression under hypoxic conditions using two patient-derived HPV16-positive cancer cell lines. In both cell lines, HPV16E7 expression was significantly reduced in hypoxic conditions, suggesting that hypoxia likely contributes to the HPVoff phenotype (Fig. 5d and Supplementary Fig. 14f).

To evaluate the effects of hypoxia on tumors in the context of T cell-mediated tumor-cell cytotoxicity^29^, we co-cultured two HPV16-positive HPV-OPSCC cancer cell lines with HPV16E7-specific TCR-engineered T cells. Although substantial tumor-cell killing was observed under normoxic conditions, hypoxic conditioning of tumor cells prior to co-culture with T cells led to a significant reduction in tumor-cell killing (Fig. 5e, f, Supplementary Fig. 14g–i). These results suggest that hypoxia may directly promote evasion of HPV-specific T cell immunity, possibly through the combination of hypoxia-induced reduction in HPV gene expression and reduced tumor-cell HLA class I.

To further assess how HPV gene expression status may affect tumor-immune cell interactions, we focused on HPVon and HPVoff cells within *CXCL9/10/11* foci that are enriched in immune cells. Compared to HPVon cells, HPVoff cells within *CXCL9/10/11* foci displayed significantly reduced expression of cell-cycle and apoptosis gene signatures (Fig. 5g, Supplementary Fig. 14j), indicative of senescence^30^. In contrast, signatures associated with IFN-γ signaling and antigen presentation were significantly elevated in HPVoff cells, suggesting that HPVoff cells within *CXCL9/10/11* foci are exposed to increased IFN-γ levels (Fig. 5g, Supplementary Fig. 14k). A trend toward increased IFN-γ signaling signature expression within HPVoff cells was also seen in scRNA-seq data (Supplementary Fig. 14l). Consistent with these observations, IFN-γ-mediated inhibition of viral gene expression and replication has been described^31–34^. Notably, HPVoff cells within *CXCL8* foci did not display characteristics of increased IFN-γ signaling, further supporting the idea that different mechanisms may give rise to HPVoff cells (Supplementary Fig. 14k, m), potentially further affected by distinct differentiation states (Supplementary Fig. 14n).

We also analyzed the local cellular neighborhoods of both HPVon and HPVoff cells within *CXCL9/10/11* foci. Consistent with the elevated IFN-γ signature expression, HPVoff cells displayed increased spatial association with different T/NK cell subsets, including *IFNG-*expressing Tex_ifng cells that may contribute to the HPVoff phenotype (Fig. 5h). Different MoMa subsets also displayed significant enrichment within HPVoff neighborhoods (Fig. 5i). To investigate the relationship between IFN-γ exposure and cellular senescence, we treated HPV-OPSCC cancer cell lines with IFN-γ for up to seven days. IFN-γ treatment led to a progressive reduction in cell proliferation and a concurrent upregulation of the senescence marker β-galactosidase (Fig. 5j, k, Supplementary Fig. 15a). Cell-cycle analysis further revealed time-dependent G_0_/G_1_ cell-cycle arrest (Fig. 5l, Supplementary Fig. 15b–d). Notably, IFN-γ treatment of two HPV-negative, p53-mutant HNSCC cell lines lacking p53 protein^35^ did not lead to cell-cycle arrest or upregulation of senescence markers, suggesting that IFN-γ-induced cell-cycle arrest may depend on p53 function (Supplementary Fig. 15e–h). Furthermore, we observed that withdrawal of IFN-γ led to substantially reduced β-galactosidase positivity and recovered proliferation (Fig. 5k, Supplementary Fig. 15i), possibly indicating reversibility of IFN-γ-induced senescence within HPV-OPSCC cancer cells. Together, these data suggest that cancer cell exposure to chronic IFN-γ may also contribute to the HPVoff phenotype through induction of a senescent-like state. Senescent cells can have variable effects on tumor progression^30^. A hypersecretory state, referred to as senescence-associated secretory phenotype (SASP), has been associated with cancer cell stemness, angiogenesis, and immune evasion^30,36–38^. To assess the immunomodulatory potential of HPVoff cells, we evaluated expression of different immunosuppressive and SASP-associated cytokines. Interestingly, multiple cytokines displayed significantly higher expression in HPVoff cells (Fig. 5m), suggesting that HPVoff cells are capable of modulating anti-tumor immunity using both cell-intrinsic and extrinsic mechanisms.

## Discussion

Our analysis revealed detailed insights into cellular order associated with HPV-OPSCC. We describe malignant and immune cell types and cellular states at high resolution within their spatial context across distinct anatomical compartments and states of malignancy.

Expression analysis of T and myeloid cell attracting chemokines revealed spatially distinct expression foci distributed across the tumor parenchyma that were largely absent in non-malignant mucosa. *CXCL9/10/11* foci were enriched in different T and mononuclear myeloid cell populations, exhibited transcriptional features of elevated IFN-γ signaling, and displayed increased potential of direct cell-to-cell and paracrine signaling interactions, similar to so-called ‘immune hubs’ described in colorectal^39^ and lung cancer^40^. In contrast, *CXCL8* foci occupied separate regions of the tumor parenchyma, were enriched in neutrophilic myeloid populations harboring increased immunosuppressive capacity, were distant from the stroma, and expressed markers of hypoxia. The mechanisms underlying the selective enrichment of distinct myeloid cell populations within both *CXCL8* and *CXCL9/10/11* foci are incompletely understood and may involve combinatorial signaling of distinct chemokine receptors. For instance, relatively low expression of *CXCR2* on neutrophilic myeloid cells within *CXCL8* foci suggests that additional chemotactic signaling pathways, such as CSF3/CSF3R, may be required for the focal accumulation. Similarly, accumulation of myeloid cells within *CXCL9/10/11* foci despite expression of *CXCR2* may indicate the involvement of other chemokine receptors, such as CSF1R, in this process. In addition, chemokine receptor transcript counts may not accurately reflect effective cell-surface receptor protein levels due to post-translational regulation. Further study of the mechanisms underlying selective enrichment of distinct myeloid populations within different tumor-parenchymal regions, for instance, using protein-level analysis, is warranted. Furthermore, concurrent expression of ligands (*CXCL2*, *CXCL8,* and *CSF3*) and cognate receptors (*CXCR2* and *CSF3R*) on neutrophilic cells within *CXCL8* foci could indicate autocrine signaling and self-recruitment potential. Analysis of TCGA data revealed that elevated intratumoral expression of both *CXCL2* and *CXCL8* was predictive of reduced overall survival. Consistently, higher serum CXCL8 levels have been associated with reduced overall survival in patients with HPV-associated cancers^41^. If focal *CXCL8* expression within the tumor parenchyma contributes to serum CXCL8 requires further evaluation. Our analysis suggests that *CXCL8* and *CXCL9/10/11* foci may differ regarding intrinsic immunosuppressive mechanisms. For instance, within *CXCL9/10/11* foci, immunosuppression of T cells may predominantly depend on stimulation of co-inhibitory receptors, such as LAG3 or PD-1. In contrast, hypoxic conditions present within *CXCL8* foci may preferentially support the development of immunosuppressive neutrophilic cells. Whether the reduction of hypoxia levels, for instance, through cytoreductive therapy or LAG3 blockade, could result in clinical benefit in HPV-OPSCC requires further investigation. Mechanistic studies designed to further assess productive proximal and distal signaling events affecting individual immune cells are warranted. Similarly, events underlying the initiation of chemokine foci formation require further analysis. Tumor cells located within distinct chemokine foci displayed similarities in gene expression across patients, suggesting that functional properties of *CXCL8* and *CXCL9/10/11* foci are conserved.

Analysis of HPV-positive cell lines suggested that hypoxia and IFN-γ exposure promote expression of a subset of *CXCL8* and *CXCL9/10/11* foci defining factors, respectively, and implicate antagonistic effects of hypoxia and IFN-γ in the mutual exclusivity of these chemokine foci.

Carcinoma cells that lack expression of HPV genes (HPVoff) were evident across the tumor parenchyma. HPVoff cells displayed reduced expression of cell-cycle genes and a concomitant upregulation of senescence markers, consistent with previous observations^6^. Spatial analysis revealed an enrichment of HPVoff cells within *CXCL8* foci. In agreement with these observations, hypoxic conditions are sufficient to reduce HPV gene expression in vitro. Analysis of HPVoff cells within *CXCL9/10/11* foci revealed spatial interactions with IFN-γ-producing T cells, in addition to other immune cell subsets, potentially representing an alternative mechanism underlying induction of the HPVoff phenotype. Consistently, different studies have demonstrated the ability of IFN-γ to mediate repression of viral gene expression^32,33^. Whether cell-intrinsic factors, such as stochastic HPV gene expression or epigenetic predisposition, can promote the HPVoff phenotype requires further study. Furthermore, prolonged exposure of HPV-OPSCC cell lines to hypoxia led to reduced susceptibility to HPV-specific T cell-mediated cytotoxicity, consistent with a significant reduction in HPV gene and MHC class I and II expression. Therefore, it is possible that the HPVoff phenotype provides protection from anti-tumor immunity specifically directed against HPV-associated antigens, reminiscent of an adaptive immune resistance mechanism^42^. How long HPVoff cells retain presentation of immunogenic HPV antigens following termination of HPV gene transcription in vivo requires additional analysis. Studies that evaluate whether similar mechanisms exist in the context of other high-risk HPV types, benign low-risk HPV-associated tumors^43^ or other virus-associated malignancies are warranted^44,45^.

The importance of the HPV E6 target p53 in the induction of cellular senescence and the stability of the HPVoff phenotype in vivo requires further analysis. Selective abrogation of p53 signaling in senescent cells can induce spontaneous escape from senescence^46,47^. Therefore, it is conceivable that reactivation of HPV gene expression in HPVoff cells could reinstate p53 inactivation and result in escape from cell-cycle arrest.

In addition to tumor control mechanisms related to the induction of cell-cycle arrest, the senescent state has also been implicated in promoting tumorigenesis^30,38^. In our dataset, HPVoff cells were characterized by increased expression of various cytokines, such as *IL6*, *IL1B,* and *IL10*, previously implicated in immunosuppression^26,27,48^. A hypersecretory state referred to as senescence-associated secretory phenotype (SASP) has been described^36^ and associated with adverse outcomes in viral infections^49,50^ and cancer^51^. Whether HPVoff cells can promote T cell exhaustion, for instance, through production of immunosuppressive cytokines, requires further analysis. Similarly, whether patients with HPV-OPSCC could benefit from selective depletion of HPVoff cells through the use of senolytic therapy^38^ warrants further investigation.

Limitations to spatial gene expression analysis exist. Low transcript counts in SGE can also complicate cell type and state analysis. Probe-based quantification of gene expression in SGE data may be affected by characteristics of transcripts and probes and, therefore, may contain bias, such as over- or underestimation of actual transcript counts and off-target binding. Despite restricting our single-cell SGE analysis to nuclear transcripts, SGE quantification may be affected by misassigned transcripts. Development of more accurate cellular segmentation methods to allow quantification of transcripts across entire cells is warranted.

In summary, our analysis highlights a complex interplay between different cell types and states underlying the spatial cellular organization of HPV-OPSCC with implications for heterogeneous cancer cell states, immunosuppression, and immune evasion.

## Methods

### Human specimen collection and processing

This study complies with all relevant ethical regulations. Clinical (tissue) specimens were collected from patients with newly diagnosed, previously untreated HPV-related OPSCC referred to the NIH Clinical Center for consideration of clinical trials or from patients undergoing standard of care procedures following verbal and written informed consent. The biospecimen protocol under which these specimens were collected was reviewed and approved by the National Institutes of Health Clinical Center Institutional Review Board (NCT03429036). Deidentified healthy donor PBMC were acquired from the NIH Blood Bank. Demographic characteristics of patients providing OPSCC samples for this study are summarized in Supplementary Tables 1 and 2. Patient carcinomas were determined to be HPV-associated based on p16 immunohistochemistry positivity in a CLIA-certified clinical laboratory prior to referral, and HPV positivity was confirmed via laboratory-based RT-PCR following biopsy as described below. Whole-exome sequencing was performed on carcinoma biopsies using the commercial service provided by Caris. Fresh carcinoma biopsies were divided and snap frozen for the extraction of genomic material for HPV typing, fixed in formalin for histologic assessment and spatial transcriptomic analysis, or freshly digested into a single-cell suspension for single-cell RNA-sequencing as described below. Non-malignant mucosa adjacent to carcinoma was pathologically annotated and used for comparison.

### HPV typing of clinical specimens

Snap frozen carcinoma samples were thawed, disrupted, and homogenized using the TissueLyser System (QIAGEN), and gDNA was extracted using the QIAamp DNA Mini Kit (QIAGEN) per manufacturer recommendations. Real-time PCR was performed in a reaction mixture of 5 µL gDNA (20 ng/µL), 10 µL TaqMan Universal Master Mix II with UNG (2×, ABI Cat#4426710), 1 µl Custom TaqMan Gene Expression Assay (20× Real-time PCR Primer/probe set, Thermo Fisher Scientific Cat# 4331348, which consist of HPV16L forward primer: TTGTTGGGGTAACCAACTATTTGTTACTGTT, HPV16L reverse primer: CCTCCCCATGTCGTAGGTACTCCTTAAAG and HPV16L TaqMan probe: 6FAM- GTCATTATGTGCTGCCATATCTACTTC -TAMRA) and 4 µl nuclease free water (20 µl total reaction volume) using ABI’s QuantStudio 6 Flex System with default PCR Cycling: Pre Heat activation at 50 °C, 2 min; Initial Denature at 95 °C, 10 min; 40 Cycles of Denaturation at 95 °C, 15 s, Annealing and Elongation at: 60 °C, 1 min. The cycle threshold (CT) values were automatically generated by the software in the system. The samples with CT values ≤ 35 were considered as HPV16 positive.

### Single-cell RNA-sequencing

Fresh carcinoma samples were digested using the Human Tumor Dissociation Kit and the gentleMACS Dissociator (Miltenyi Biotec) per manufacturer recommendations. Cells were washed in 1×PBS, filtered (70 μm), and quantified using acridine orange + propidium iodide (AO/PI) staining on a Cellometer Auto 2000 (Nexcelom). Cells were concentrated to 1000 cells/μL and loaded onto the Chromium Controller (10X Genomics) with a target of 8000 cells per sample. Cells were mixed with barcoded gelbeads and 5′ GEM Kit v2 reagents (10X Genomics), and single-cell capture was performed. Following reverse transcription, cDNA was amplified, and gene expression libraries were constructed according to the manufacturer’s recommendations. Each fragmented DNA library was loaded into a sequencing lane on a NovaSeq or NextSeq system (Illumina) and was sequenced with pair-end reads of 75 bp.

### scRNA-seq data analysis

The resulting fastq files were processed using cellranger multi (-f 10,000; version 7.1.0) with a custom reference combining human reference genome GRCh38 and Ensembl annotation (version 98) with HPV types 6, 11, 16, 18, 33, 35, and 56 genome sequences and annotations obtained from PaVe^52^ and default parameters otherwise. Downstream analysis was performed using the programming language R (version 4.5.2)^53^ and the R package Seurat (version 5.4.0)^54^.

The R package scds (version 1.24.0)^55^ was used to identify potential doublets. More specifically, for all cells, hybrid scores were computed using the function scds::cxds_bcds_hybrid prior to quality control-based filtering, and the 4.8% of cells with the highest hybrid scores were considered potential doublets according to the 10X Genomics User and removed from the analysis. In addition, cells satisfying one or more of the following conditions were removed: cells with (1) ≥25% HBA or HBB UMI counts, (2) ≥25% mitochondrial UMI counts, (3) cells with <250 or >5000 detected transcripts (non-zero UMI counts), and (4) sum of UMI over all genes <500.

Unless stated otherwise, functions were used with default parameters. UMI counts were normalized (Seurat::NormalizeData), variable features were identified (Seurat::FindVariableFeatures), the data was scaled (Seurat::ScaleData(vars.to.regress = c(’nCount_RNA’))), and a PCA was performed using the variable features (Seurat::RunPCA). Harmony (version 1.2.4)^56^ was applied to (Seurat::RunHarmony) to integrate individual data sets at the sample level. The ‘harmony’ reduction was used to generate UMAP embeddings (Seurat::RunUMAP) and performed graph-based clustering. Heatmaps were generated using ComplexHeatmap (version 2.24.1)^57^. Data processing and visualization were performed using tidyverse (version 2.0.0)^58^ and ggplot2 (version 2.4.1)^59^. Box plots represent the following information: the box corresponds to the interquartile range (IQR), horizontal lines inside the box indicate the median, whiskers (vertical bars) extend to the smallest and largest data points within 1.5*IQR from the lower and upper quartiles, respectively, and data points beyond the boundaries of whiskers reflect outliers. Paired statistical tests were performed whenever suitable. Custom reference gene expression profiles were computed using Seurat::AggregateExpression for each cluster.

Ligand-receptor interaction analysis was performed using the P package CellChat (version 2)^60^ following the developer recommendations. The interaction component of the database was updated to include HLA class 2 - LAG3 and HMGB1 – HAVCR2 interaction terms. The analysis was performed using default parameters except for the following function calls: CellChat::computeCommunProb(type = ‘truncatedMean’, trim = 0.1) and CellChat::filterCommunication(min.cells = 10).

### Copy number alteration analysis in scRNA-seq data

scRNA-seq copy number analysis was performed considering the local gene detection frequency. More specifically, scRNA-seq data is characterized by sparsity, i.e., for each cell, only a subset of transcripts is ultimately sequenced. We reasoned that the frequency at which genes within a contiguous chromosomal segment are detected should be related to the total copy number. In this analysis, we computed the local gene detection frequencies (LGDF) within a window of 200 consecutive genes. Expression of each gene was represented as a binary variable, where 0 and 1 correspond to not expressed and expressed, respectively. Furthermore, all annotated genes were considered in this analysis. Center on each gene, the LGDF was computed using the R package caTools (version 1.18.3)^61^ function caTools::runmean(k = 200, endrule = ‘mean’) with default parameters otherwise. Next, for each genomic position, the relationship between LGDF (square root) and the number of total transcripts (log10) detected was assessed within fibroblasts and endothelial cells, used as copy number normal references, using linear models (stats:lm(LGDF ~ transcript count)). Using the resulting position-specific linear models, copy number alteration (CNA) scores for all cells and genomic positions were obtained as residuals comparing both predicted and observed LGDF. Next, for each genomic position resulting CNA scores were centered around zero. For each cell, the median-centered CNA score was subtracted from all CNA scores. Furthermore, for each genomic position, the 0.2 – and 0.8 – quantiles (0.2Q and 0.8Q) CNA scores were computed using only the reference cells. To remove noise, CNA scores >0.8Q were mapped to CNA score – 0.8Q; CNA scores <0.2Q were mapped to CNA score – 0.2Q; remaining values were mapped to zero.

### Spatial gene expression profiling

Xenium In Situ Technology from 10X Genomics was used to spatially detect gene expression using a custom probe set that included HPV16 genes (10X Genomics Custom Probe Set number 8XCY2D; Supplementary Dataset 1). We manually curated a list of 297 genes that satisfied one or more of the following criteria: (1) cell-type-specific genes identified by differential gene expression analysis between different cell-type clusters (Figs. 1b), (2) genes of different functional categories, such as hypoxia or interferon gamma signaling, or (3) HPV genes. Word cloud representation was generated using the R package wordcloud (version 2.6)^62^. Tissue sections of 5 μm thickness from FFPE blocks were placed on Xenium slides. The tissue sections were first deparaffinized and de-crosslinked according to the 10X Genomics Demonstrated Protocol CG000580. Subsequently, probe hybridization, ligation, amplification, and subsequent steps were performed as outlined in the 10X user guide (CG000582). The custom probe set was then used in the hybridization step. A Xenium run was set up as per the Xenium Analyzer user guide (CG000584), running instrument software v1.6.1.0 and onboard analysis software v1.6.0.8 (10X Genomics). Digital images were acquired of the hematoxylin and eosin-stained tissue section used for spatial transcriptomic analysis and annotation of tumor parenchyma and stroma (malignant samples) or non-malignant epithelium and stroma (non-malignant samples) was performed by an anatomic pathologist. The estimated number of false positive transcripts per cell for each sample, computed based on negative control probes/codewords by the manufacturer’s companion analysis software, is as follows: NCI_3: 0.101; NCI_4: 0.159; NCI_14: 0.0316; NCI_22: 0.0412; NCI_6: 0.0878; NCI_8: 0.0492; NCI_9: 0.0559; NCI_10: 0.0488; NCI_12: 0.0781; NCI_15: 0.0672; NCI_21: 0.0862.

### Single-cell spatial gene expression analysis

Xenium data was processed on-instrument using the Xenium Analyzer. Downstream analysis of the processed count data was performed using R and Seurat^54^. To reduce the effects of misassigned transcripts, we considered nuclear transcripts in this analysis. Furthermore, cells with no transcripts were removed from the analysis. Nearest neighbors were identified using Seurat::FindNeighbors, considering Euclidean distances. Cell-type classification was performed using SingleR^63^ with custom reference gene expression profiles based on scRNA-seq data as outlined above. Stromal and parenchymal cells with a minimum transcript count of 20 that were classified as myeloid were used for a more resolved myeloid subset analysis. Similarly, stromal and parenchymal T/NK cells with a minimum transcript count of 40 were separately analyzed for subset classification. Stromal and parenchymal myeloid and T/NK cells were analyzed analogous to the scRNA-seq data with Seurat::RunPCA(npcs = 15) and Seurat::RunHarmony(theta = 0.5). Of note, the NK cell cluster may contain NKT cells, which display transcriptional similarities^64^.

Cells with at least two detectable transcripts of MKI67, TOP2A, or GINS2 were considered to be proliferative. Differential gene expression analysis was performed using Seurat::FindAllMarkers, considering adjusted *p*-values. To identify common chemokine foci-specific genes using HPV-OPSCC samples, differential gene expression was performed comparing epithelial cells associated with CXCL9/10/11, CXCL8, and remaining parenchyma using stringent filtering conditions; only genes identified as differentially expressed, using an adjusted *p*-value cutoff of 10^−100^, in at least three comparisons were considered. Gene sets related to apoptosis, glycolysis, hypoxia, or IFN-γ were obtained from the Reactome data base^65^. The final gene expression signatures were defined as the intersection between genes represented in our probe set and the corresponding Reactome gene sets. The signature expression was quantified as follows: the mean gene counts over all genes belonging to the signature were computed; one was added to the resulting mean, and the number was log2-transformed. Of note, PSMB8 and PSMB9, which are IFN-γ inducible, were removed from the hypoxia signature to avoid confounded results. Gene set enrichment analysis within differentially expressed genes was performed using clusterProfiler::compareCluster()^66^ using Reactome terms and considering HPV status and chemokine compartment as covariates. Differential gene expression analysis comparing HPVon and HPVoff cells was performed for samples and chemokine compartments separately using Seurat::FindMarkers(min.pct = 0.2); only genes with an adjusted *p*-value ≤ 10^−10^ were considered as differentially expressed. Genes identified across multiple comparisons, e.g., within different samples, were not further summarized to retain quantitative information. Mutual exclusivity between *CXCL8* and *CXCL9/10/11* foci was evaluated considering the number of parenchymal cells assigned to *CXCL8* foci only, *CXCL9/10/11* foci only, *CXCL8* and *CXCL9/10/11* foci, and neither *CXCL8* nor *CXCL9/10/11* foci. Assuming independence, the probability of a cell belonging to both *CXCL8* and *CXCL9/10/11* foci was obtained as the product of the two marginal probabilities to reflect expected events and compared to the observed probability estimated based on the observed event counts. The resulting probabilities were compared to obtain the logodds = log(p_observed/p_expected). Corresponding *p*-values were obtained considering the complete event-count contingency table and a Fisher’s exact test.

### Visium data analysis

Published Visium data was obtained from the Gene Expression Omnibus using the following accession numbers: GSE252265^67^, GSE181300^68^, and GSE208253^69^. The data was processed analogously to the scRNA-seq data. Parenchymal visium spots were identified based on the average expression of all available keratin genes, i.e., for each sample, the distribution of average keratin expression was computed and used to determine an expression cutoff such that all spots expressing greater than or equal keratin levels were classified as parenchymal. Gene signature expression was computed using Seurat::AddModuleScore.

### Survival analysis

Survival analysis was performed using clinically annotated TCGA HPV-HNSCC samples^70^ using the R packages survival (version 3.8-3)^71^ and survminer (version 0.5.2)^72^. The following TPM expression values were used for sample stratification: CXCL2: 4; CXCL8: 49; CXCL9: 30; CXCL10: 138; CXCL11: 7.

#### Multispectral immunofluorescence

Formalin-fixed paraffin-embedded tumors were sectioned at 5 µm, baked at 60 °C for 30 min, soaked in Bond Dewax Solution (Leica), and rehydrated. Deparaffinization and staining of all slides were performed on the Leica BOND RX Autostainer (Leica). Before being used in combination, the specificity and optimal dilution of each antibody were individually determined with chromogenic immunohistochemistry (3’−3’ diaminobenzidine tetrahydrochloride hydrate; DAB) using slides from normal tonsil and head and neck carcinoma, consistent with best-practice guidelines^73^. Heat-induced epitope retrieval was performed by heating to 95 °C in BOND epitope retrieval solutions ER1 or ER2 (Leica). Tyramine Signal Amplification (TSA) Opal technology was used for immunofluorescence staining. After individual primary antibody optimization, primary and secondary antibody and opal pairings were optimized for minimum background and desired signal amplification in monoplex immunofluorescence using head and neck carcinoma sections. Antibody and amplification reagents are listed in Table 1. Slides were coverslipped using the Leica CV5030 automated coverslipper after staining. Whole slide images were obtained at 40× magnification using 5-color whole slide unmixing filters on a Vectra Polaris. All samples were stained and scanned concurrently.

**Table 1:** Antibodies used for immunofluorescence

Antibody	Vendor/Clone	Catalog #	Dilution 1:	HIER	Secondary antibody	Opal	Opal dilution 1:
Tim3	CST[D5D5R]	ab245620	25	Leica ER2(AR9640) / 20 min	BioCare MACH 2 Rabbit HRP-Polymer (RHRP520)	520	150
PD-1	Abcam [EPR4877(2)]	ab137132	750	Leica ER2(AR9640) / 20 min	Akoya OPAL POLYMER HRP MS + RB, 1×, (ARH1001EA)	570	150
CD8	Abcam [EPR10640-2]	ab215041	2000	Leica ER2(AR9640) / 20 min	Akoya OPAL POLYMER HRP MS + RB, 1×, (ARH1001EA)	480	150
PanCK	Santa Cruz [AE1/AE3]	sc-81714	400	Leica ER2(AR9640) / 20 min	Akoya OPAL POLYMER HRP MS + RB, 1×, (ARH1001EA)	780	50

#### Immunofluorescence analysis

Whole slide analysis of each stained slide was performed with HALO Image Analysis software (v3.3, Indica Labs). Whole slide annotations were performed using the Random Forest Tissue Classifier Algorithm. Standard nuclear segmentation was used. The HALO AI Nuclear Segmentation Classifier was trained to identify nuclei of various sizes and used for the CD8-positive cell detection. Fluorescence intensities of each marker for each cell were determined using the HALO Highplex FL Analysis Algorithm. Separate Highplex FL Analysis Algorithms were used for tumor and immune cells, given differences in nuclear size. Fluorescence thresholds used to assign positivity for a given marker were determined for each sample. Cell density was defined as the absolute number of positive cells per unit area (mm^2^).

### HPV-related oropharyngeal carcinoma cell lines and treatments

HPV16-positive HNSCC cell lines UM-SCC-47 and UM-SCC-104 cells, and the HPV-negative cell lines UM-SCC-1 and UM-SCC-9 were obtained from the University of Michigan and UPCI:SCC152 cells were obtained from the University of Pittsburgh under material transfer agreements with the NIH. Cells were cultured in Dulbecco’s Modified Eagle Medium, 10% fetal bovine serum, 1% pen-strep antibiotics, and 1% L-glutamine (defined as complete media, CM) under sterile conditions at 37 °C/5% CO_2_, used at low passage number, and serially tested to ensure mycoplasma negativity. At 70–80% confluency, cells were exposed to hypoxia (0% O_2_), recombinant human IFNγ (20 ng/mL, PeproTech), or recombinant human IL-1β (1 ng/mL), alone or in combination, for variable time courses as indicated in each figure legend. Hypoxia (0% O_2_) was created by incubating neutrophilic cells in a sealed modular incubator hypoxia chamber (Billups-Rothenberg) with an O_2_ sensor and flushed with 0% O_2_ for 10 min. Cells within the hypoxia chamber were incubated in the same incubator as the normoxic cells. Treated or control tumor cells were then used for flow cytometry, RT-PCR, or T cell cytotoxicity experiments. Supernatants from control or treated cells were used for ELISAs.

### Flow cytometry for cell-surface markers

Cells were harvested at <90% confluence and resuspended at 1 × 10^6^ cells/mL in 1×PBS with 0.5% BSA. Primary fluorophore-conjugated anti-human pan-HLA class I (clone W6/32, Biolegend) and HLA-DR (clone L243, Biolegend) antibodies were added at the manufacturer-recommended concentrations and incubated for 30 min on ice in the presence of Human TrueStain FcX. Cells were washed and passed through a 40 μm filter. Data was acquired on a BD LSRFortessa. Doublets and dead cells were excluded from analysis by forward light-scatter and using the viability dye Sytox Blue (Thermo). Data were analyzed using FlowJo software (TreeStar).

### Cell proliferation assays

Cells were plated at variable densities in 10 cm cell culture plates in CM and exposed to control (volume equivalent 1×PBS) or IFNγ for up to 10 days. Plates for control and IFNγ exposure were plated at equal cell density for each timepoint. Cell passage was not required for cells cultured for up to 7 days. For cells cultured for 10 days, one passage was required. Viable cells were counted after harvest, and the relative live cell number of IFNγ-exposed cells compared to control was calculated as: (number of viable IFNγ-exposed cells/number of viable control cells).

### Flow cytometry-based cell-cycle analysis

Cells were exposed to control (volume equivalent 1×PBS) or IFNγ in CM for up to 7 days. The day before each analysis, cells were harvested, and 6 × 10^5^ cells were plated into each well of a 6-well culture plate and cultured for 24 h in control or IFNγ conditions. EdU reagent from the Click-iT EdU Kit (Thermo) was added for 2 h prior to cell harvest. Cells were harvested via scraping and stained with a primary fluorophore-conjugated anti-human pan-HLA class I (clone W6/32, Biolegend) for 30 min on ice. Cells were stained with a fixable viability marker (Zombie Aqua, Biolegend), then fixed and permeabilized with the FoxP3 Transcription Factor Staining Buffer Set (Thermo) per manufacturer recommendations. The remaining steps of the Click-iT EdU Kit were performed per the manufacturer's recommendations. FxCycle Violet (Thermo) was added per manufacturer recommendations prior to data acquisition on a BD LSRFortessa.

### ELISA

ELISA kits for CXCL8, CSF3, CXCL10, and CSF1 were purchased from R&D Systems and used per manufacturer recommendations. For all experiments, plotted values represent concentrations normalized to production by 1 × 10^5^ viable cells counted at the time of supernatant collection.

### HPV16 E6 and E7 quantitative RT-PCR

Cells were harvested, washed in 1×PBS, and lysed with TRIzol (Thermo). RNA was isolated using Qiagen RNeasy Mini or Micro Kits as per the manufacturer's recommendations. An experiment standardized quantity of RNA was converted to cDNA using the High-Capacity cDNA Reverse Transcription Kit from Applied Biosystems per manufacturer recommendations at 25 °C for 10 min, 37 °C for 2 h, and 85 °C for 5 min. Thirty nanograms of cDNA from each experimental sample were loaded in technical triplicate onto a MicrAmp® Fast Optical 96-Well Reaction Plate (Applied Biosystems). Relative expression (ΔΔCT^2^) of *HPV16E7* compared to a housekeeping gene (*Gapdh*) was assessed by adding Taqman Universal PCR Master Mix (Applied Biosystem) along with each primer in a total reaction volume of 20 µL in each well. The qPCR reaction was completed using the QuantStudio 6 Flex analyzer (Applied Biosystems). The primer for human *Gapdh* was obtained from Integrated DNA Technologies (Assay ID: Hs.PT.39a.22214836). The primer for *HPV16E7* was custom-ordered and obtained through Integrated DNA Technologies with the following primer and probe sequences: Forward primer: TCA GAG GAG GAG GAT GAA ATA GA; Reverse primer: GCA CAA CCG AAG CGT AGA; Probe: 56-FAM/AGA ACC GGA /ZEN/CAG AGC CCA TTA CAA /3IABkFQ.

### β-galactosidase assays

The CellEvent Senescence Green Flow Cytometry Assay Kit was purchased from Thermo Fisher and used per manufacturer recommendations.

### Neutrophil isolation from tumors

Fresh carcinoma samples were digested using the Human Tumor Dissociation Kit and the gentleMACS Dissociator (Miltenyi Biotec) per manufacturer recommendations. Cells were washed in 1×PBS, filtered (70 μm), and RBC lysis was performed using the RBC lysis buffer from Biolegend per manufacturer recommendations. Leukocytes were isolated from the digested tumor using an 80/40% Percoll centrifugation gradient. Dead cell removal was then performed using the EasySep Dead Cell Removal Kit from StemCell Technologies per manufacturer recommendations. Neutrophilic cell positive magnetic isolation was then performed using the EasySep Human CD15 Positive Selection Kit from StemCell Technologies per manufacturer recommendations. Neutrophilic cells were suspended in RPMI1640 media supplemented with 10% FBS, 2 μmol/L β-ME, HEPES, nonessential amino acids, glutamine, and antibiotics. (T cell media) All steps were performed at room temperature. Isolated neutrophilic cells were then used in T cell suppression assays.

### Peripheral blood neutrophil isolation and culture

Neutrophilic cells were isolated from fresh whole blood using the EasySep Direct Human Neutrophil Isolation kit per manufacturer recommendations at room temperature. Fresh neutrophilic cells were suspended in T cell media at 5 × 10^5^ cells/mL and exposed to hypoxia (0% O_2_), recombinant human CXCL8 (1 ng/mL, PeproTech) or recombinant human CSF3 (G-CSF, 50 ng/mL, PeproTech), alone or in combination, for 24 h at 37 °C/5%CO_2_. Cultured neutrophilic cells were then used in T cell suppression assays.

### T cell suppression assays

CD8^+^ and CD4^+^ T cells were isolated from PBMC from a common healthy donor via negative magnetic selection using the EasySep Human T Cell Enrichment Kit from Stemcell Technologies as per the manufacturer's recommendations. T cells were then stained with either CFSE (Selleckchem, 5 mM for 3 min) or CellTrace Yellow from Thermo Fisher Scientific per manufacturer recommendations. Stained T cells were stimulated with plate-bound anti-human CD3 (OKT3) and CD28 (CD28.2) and co-cultured with whole PBMC, isolated carcinoma-infiltrating neutrophilic cells, or in vitro cultured peripheral blood neutrophilic cells at the indicated PMN-to-T cell ratios for 72 h. Cultured T cells were then stained with anti-human CD8 and CD4 antibodies as well as sytox blue to exclude dead cells, and flow cytometry (BD LSRFortessa) was used to quantify CFSE or CellTrace Yellow dilution. Proliferation was quantified as the percentage of proliferated T cells using FlowJo software.

### HPV-specific cytotoxicity assays

UM-SCC-104 and UPCI:SCC152 cells are both HPV16 and HLA-A*02 positive^24^. 2 × 10^4^ cells were plated in each well of RTCA microplates in CM. For hypoxia-related experiments, initial impedances were registered on the xCELLigence RTCA Reader (Agilent). Plated cells were then removed from the RTCA Reader and exposed to normoxia or hypoxia (0% O_2_) for 24 h at 37 °C/5% CO_2_. Impedance was again registered after placing the plated cells back onto the RTCA Reader. For IFNγ-related experiments, cells were exposed to IFNγ or volume equivalent 1×PBS in CM for 7 days and plated in RTCA microplates for overnight adherence in IFNγ or volume equivalent 1×PBS prior to the addition of effector T cells. Healthy donor peripheral T cells engineered to express an HPV16E7-specific, HLA-A*02-restricted T cell receptor^74^ were used as effector T cells and added at different effector-to-target cell ratios, and real-time impedance measurements in normoxic conditions were used to measure antigen-specific T cell killing of the cancer cells exposed to normoxic or hypoxic conditions. Triton was added to some wells to visualize immediate and complete tumor-cell killing. To compare the kinetics of T cell killing of tumor cells exposed to different conditions, all impedance curves were normalized to 1, corresponding to the time of the addition of the T cells. Percentage loss of target cell index represents percentage T cell killing and was quantified as: ((normalized control cell index-experimental normalized cell index)/normalized control cell index) × 100.

### Statistics

All in vitro experiments were performed in at least three biological replicates, and data points shown represent the individual result for the biological experiment or the mean of at least three technical replicates for each biological replicate. The number of biological replicates is indicated in each figure legend. Analyses for functional experiments were performed with GraphPad Prism v10.3. A *t*-test was used to determine the significance of differences between two groups. For experiments with more than two groups, a one-way ANOVA with Tukey’s multiple comparison test was used. Two-sided statistical tests were performed to evaluate statistical significance when using T and Wilcoxon tests.

### Reporting summary

Further information on research design is available in the Nature Portfolio Reporting Summary linked to this article.

## Supplementary information

Supplementary Information

Description of Additional Supplementary Files

Supplementary Dataset 1

Reporting Summary

Transparent Peer Review file

## Source data

Source data

## Data Availability

Raw and processed data used in this study is available through Gene Expression Omnibus using accession numbers GSE290040 (scRNA-seq) and GSE290041 (Xenium). Source data are provided with this paper.

## Code availability

Custom scripts used for the analysis were developed using publicly available R packages and will be made available upon reasonable request.
