# Supplementary material for: Spatial cellular order underlies locally-confined mechanisms of immune resistance in oropharyngeal cancer
Source: Nat Commun. 2026 Jun 13;17:7512. doi: 10.1038/s41467-026-74318-z (PMC13408450; doi:10.1038/s41467-026-74318-z)
Supplement: Supplementary file 1 — Supplementary Information [file 41467_2026_74318_MOESM1_ESM.pdf]

Supplementary Figures and Tables:

Spatial cellular order underlies locally-confined mechanisms of immune resistance in oropharyngeal cancer

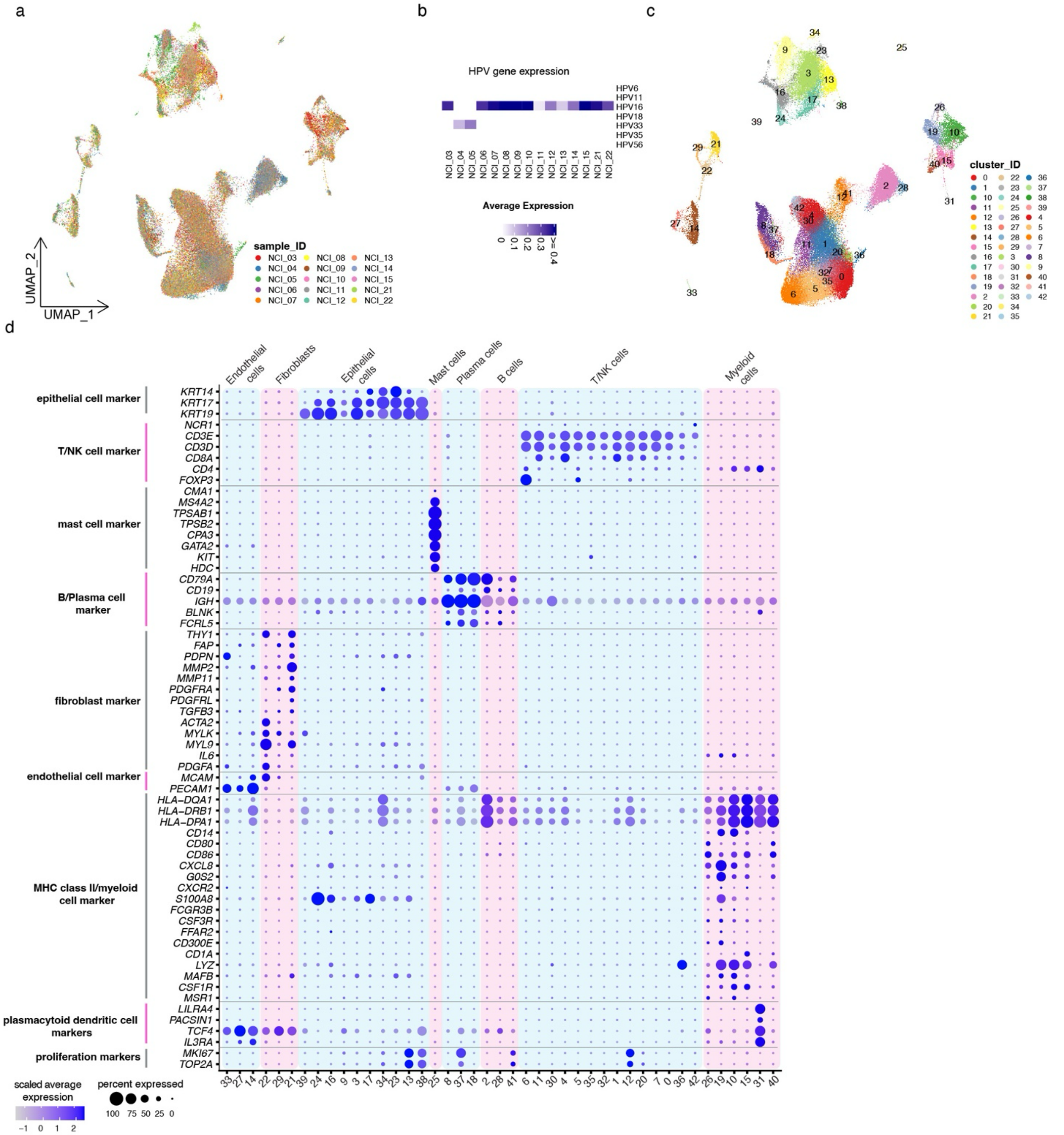

Supplementary Figure 1. scRNA-seq cell-type classification.

a, Scatter plot showing uniform manifold approximation and projection (UMAP) embedding of single cells resulting HPV-OPSCC scRNA-seq colored by sample identity.

b, Heatmap showing average expression of HPV genes associated with different HPV types computed based on the scRNA-seq data for each sample.

c, Scatter plot showing UMAP embedding of single cells resulting HPV-OPSCC scRNA-seq colored by cluster.

d, Dot plot showing expression of select cell type/state marker genes for all cell clusters shown in (a). For each gene, circle color and size correspond to scaled average expression and fraction of cells with non-zero expression, respectively.

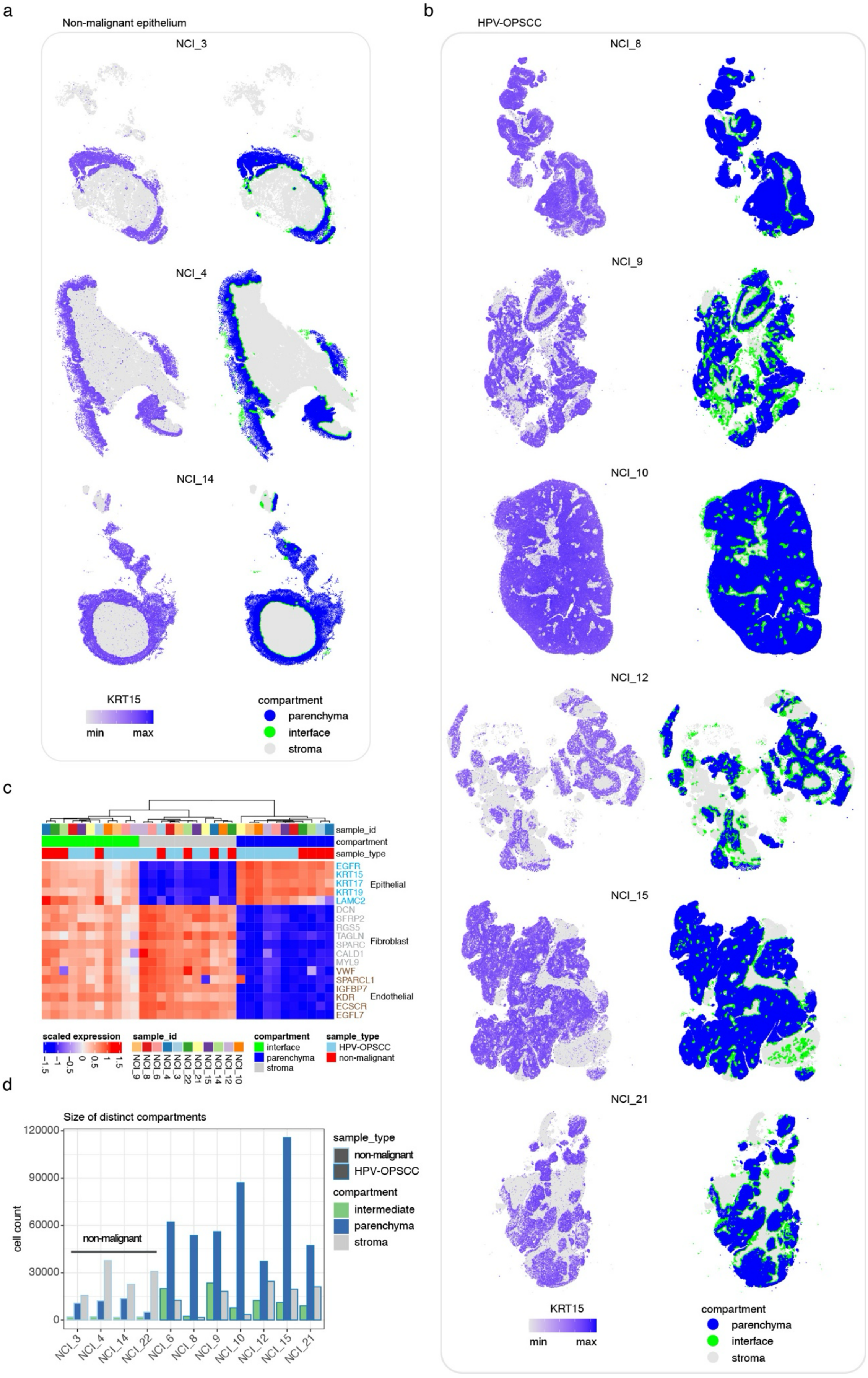
a
Non-malignant epithelium
NCI_3
NCI_4
NCI_14
KRT15
min
max
compartment
parenchyma
interface
stroma
b
HPV-OPSCC
NCI_8
NCI_9
NCI_10
NCI_12
NCI_15
NCI_21
KRT15
min
max
compartment
parenchyma
interface
stroma
c
sample_id
compartment
sample_type
EGFR
KRT15
KRT17
KRT19
LAMC2
Epithelial
DCN
SFRP2
RGS5
TAGLN
SPARC
CALD1
MYL9
Fibroblast
VWF
SPARCL1
IGFBP7
KDR
ECSCR
EGFL7
Endothelial
scaled expression
−1.5
−1
−0.5
0
0.5
1
1.5
sample_id
NCI_9
NCI_8
NCI_6
NCI_4
NCI_3
NCI_22
NCI_21
NCI_15
NCI_14
NCI_12
NCI_10
compartment
interface
parenchyma
stroma
sample_type
HPV-OPSCC
non-malignant
d
Size of distinct compartments
cell count
120000
90000
60000
30000
0
non-malignant
NCI_3
NCI_4
NCI_14
NCI_22
NCI_6
NCI_8
NCI_9
NCI_10
NCI_12
NCI_15
NCI_21
sample_type
non-malignant
HPV-OPSCC
compartment
intermediate
parenchyma
stroma

Supplementary Figure 2. Classification of tumor parenchymal and stromal compartments.

a, b, Scatter plots showing cells within non-malignant epithelial (a) and HPV-OPSCC tissues (b). Colors correspond to KRT15 expression (a, b; left) or compartment classification (a, b; right).

c, Heatmap showing expression of epithelial and stromal marker genes within aggregate gene expression profiles of cells assigned to the epithelial compartment, stromal compartment or interface for each sample. Color corresponds to scaled average expression.

d, Bar graph showing the number of cells for each sample classified as stromal, intermediate and parenchymal.

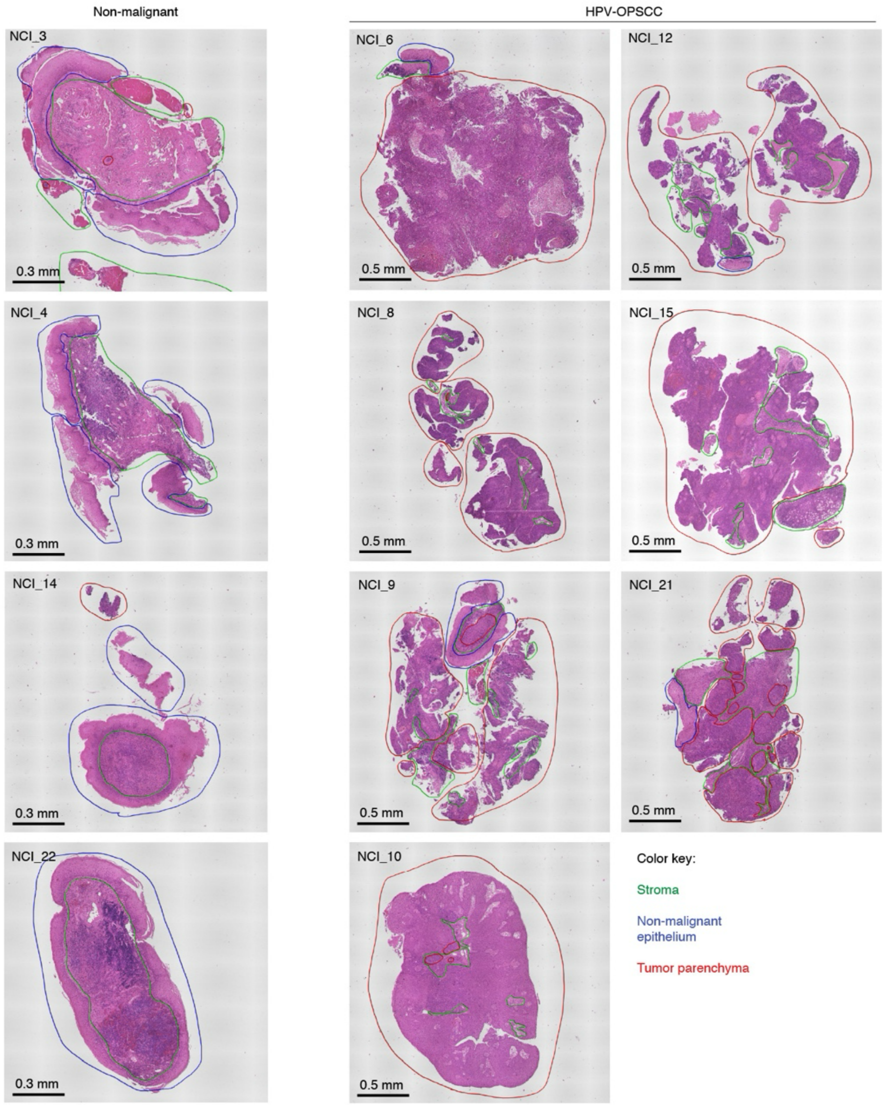

Non-malignant
HPV-OPSCC
NCI_3
0.3 mm
NCI_6
0.5 mm
NCI_12
0.5 mm
NCI_4
0.3 mm
NCI_8
0.5 mm
NCI_15
0.5 mm
NCI_14
0.3 mm
NCI_9
0.5 mm
NCI_21
0.5 mm
NCI_22
0.3 mm
NCI_10
0.5 mm
Color key:
Stroma
Non-malignant epithelium
Tumor parenchyma

Supplementary Figure 3. Histology of tumor and non-malignant epithelium.

Histologic annotation by a pathologist of epithelial/parenchymal and stromal compartments using H&E staining of the same tissue section used for SGE profiling of non-malignant epithelial (left column) and HPV-OPSCC samples used for SGE analysis (center and right column).

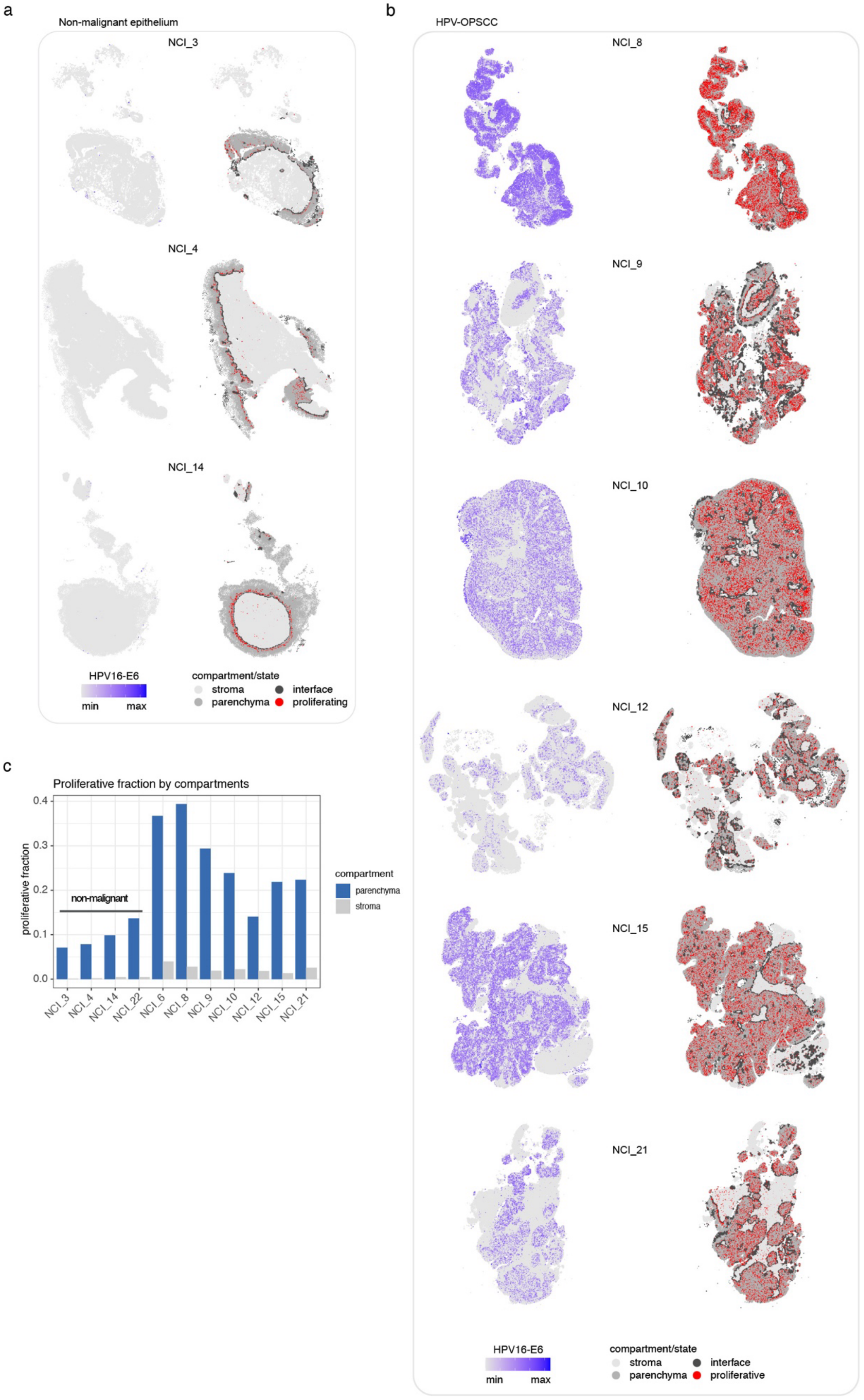
a
Non-malignant epithelium
NCI_3
NCI_4
NCI_14
HPV16-E6
min
max
compartment/state
stroma
interface
parenchyma
proliferating
b
HPV-OPSCC
NCI_8
NCI_9
NCI_10
NCI_12
NCI_15
NCI_21
HPV16-E6
min
max
compartment/state
stroma
interface
parenchyma
proliferative
c
Proliferative fraction by compartments
proliferative fraction
0.0
0.1
0.2
0.3
0.4
non-malignant
NCI_3
NCI_4
NCI_14
NCI_22
NCI_6
NCI_8
NCI_9
NCI_10
NCI_12
NCI_15
NCI_21
compartment
parenchyma
stroma

Supplementary Figure 4. Expression of HPV genes and proliferation markers.

a, b, Scatter plots showing cells within non-malignant epithelial (a) and HPV-OPSCC tissues (b). Colors correspond to HPV16-E6 expression (a, b; left) or compartment classification with additional indication of proliferative cells (a, b; right).

c, Bar graph showing the proportion of stromal and parenchymal cells classified as proliferative.

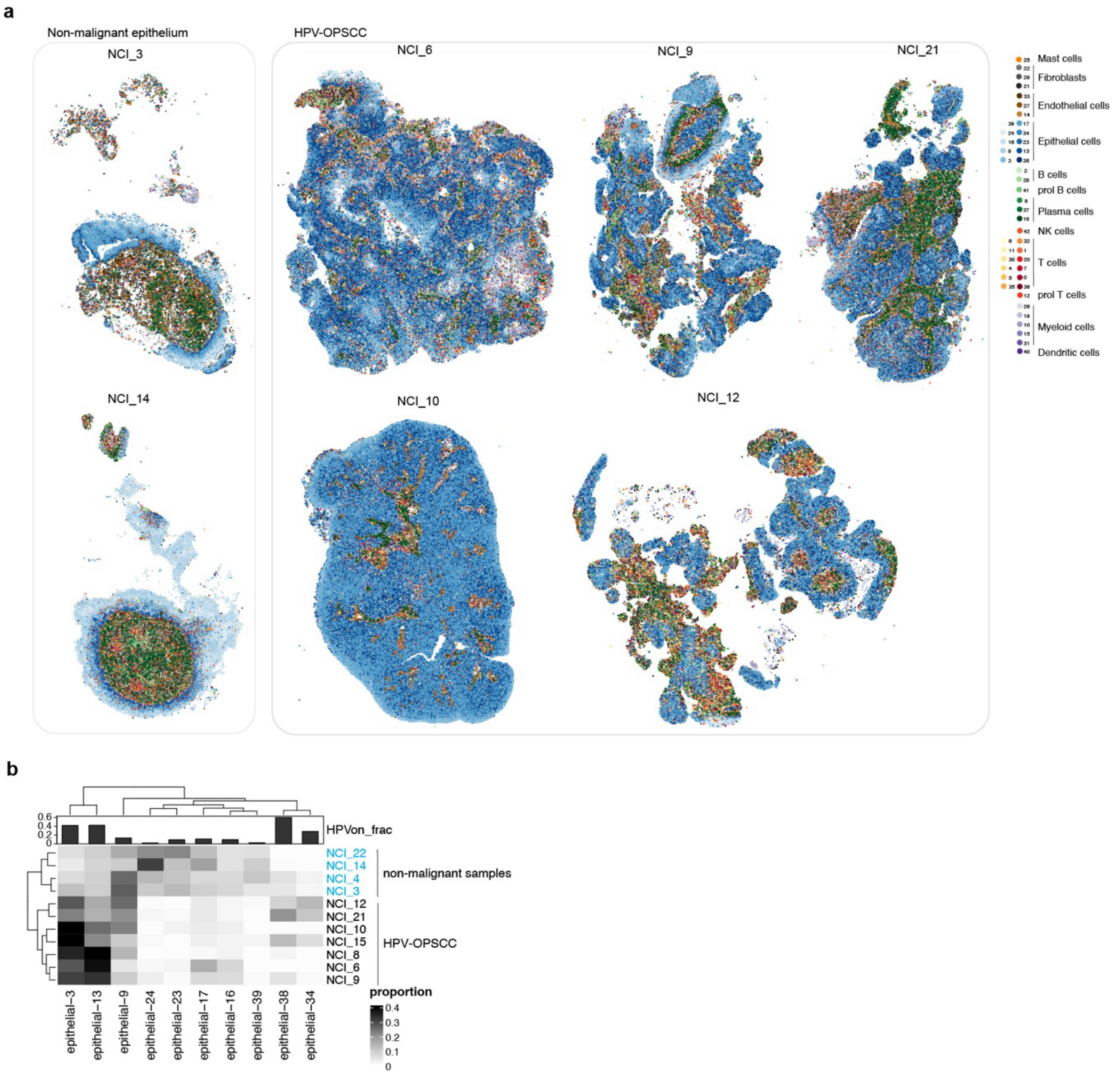

Supplementary Figure 5. Cell type classification within spatial data.

a, Scatter plots showing cells within non-malignant epithelial (left) and HPV-OPSCC tissues (right). Colors reflect scRNA-seq-based cell type classification.

b, Heatmap showing the proportion of cells within each SGE data set classified as indicated epithelial cell type using scRNA-seq-based cell type classification. Bar graph shows the fraction of cells within each scRNA-seq cluster expressing detectable levels of HPV genes.

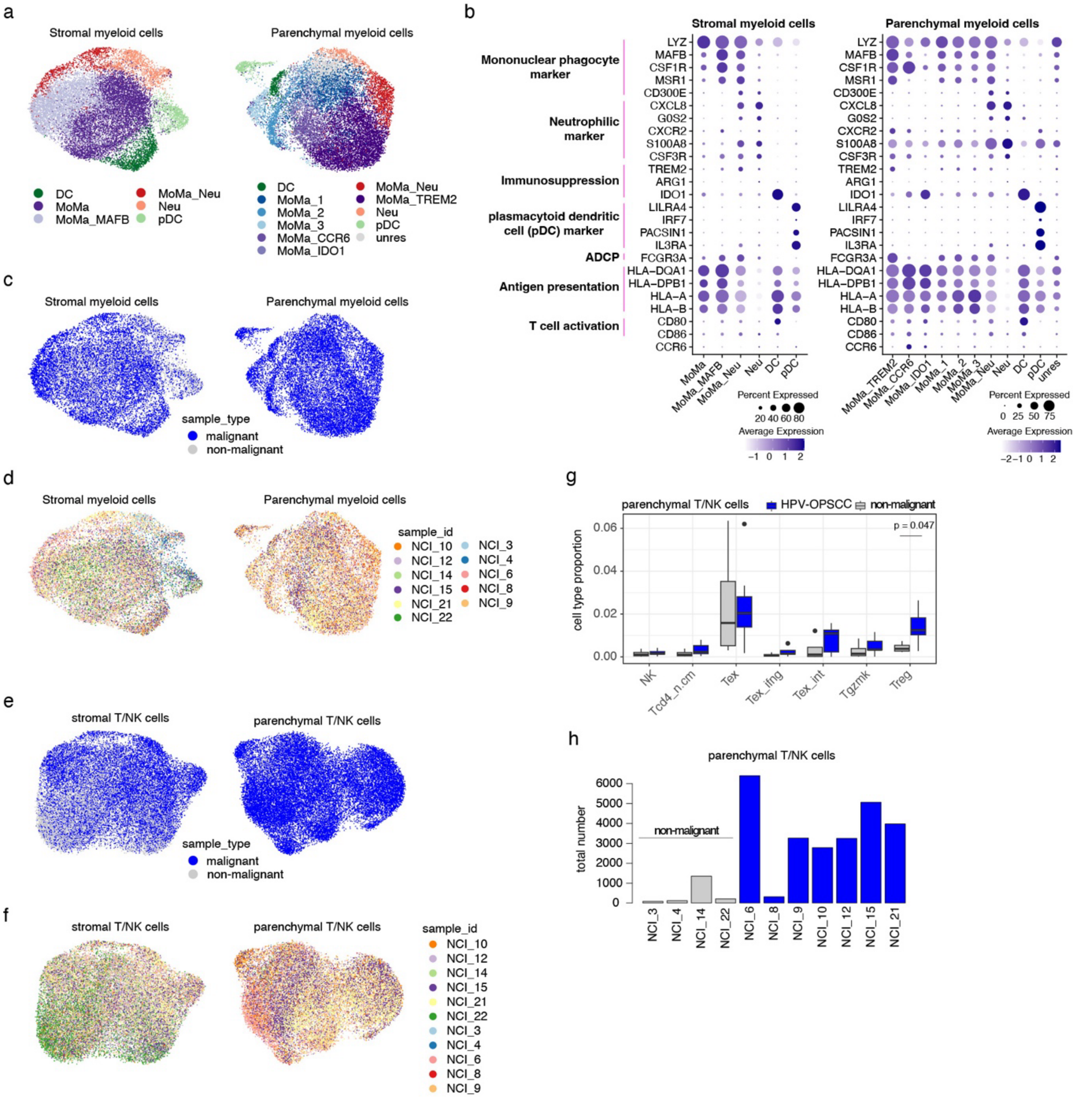

Supplementary Figure 6. Myeloid and T cells within stromal and parenchymal compartments in spatial data.

a, Scatter plots showing UMAP embedding of stromal (left) and parenchymal (right) myeloid cells identified within single-cell spatial gene expression data colored by cluster identity.

b, Dot plots showing expression of select marker genes associated with myeloid cell identity and function for stromal (left) and parenchymal myeloid cell clusters shown in (a). For each gene, circle color and size correspond to scaled average expression and fraction of cells with non-zero expression, respectively. ADCP = antibody-dependent cellular phagocytosis.

c, d, Scatter plots showing UMAP embedding of stromal (left) and parenchymal (right) myloid cells identified within single-cell spatial gene expression data colored by sample type (c) or identity (d).

e, f, Scatter plots showing UMAP embedding of stromal (left) and parenchymal (right) T/NK cells identified within single-cell spatial gene expression data colored by sample type (e) or identity (f).

g, Box plot showing the parenchymal proportions of indicated T/NK cell subsets within HPV-OPSCC (n = 7) and non-malignant epithelial (n = 4) samples. The box corresponds to the interquartile range (IQR), horizontal line inside the box indicates the median, whiskers (vertical bars) extend to the smallest and largest data points within 1.5*IQR from the lower and upper quartiles, respectively, and data points beyond boundaries of whiskers reflect outliers. P-values are based on paired, two-sided Wilcoxon rank-sum test; *: p <= 0.05. Source data are provided as a Source Data file.

h, Bar graph showing the total number of parenchymal T/NK cells identified within each sample.

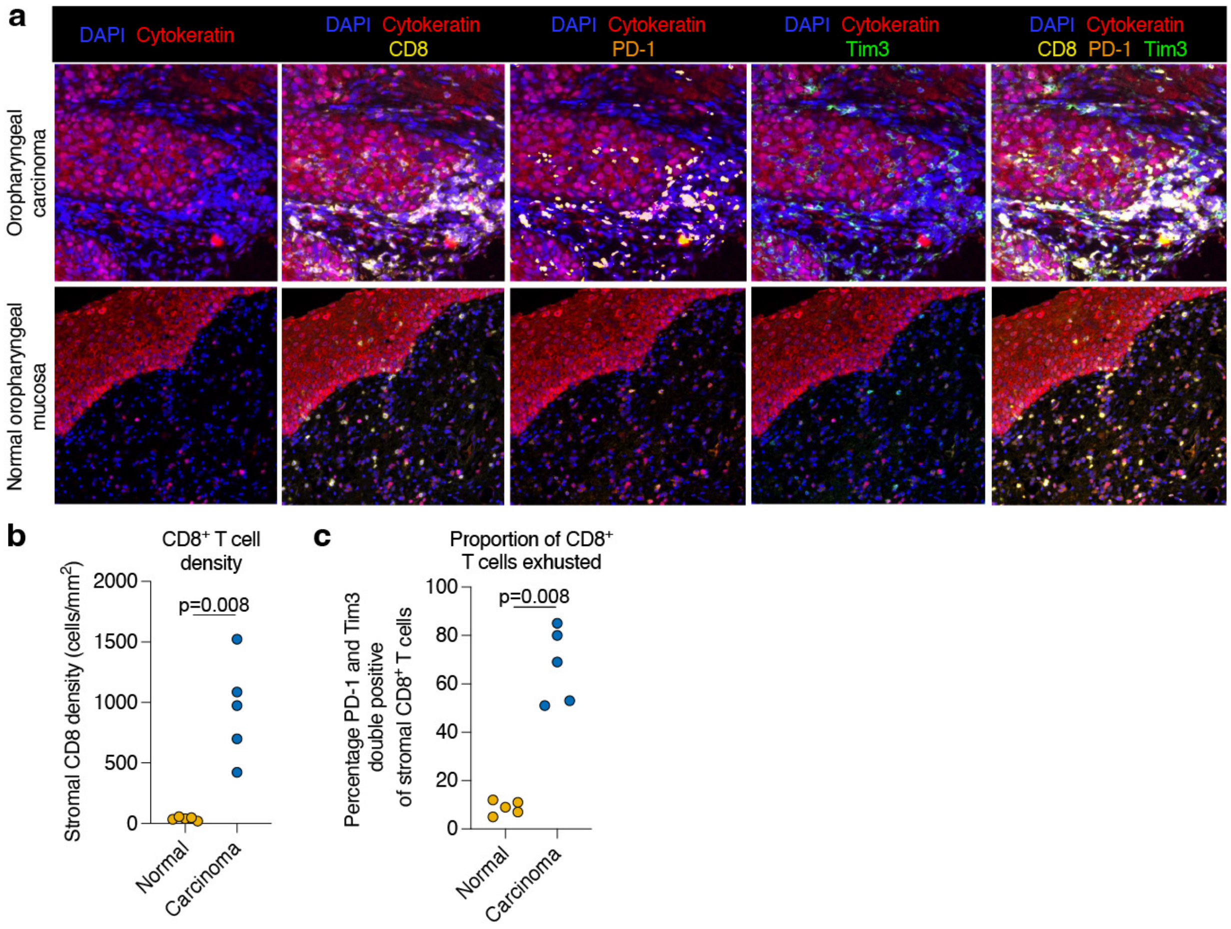

Supplementary Figure 7. Immunofluorescence of non-malignant tonsillar epithelium and HPV-OPSCC.

a, Representative photomicrographs showing immunofluorescence staining for T cell and exhaustion markers in oropharyngeal carcinoma and mucosa clinical specimens.

b, Dot plot showing whole slide quantification of stromal CD8 positive cell density in oropharyngeal carcinoma and mucosa clinical specimens.

c, Dot plot showing whole slide quantification of the percentage of stromal CD8 positive cells that are double positive for PD-1 and Tim3 in oropharyngeal carcinoma and mucosa clinical specimens.

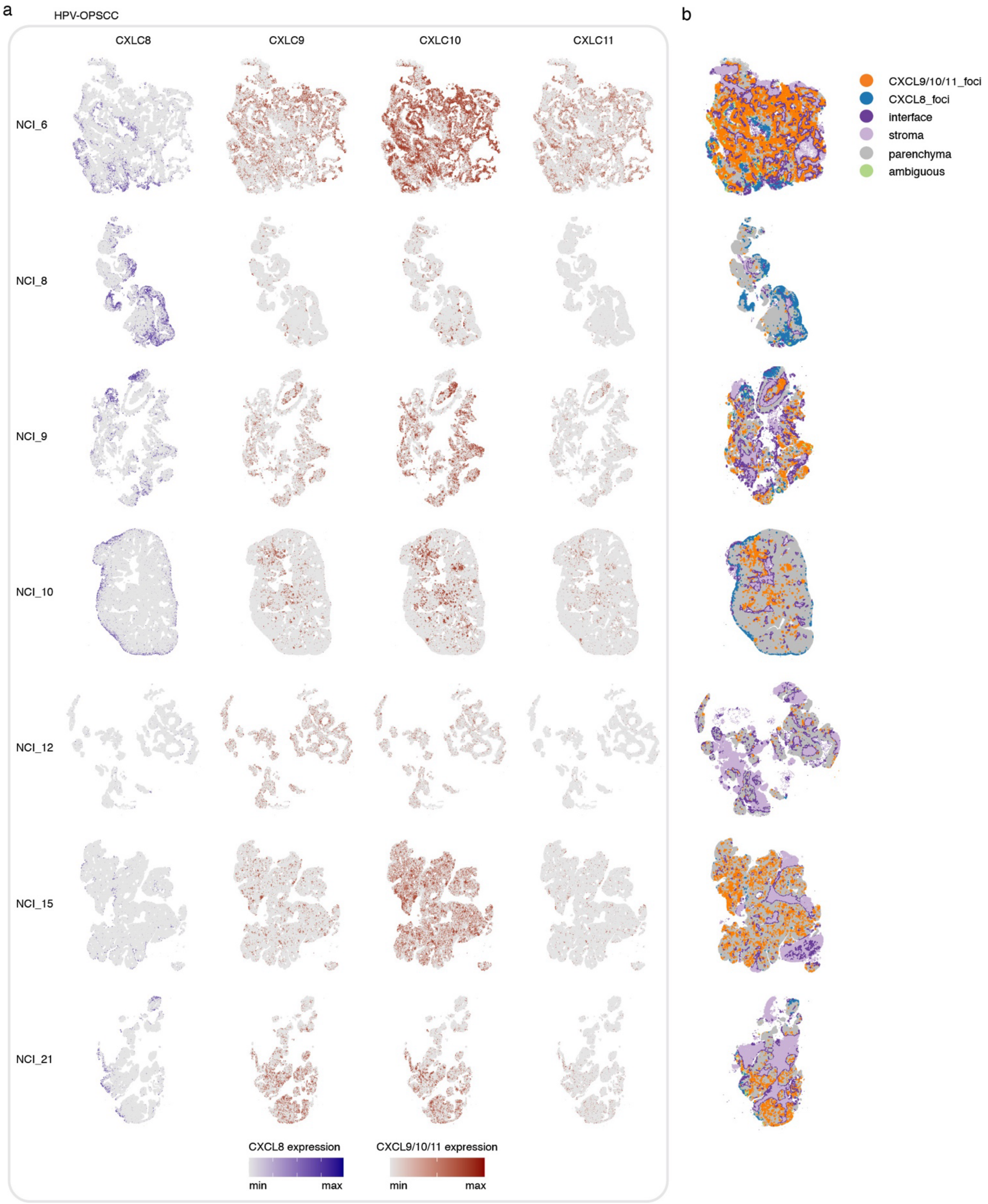
a
HPV-OPSCC
CXLC8
CXLC9
CXLC10
CXLC11
NCI_6
NCI_8
NCI_9
NCI_10
NCI_12
NCI_15
NCI_21
CXCL8 expression
min
max
CXCL9/10/11 expression
min
max
b
CXCL9/10/11_foci
CXCL8_foci
interface
stroma
parenchyma
ambiguous

Supplementary Figure 8. Focal expression of lymphocytic and myeloid cell attracting chemokines in HPV-OPSCC tumor parenchyma.

a, Scatter plots showing cells within HPV-OPSCC tissues. Colors reflect expression of *CXCL8*, *CXCL9*, *CXCL10* or *CXCL11*.

b, Scatter plots showing cells within HPV-OPSCC tissues. Colors correspond to compartments and chemokine foci. Cells assigned to both CXCL9/10/11 and CXCL8 foci are considered ambiguous.

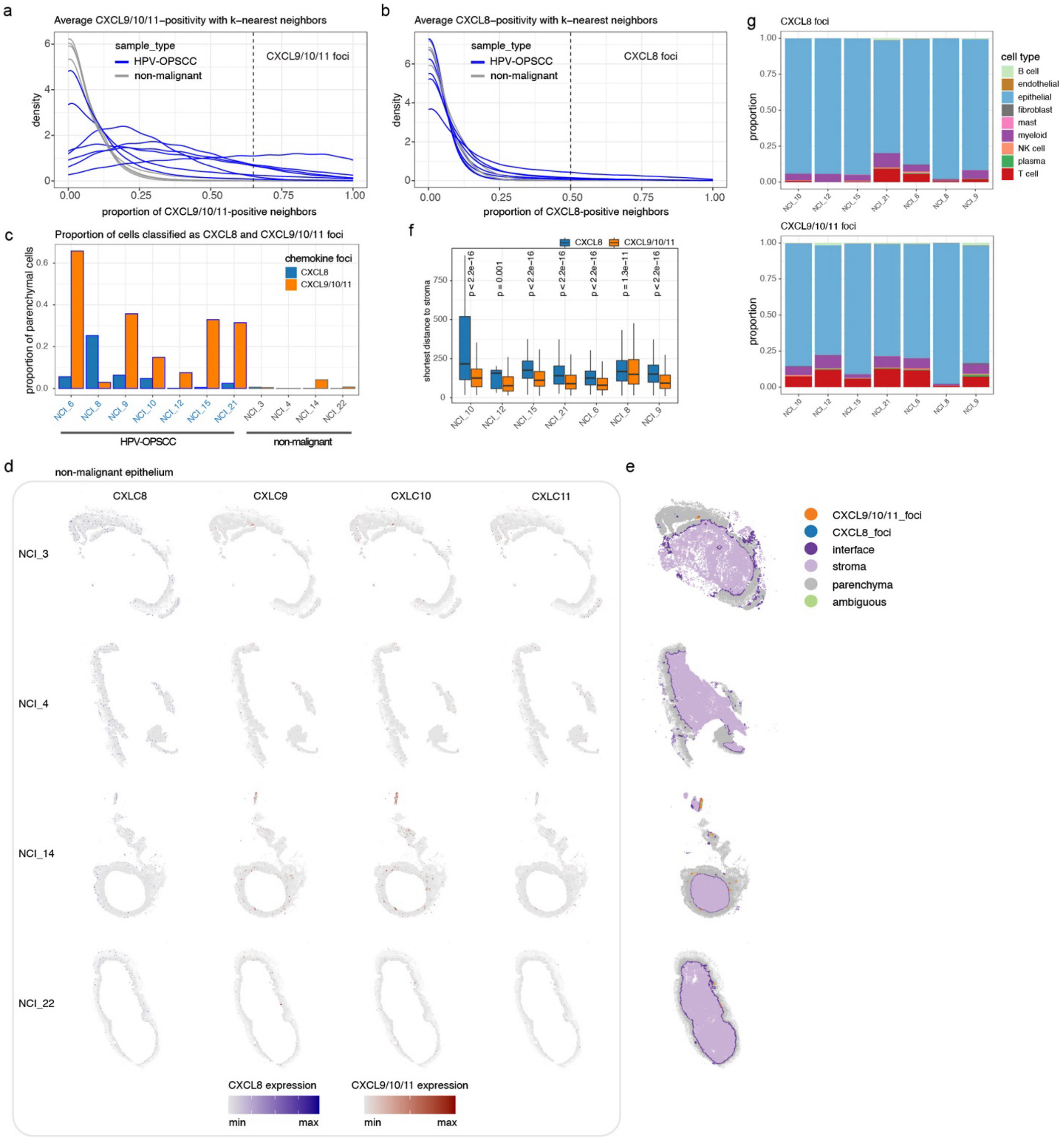

Supplementary Figure 9. Characterization of parenchymal chemokine foci in HPV-OPSCC.

a, b, Line graph showing the probability density of *CXCL9/10/11*- (a) or *CXCL8* – positivity (b). The *CXCL9/19/11*- and *CXCL8* - positivity reflects the fraction of neighboring cells with at least one transcript of *CXCL9*, *CXCL10* or *CXCL11* (a) or *CXCL8* (b), respectively. For each

parenchymal cell the ten nearest neighbors were considered. *CXCL9/10/11* or *CXCL8* foci were defined as all parenchymal cells exhibiting local *CXCL9/10/11* – positivity >= 0.65 (a) or *CXCL8*-positivity >= 0.5, (b) respectively. We considered a lower *CXCL8*-positivity cutoff to account for the gene number imbalance compared to *CXCL9/10/11*.

c, Bar graph showing the proportion of parenchymal cells assigned to either *CXCL8* or *CXCL9/10/11* foci.

d, Scatter plots showing cells within non-malignant epithelial tissues. Colors reflect expression of *CXCL8*, *CXCL9*, *CXCL10* or *CXCL11*.

e, Scatter plots showing cells within non-malignant epithelial tissues. Colors correspond to compartments and chemokine foci. Cells assigned to *CXCL9/10/11* and *CXCL8* foci are considered ambiguous.

f, Box plot showing the distribution of the Euclidean distance to the nearest stromal cell for cells assigned to *CXCL8* or *CXCL9/10/11* foci within HPV-OPSCC (n = 7) samples. The box corresponds to the interquartile range (IQR), horizontal line inside the box indicates the median, whiskers (vertical bars) extend to the smallest and largest data points within 1.5*IQR from the lower and upper quartiles, respectively, and data points beyond boundaries of whiskers reflect outliers. P-values are based on two-sided Wilcoxon rank-sum test. Source data are provided as a Source Data file.

g, Bar graph showing cell type composition of CXCL8 (top) and CXCL9/10/11 foci (bottom).

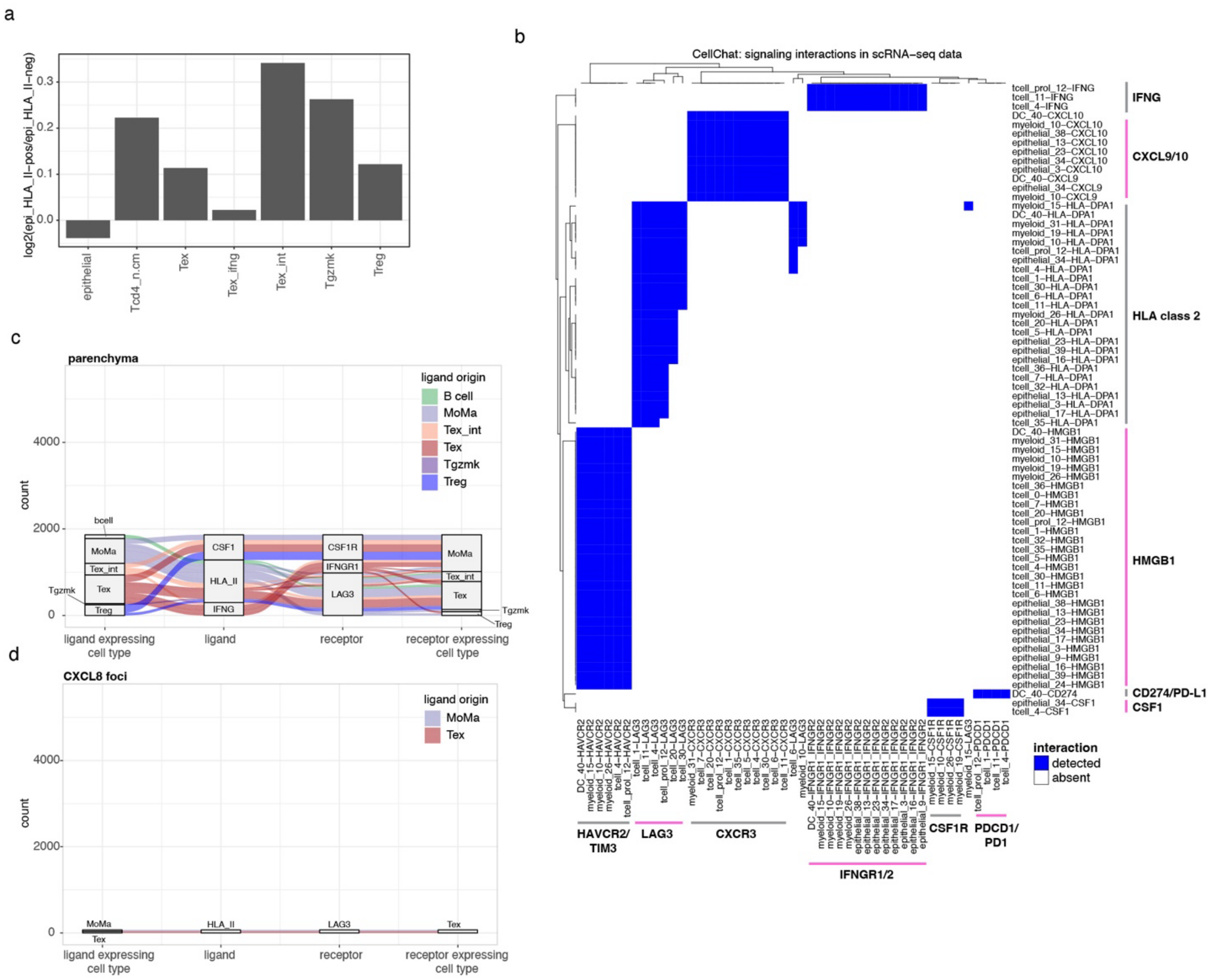

Supplementary Figure 10. Direct cell-to-cell and paracrine signaling in parenchymal chemokine compartments.

a, Bar graph showing log-2 fold change of the frequency of spatial association of the indicated cell types comparing HLA class II – positive – or – negative carcinoma cells. Only carcinoma cells expressing HPV genes were considered in this analysis.

b, Heatmap showing cell type-specific ligand-receptor interactions identified within the scRNA-seq data. Rows correspond to cell-type clusters expressing indicated ligands; columns correspond

to cell-type clusters expressing indicated receptor. Only interactions involving epithelial, myeloid and T cell clusters are shown.

c, d, Alluvial diagram corresponding to all immune cell pairs that express cognate receptor-ligand pairs and satisfy a nearest neighbor relationship. More specifically, for each ligand-expressing cell k-nearest neighbors graph was computed (k = 5) and used to identify cognate receptor-expressing neighboring cells. Interactions within parenchymal cells included in neither CXCL9/10/11 nor CXCXL8 foci (c) and CXCXL8 foci (d) are shown.

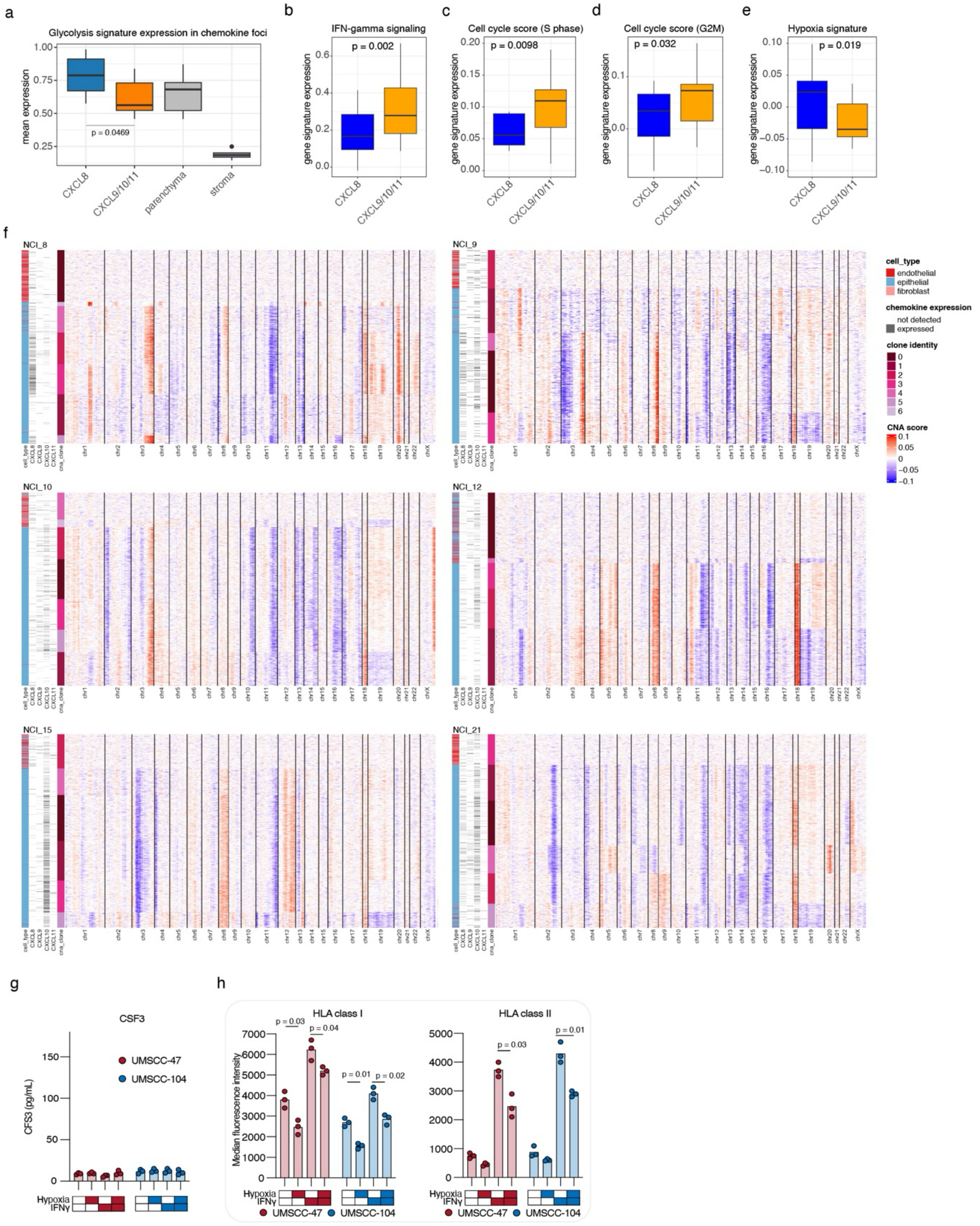

Supplementary Figure 11. Characterization of chemokine foci.

a, Boxplot showing the mean expression of a glycolysis signature within HPV-OPSCC samples (n = 7) for indicated chemokine and anatomical compartments. The box corresponds to the

interquartile range (IQR), horizontal line inside the box indicates the median, whiskers (vertical bars) extend to the smallest and largest data points within 1.5*IQR from the lower and upper quartiles, respectively, and data points beyond boundaries of whiskers reflect outliers. P-value is based on paired, two-sided Wilcoxon rank-sum test.

b-e, Boxplots showing the sample-based mean gene signature expression related to IFN-γ signaling (a), cell cycle S phase (b), cell cycle G2 to M phase (c) or hypoxia (d). The mean gene signature expression was obtained considering scRNA-seq transcriptome profiles of cells classified as epithelial that expressed either *CXCL8* or at least one of *CXCL9/10/11*. Only samples with at least 20 epithelial cells within each group (*CXCL8* and *CXCL9/10/11)* were considered in this analysis. P-values are based on paired, two-sided Wilcoxon rank-sum test.

f, Heatmaps showing inferred DNA copy number profiles in scRNA-seq of HPV-OPSCC. Endothelial cells and fibroblasts were included as control cell types in this analysis. In addition, clustering of copy number alterations (CNA) profiles revealed distinct clones that display specific copy number changes including amplifications of chromosome arm 3q and 8q in a subset of samples commonly observed in HPV-associated HNSCC[1]. In addition, clustering of individual CNA profiles revealed numerous clones characterized by specific patterns of copy-number gains and losses. For each cell, detection of *CXCL8*, *CXCL9*, *CXCL10* and *CXCL11* expression is indicated. Analysis of *CXCL8* and *CXCL9/10/11* expression across inferred genetic clones did not reveal clones that substantially differed based on expression of these chemokines, suggesting that genetic factors and clonal structure may not play a dominant role in driving chemokine expression and foci formation.

g, Bar graphs showing expression of soluble CSF3 in patient-derived HPV-positive cell lines UMSCC-47 and UMSCC-104 treated with a combination of hypoxia and IFN-γ as indicated below using red and blue rectangles.

h, Bar graphs showing cell surface expression of HLA class I and HLA class II in patient-derived HPV-positive cell lines UMSCC-47 and UMSCC-104 treated with a combination of hypoxia and IFN-γ as indicated below using red and blue rectangles. P-values are based on ANOVA with multiple comparisons. *: $p<0.05$.

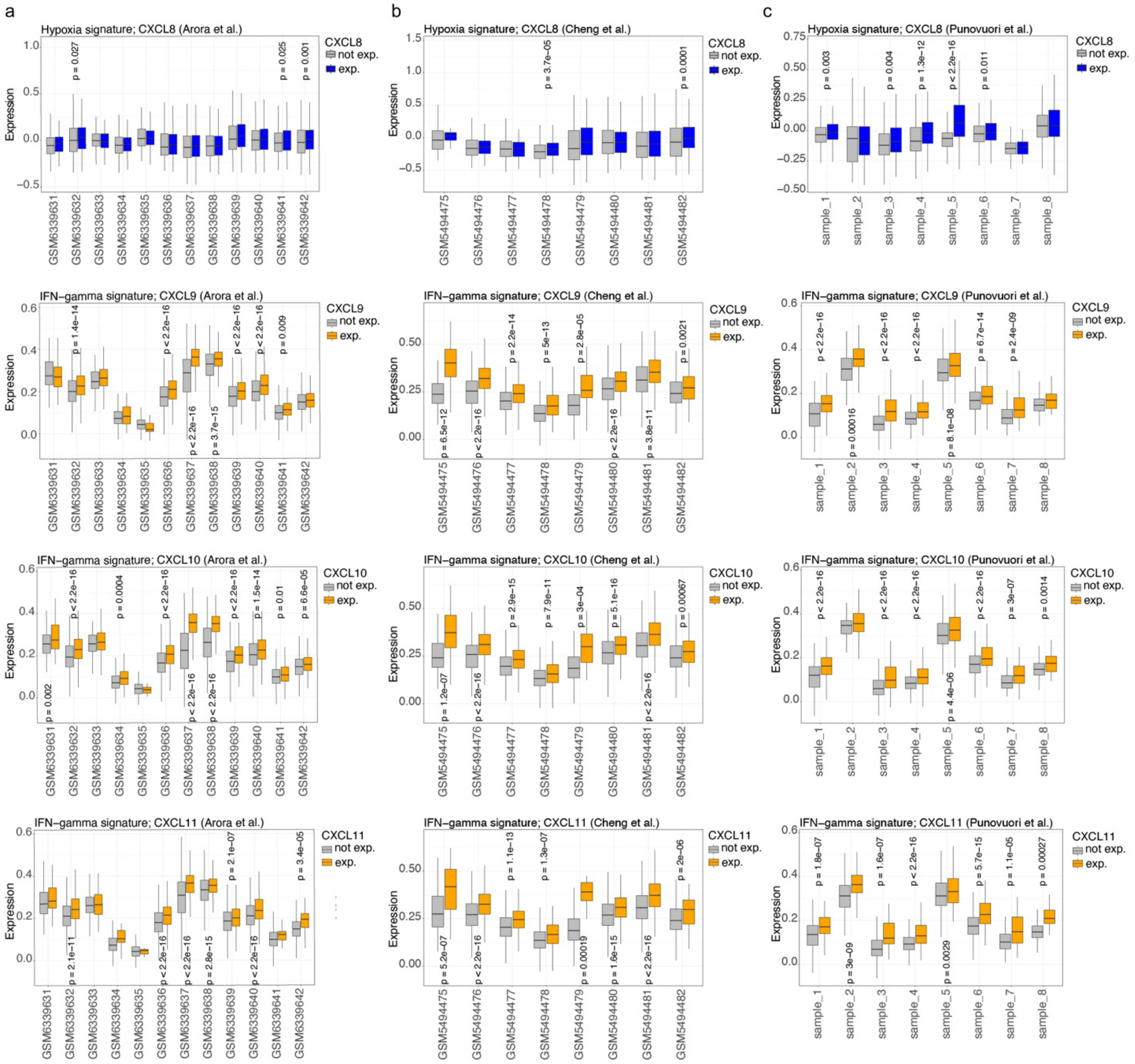

Supplementary Figure 12. HNSCC Visium data.

a-c, Boxplots showing the expression of the hypoxia signature (top row) or IFN-γ signature (rows 2 to 4) within KRT-high parenchymal Visium spots further classified based on the detection of CXCL8 (row 1), CXCL9 (row 2), CXCL10 (row 3) or CXCL11 (row 4) within multiple independent HNSCC Visium data sets published by Arora et al.[2] (a), Cheng et al.[3] (b) and Punovuori et al.[4] (c). The box corresponds to the interquartile range (IQR), horizontal line inside the box indicates the median, whiskers (vertical bars) extend to the smallest and largest

data points within 1.5*IQR from the lower and upper quartiles, respectively, and data points beyond boundaries of whiskers reflect outliers. P-values are based on two-sided Wilcoxon rank-sum test.

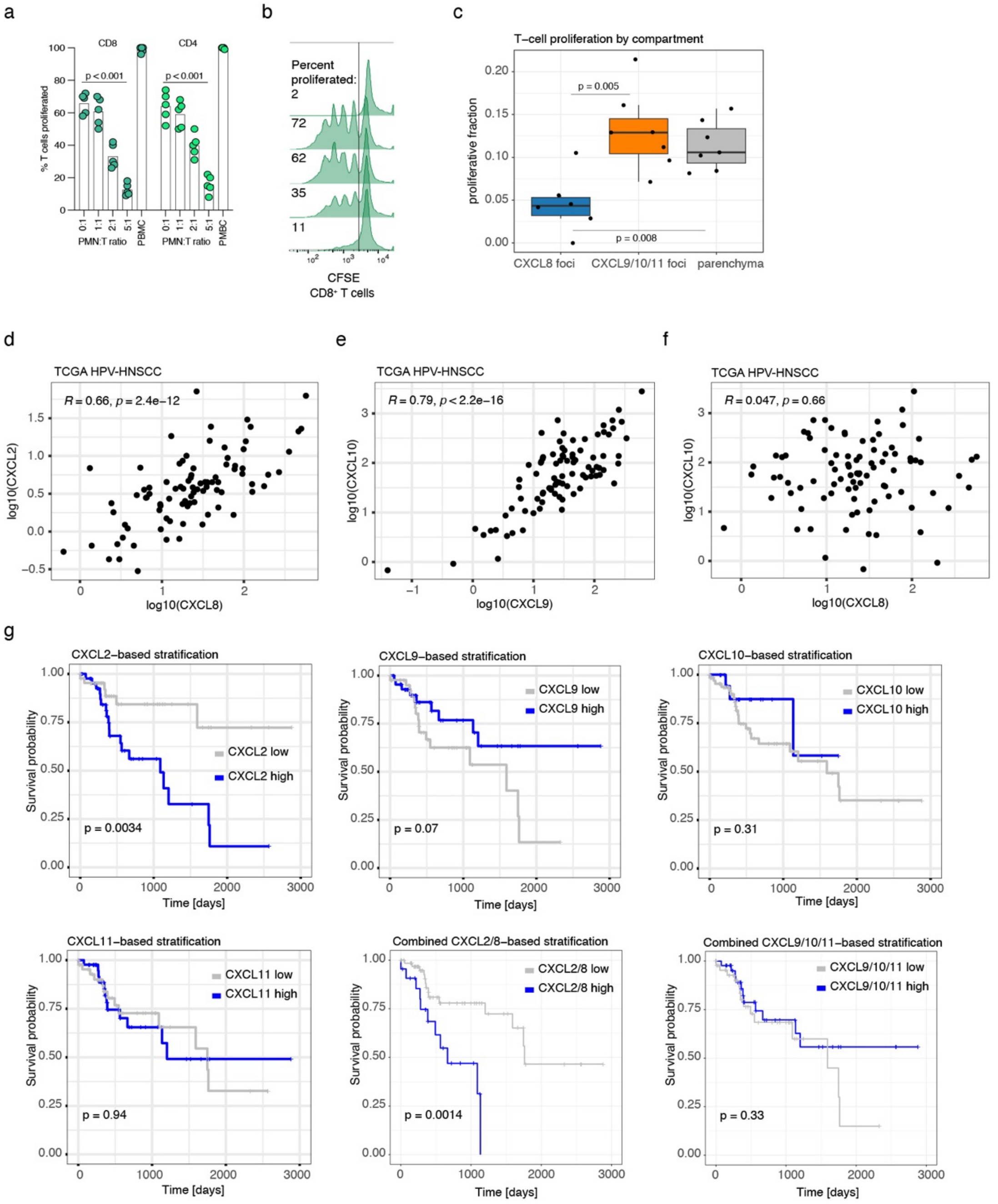

Supplementary Figure 13. Immunosuppression and HPV-HNSCC TCGA data analysis.

a, Bar graph showing fraction of activated CD8 (left) and CD4 (right) T cells undergoing proliferation during co-culture with increasing numbers of polymorphonuclear (PMN) neutrophilic cells isolated from an independent cohort of HPV-OPSCC tumors (n=5, Table S3). P-value; ANOVA.

b, Representative CFSE histograms of CD8 T cells, with or without CD3/28 stimulation, co-cultured with different ratios of tumor-infiltrating neutrophilic cells described in (a). Representative histograms are from one experiment using tumor-infiltrating neutrophils from a single patient.

c, Boxplot showing the proliferative fraction of T cells located within *CXCL8* foci, *CXCL9/10/11* foci or the remaining parenchyma considering all HPV-OPSCC samples (n = 7). The box corresponds to the interquartile range (IQR), horizontal line inside the box indicates the median, whiskers (vertical bars) extend to the smallest and largest data points within 1.5*IQR from the lower and upper quartiles, respectively, and data points beyond boundaries of whiskers reflect outliers. P-value is based on two-sided Wilcoxon rank-sum test.

d, e, f, Scatter plots showing the expression of *CXCL2* vs. *CXCL8* (a), *CXCL10* vs. *CXCL9* (b) and *CXCL10* vs *CXCL8* (c) measured as log10-transformed transcripts per million (TPM). P-values are based on Fisher's transform.

g, Line graphs showing survival probabilities over time resulting from expression-based stratification of TCGA HPV-HNSCC samples using *CXCL2* (top left), *CXCL9* (top center), *CXCL10* (top right), *CXCL11* (bottom left), combined *CXCL2* and *CXCL8* (bottom center), or combined *CXCL9*, *CXCL10* and *CXCL11* (bottom right). P-values are based on log-rank test.

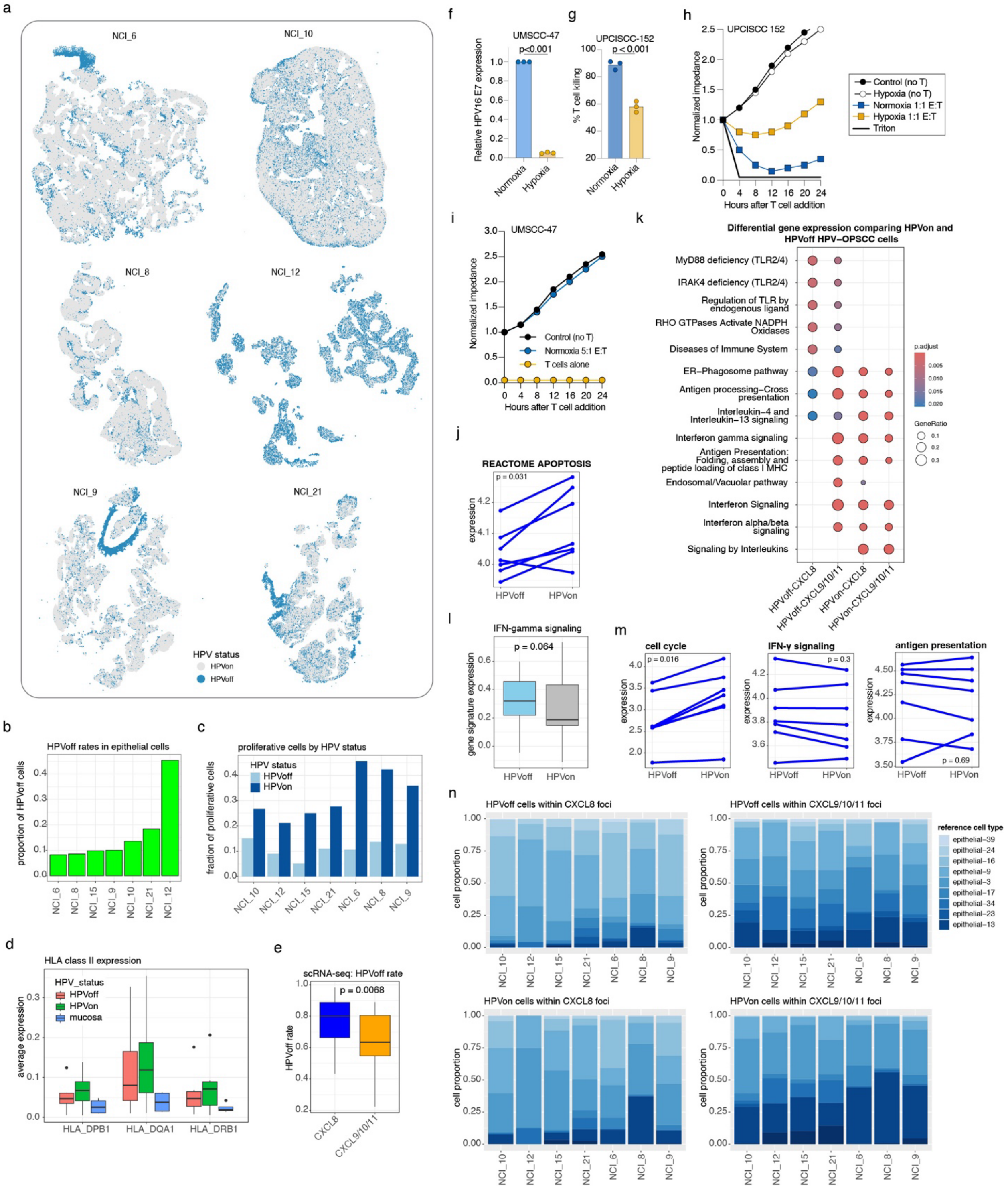

Supplementary Figure 14. Characterization HPV gene expression heterogeneity.

a, Scatter plots showing HPV-OPSCC samples. Colors reflect detected HPV transcripts.

b, Bar graph showing the proportion of epithelial cells without detectable HPV transcripts (HPVoff).

c, Bar graph showing the fraction of proliferative epithelial cells classified as HPVon or HPVoff.

d, Box plot showing the sample-based average expression of HLA class II genes within HPVoff, HPVon of HPV-OPSCC samples (n = 7) and non-malignant epithelial cells (n = 4). The box corresponds to the interquartile range (IQR), horizontal line inside the box indicates the median, whiskers (vertical bars) extend to the smallest and largest data points within 1.5*IQR from the lower and upper quartiles, respectively, and data points beyond boundaries of whiskers reflect outliers. The observed differences did not reach statistical significance.

e, Boxplot showing the sample-based HPVoff rate considering scRNA-seq transcriptome profiles of epithelial cells that expressed either *CXCL8* or at least one of *CXCL9/10/11*. Only samples with at least 20 epithelial cells within each group (*CXCL8* and *CXCL9/10/11)* were considered in this analysis. The box corresponds to the interquartile range (IQR), horizontal line inside the box indicates the median, whiskers (vertical bars) extend to the smallest and largest data points within 1.5*IQR from the lower and upper quartiles, respectively, and data points beyond boundaries of whiskers reflect outliers. P-value is based on paired Wilcoxon rank-sum test.

f, **g,** Bar graphs showing HPV16 E7 expression under normoxia and hypoxia in the HLA-A*02-negative and HPV16-positive cell line UMSCC-47 (f) and quantification of T cell-mediated killing of the HLA-A*02- and HPV16-positive cell line UPCISCC 152 following 16 hours of T cell and target cell co-culture (g). P-values are based on unpaired two-tailed t-test. Source data are provided as a Source Data file (g).

h, Line graph showing normalized impedance of patient-derived HLA-A*02- and HPV16-positive cell line UPCISCC 152, co-cultured with T cells expressing an HLA-A*02-restricted,

HPV16 E7-specific TCR in a 1:1 effector-to-target ratio. UPCISCC 152 cells were either cultured under normoxic or hypoxic conditions, prior to T cell co-culture under normoxic conditions. Of note, the T cells were not exposed to hypoxia and were maintained in normoxic conditions throughout the experiments. Growth curves of UPCISCC 152, previously exposed to normoxia or hypoxia, in the absence of T cells are depicted as well. Source data are provided as a Source Data file.

i, Line graph showing normalized impedance of the patient-derived HLA-A*02-negative HPV16-positive cell line UMSCC-47, unaffected by co-culture with T cells engineered to express an HPV16 E7-specific TCR restricted to HLA-A*02 in a 5:1 effector-to-target ratio. Impedance of T cell alone is also shown. Source data are provided as a Source Data file.

j, Line graphs showing average expression of an apoptosis gene signatures in HPVon and HPVoff HPV-OPSCC epithelial cells. Values of HPVon and HPVoff cells from the same sample are connected by solid lines. P-value is based on a paired, two-sided Wilcoxon rank-sum test.

k, Dot plot showing Reactome pathway terms enriched within genes that are differentially expressed between HPVon and HPVoff cells. Differential gene expression analysis was performed for *CXCL8*- and CXCL9/10/11 foci separately.

l, Boxplot showing the sample-based mean IFN-γ signaling signature expression obtained using scRNA-seq transcriptome profiles of cells classified as HPVon or HPVoff epithelial cells that expressed at least one of *CXCL9/10/11*. Only samples with at least 20 epithelial cells within each group (HPVon and HPVoff) were considered in this analysis. The box corresponds to the interquartile range (IQR), horizontal line inside the box indicates the median, whiskers (vertical bars) extend to the smallest and largest data points within 1.5*IQR from the lower and upper

quartiles, respectively, and data points beyond boundaries of whiskers reflect outliers. P-value is based on paired, two-sided Wilcoxon rank-sum test.

m, Line graphs showing average expression of cell cycle (left), IFN-γ signaling (center) and antigen presentation (right) gene signatures in HPVon and HPVoff HPV-OPSCC epithelial cells located within *CXCL8* foci. P-values; paired, two-sided Wilcoxon rank-sum test.

n, Bar plots showing cell-type proportions of cancer cells resulting from reference gene expression profile - based classification. Cancer cells were distinguished based on HPV expression and parenchymal localization into HPVoff (top) and HPVon (bottom) as well as *CXCL8*- (left) and CXCL9/10/11-focal (right).

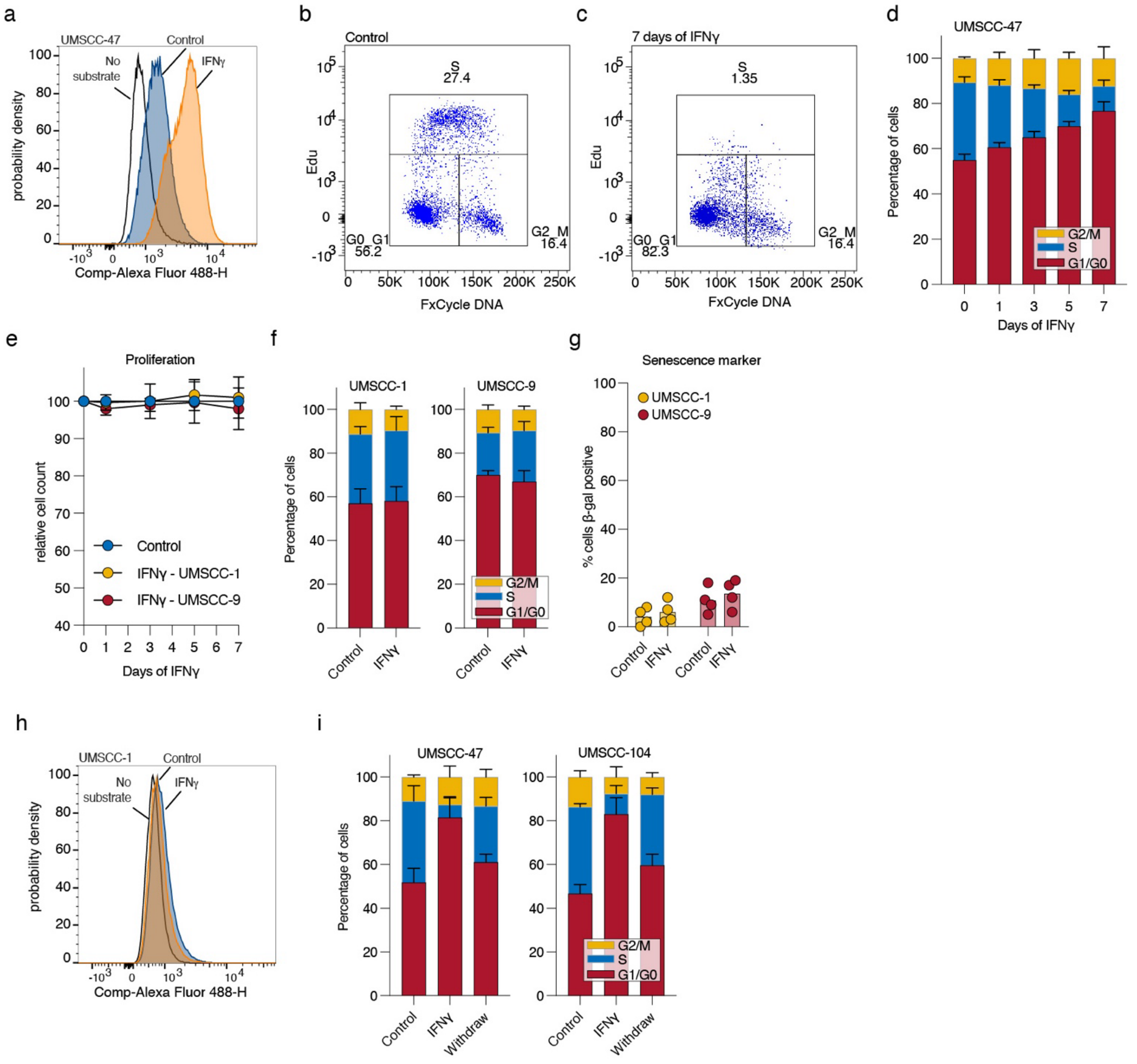

Supplementary Figure 15. IFN-γ-mediated induction of senescence.

a, Histograms showing β-galactosidase expression in HPV-positive UMSCC-47 cells treated with or without IFN-γ for 7 days. The black histogram represents the cell line assayed without the addition of the fluorescent β-galactosidase substrate as a negative control.

b,c, Flow cytometry scatter plot showing EdU incorporation and DNA content for UMSCC-47 cells untreated (j) or with IFN-γ for seven days (k).

d, Stacked bar plot showing the mean fraction of UMSCC-47 cells of three biological replicates in G1/G0, S or G2/M cell cycle phase following up to 7 days of IFN-γ treatment. Error bars correspond to standard deviation. Source data are provided as a Source Data file.
e, Line graph showing relative cell count of patient-derived HPV-negative and p53-mutant HNSCC cell lines UMSCC-1 and UMSCC-9 treated with IFN-γ relative to untreated cells. Source data are provided as a Source Data file.
f, Stacked bar plot showing the mean fraction of patient-derived HPV-negative and p53-mutant HNSCC cell lines UMSCC-1 (left) and UMSCC-9 (right) in G1/G0, S or G2/M cell cycle phase of three biological replicates following up to 7 days of IFN-γ treatment. Error bars correspond to standard deviation. Source data are provided as a Source Data file.
g, Bar graphs showing fraction of cells expressing the senescence marker β-galactosidase in UMSCC-1 and UMSCC-9 cell lines with or without 7 days of IFN-γ treatment. Source data are provided as a Source Data file.
h, Histograms showing β-galactosidase expression in HPV-positive or HPV-negative UMSCC-1 cells treated with or without IFN-γ for 7 days. The black histogram represents the cell line assayed without the addition of the fluorescent β-galactosidase substrate as a negative control.
i, Stacked bar plots showing the mean fraction of cells in G1/G0, S or G2/M cell cycle phase in untreated control cells of three biological replicates, following 7 days of IFN-γ treatment and 7 days post-IFN-γ withdrawal in HPV-positive and p53-wildtype cell lines UMSCC-47 and UMSCC-104. Error bars correspond to standard deviation. Source data are provided as a Source Data file.

Supplementary Table 1. Clinical and disease characteristics of OPSCC samples used for single-cell and spatial gene expression analysis.

ID	smoking status	primary location	HPV type	TNM	Stage	PDL1 CPS	TMB	sc-RNA-seq?	sc-SGE?
NCI_3	never	L BOT	16	T2N2	II	n/a	n/a	Yes	Yes#
NCI_4	current	L BOT	33	T2N1	I	5	n/a	Yes	Yes#
NCI_5	former	R BOT	33	T1N2	II	10	9	Yes	No
NCI_6	former	R BOT	16	T2N1	I	15	67	Yes	Yes*
NCI_7	current	R tonsil	16	T2N1	I	4	3	Yes	No
NCI_8	current	L tonsil	16	T4N2	III	2	1	Yes	Yes*
NCI_9	never	R tonsil	16	T2N1	I	4	13	Yes	Yes*
NCI_10	never	R BOT	16	T2N2	II	10	4	Yes	Yes*
NCI_11	never	R tonsil	16	T2N1	I	n/a	n/a	Yes	No
NCI_12	never	R BOT	16	T1N1	I	3	3	Yes	Yes*
NCI_13	never	R BOT	16	T3N1	II	0	1	Yes	No
NCI_14	never	L BOT	16	T1N1	I	1	5	Yes	Yes#
NCI_15	former	L BOT	16	T1N1	I	3	4	Yes	Yes*
NCI_21	former	L BOT	16	T2N1	I	10	2	Yes	Yes*
NCI_22	never	L tonsil	16	T1N2	II	n/a	n/a	Yes	Yes#

HPV, human papillomavirus; CPS, combined positive score; TMB, tumor mutation burden; sc-RNA-seq, single-cell RNA-sequencing; sc-SGE, single-cell spatial gene expression profiling
*pathologically annotated malignant samples
#pathologically annotated non-malignant mucosa

Supplementary Table 2. Clinical and disease characteristics of OPSCC samples used for *ex vivo* neutrophil function analysis.

ID	smoking status	primary location	HPV type	TNM	Stage
Pt_1	former	R tonsil	16	T2N1	I
Pt_2	never	L BOT	16	T3N1	II
Pt_3	never	L tonsil	16	T2N1	I
Pt_4	never	L tonsil	16	T2N1	I
Pt_5	never	R tonsil	16	T2N1	I

Supplementary References

1 Sievers, C. *et al.* Comprehensive multiomic characterization of human papillomavirus-driven recurrent respiratory papillomatosis reveals distinct molecular subtypes. *Commun Biol* **4**, 1416 (2021). https://doi.org/10.1038/s42003-021-02942-0

2 Arora, R. *et al.* Spatial transcriptomics reveals distinct and conserved tumor core and edge architectures that predict survival and targeted therapy response. *Nat Commun* **14**, 5029 (2023). https://doi.org/10.1038/s41467-023-40271-4

3 Cheng, H. Y. *et al.* Snail-regulated exosomal microRNA-21 suppresses NLRP3 inflammasome activity to enhance cisplatin resistance. *J Immunother Cancer* **10** (2022). https://doi.org/10.1136/jitc-2022-004832

4 Punovuori, K. *et al.* Multiparameter imaging reveals clinically relevant cancer cell-stroma interaction dynamics in head and neck cancer. *Cell* **187**, 7267-7284 e7220 (2024). https://doi.org/10.1016/j.cell.2024.09.046
