# Supplementary material for: Spatial cellular order underlies locally-confined mechanisms of immune resistance in oropharyngeal cancer
Source: Nat Commun. 2026 Jun 13;17:7512. doi: 10.1038/s41467-026-74318-z (PMC13408450; doi:10.1038/s41467-026-74318-z)
Supplement: Supplementary file 2 — Description of Additional Supplementary Files [file 41467_2026_74318_MOESM2_ESM.pdf]

Description of Additional Supplementary Files

Supplementary Dataset 1: Xenium probe genes
