# Supplementary material for: Spatial cellular order underlies locally-confined mechanisms of immune resistance in oropharyngeal cancer
Source: Nat Commun. 2026 Jun 13;17:7512. doi: 10.1038/s41467-026-74318-z (PMC13408450; doi:10.1038/s41467-026-74318-z)
Supplement: Supplementary file 4 — Reporting Summary [file 41467_2026_74318_MOESM4_ESM.pdf]

nature portfolio

Corresponding author(s): Cem Sievers, Clint Allen and Charalampos Floudas

Last updated by author(s): 10/30/2025

Reporting Summary

Nature Portfolio wishes to improve the reproducibility of the work that we publish. This form provides structure for consistency and transparency in reporting. For further information on Nature Portfolio policies, see our Editorial Policies and the Editorial Policy Checklist.

Statistics

For all statistical analyses, confirm that the following items are present in the figure legend, table legend, main text, or Methods section.

n/a	Confirmed	
☐	☒	The exact sample size (*n*) for each experimental group/condition, given as a discrete number and unit of measurement
☐	☒	A statement on whether measurements were taken from distinct samples or whether the same sample was measured repeatedly
☐	☒	The statistical test(s) used AND whether they are one- or two-sided<br>*Only common tests should be described solely by name; describe more complex techniques in the Methods section.*
☐	☒	A description of all covariates tested
☐	☒	A description of any assumptions or corrections, such as tests of normality and adjustment for multiple comparisons
☐	☒	A full description of the statistical parameters including central tendency (e.g. means) or other basic estimates (e.g. regression coefficient) AND variation (e.g. standard deviation) or associated estimates of uncertainty (e.g. confidence intervals)
☐	☒	For null hypothesis testing, the test statistic (e.g. *F*, *t*, *r*) with confidence intervals, effect sizes, degrees of freedom and *P* value noted<br>*Give P values as exact values whenever suitable.*
☒	☐	For Bayesian analysis, information on the choice of priors and Markov chain Monte Carlo settings
☒	☐	For hierarchical and complex designs, identification of the appropriate level for tests and full reporting of outcomes
☒	☐	Estimates of effect sizes (e.g. Cohen's *d*, Pearson's *r*), indicating how they were calculated

Our web collection on statistics for biologists contains articles on many of the points above.

Software and code

Policy information about availability of computer code

Data collection	single-cell RNA-seq data was analyzed with cellranger (version 7.1.0); Xenium data was analyzed with spaceranger (version 1.7.6.0); data was analyzed with R (version 4.1.2)
Data analysis	Code for data analysis was written in R (version 4.1.2)

For manuscripts utilizing custom algorithms or software that are central to the research but not yet described in published literature, software must be made available to editors and reviewers. We strongly encourage code deposition in a community repository (e.g. GitHub). See the Nature Portfolio guidelines for submitting code & software for further information.

Data

Policy information about availability of data

All manuscripts must include a data availability statement. This statement should provide the following information, where applicable:

- Accession codes, unique identifiers, or web links for publicly available datasets
- A description of any restrictions on data availability
- For clinical datasets or third party data, please ensure that the statement adheres to our policy

Raw and processed data used in this study is available through Gene Expression Omnibus using accession numbers GSE290040 (scRNA-seq) and GSE290041 (Xenium).

Research involving human participants, their data, or biological material

Policy information about studies with human participants or human data. See also policy information about sex, gender (identity/presentation), and sexual orientation and race, ethnicity and racism.

Reporting on sex and gender	Patient gender is not reported as indirect identifiers.
Reporting on race, ethnicity, or other socially relevant groupings	Race is not reported as indirect identifiers.
Population characteristics	Disease characteristics of each patient are included in Table S1.
Recruitment	Subjects were enrolled onto the biospecimen collection protocol following full informed consent at the time of a clinically indictaed procedure.
Ethics oversight	This study complies with all relevant ethical regulations. The biospecimen protocol used to collect these samples was approved by the NIH Clinical Center IRB.

Note that full information on the approval of the study protocol must also be provided in the manuscript.

Field-specific reporting

Please select the one below that is the best fit for your research. If you are not sure, read the appropriate sections before making your selection.

☒ Life sciences ☐ Behavioural & social sciences ☐ Ecological, evolutionary & environmental sciences

For a reference copy of the document with all sections, see nature.com/documents/nr-reporting-summary-flat.pdf

Life sciences study design

All studies must disclose on these points even when the disclosure is negative.

Sample size	no sample-size calculation was performed
Data exclusions	Poor quality data that did not meet the quality requirements was excluded. Criteria for exclusion were not predefined, but rather based on data evaluation.
Replication	Orthogonal data was used to validate the main findings of this study.
Randomization	no randomization was performed.
Blinding	n/a

Reporting for specific materials, systems and methods

We require information from authors about some types of materials, experimental systems and methods used in many studies. Here, indicate whether each material, system or method listed is relevant to your study. If you are not sure if a list item applies to your research, read the appropriate section before selecting a response.

Materials & experimental systems

n/a	Involved in the study	
☐	☒	Antibodies
☐	☒	Eukaryotic cell lines
☒	☐	Palaeontology and archaeology
☒	☐	Animals and other organisms
☐	☒	Clinical data
☒	☐	Dual use research of concern
☒	☐	Plants

Methods

n/a	Involved in the study	
☒	☐	ChIP-seq
☐	☒	Flow cytometry
☒	☐	MRI-based neuroimaging

Antibodies

Antibodies used	Anti-human pan HLA class I (clone W6/32), anti-human HLA-DR (L243), anti-human CD8 (SK1) and anti-human CD4 (GK1.5) for flow cytometry; CD3 (OKT3), CD38 (CD28.2) for T cell stimulation, and anti-human pancytokeratin (AE1/AE3), CD8 (EPR10640-2), PD-1

(EPR4877) and Tim3 (D5D5R) for immunofluorescence
Antibody Vendor /Clone Catalog # Dilution 1: HIER Secondary Antibody Opal Opal dilution 1:
Tim3 CST[D5D5R] ab245620 25 Leica ER2(AR9640) / 20 min BioCare MACH 2 Rabbit HRP-Polymer (RHRP520) 520 150
PD-1 Abcam [EPR4877(2)] ab137132 750 Leica ER2(AR9640) / 20 min Akoya OPAL POLYMER HRP MS + RB, 1X, (ARH1001EA) 570 150
CD8 Abcam [EPR10640-2] ab215041 2000 Leica ER2(AR9640) / 20 min Akoya OPAL POLYMER HRP MS + RB, 1X, (ARH1001EA) 480 150
PanCK Santa Cruz [AE1/AE3] sc-81714 400 Leica ER2(AR9640) / 20 min Akoya OPAL POLYMER HRP MS + RB, 1X, (ARH1001EA) 780 50

Validation

All antibodies used have been commercially validated.

Eukaryotic cell lines

Policy information about cell lines and Sex and Gender in Research

Cell line source(s)

University of Michigan and University of Pittsburgh under material transfer agreements (UMSCC-1, UMSCC-9, UMSCC-104, UMSCC-47, UPCISCC-152).

Authentication

Cell lines have been previously authenticated with genomic sequencing in our laboratory.

Mycoplasma contamination

Serially tested to ensure mycoplasma negativity.

Commonly misidentified lines
(See ICLAC register)

None

Clinical data

Policy information about clinical studies
All manuscripts should comply with the ICMJE guidelines for publication of clinical research and a completed CONSORT checklist must be included with all submissions.

Clinical trial registration

NCT03429036 (ClinicalTrials.gov)

Study protocol

The full biospecimen protocol can be made available upon request to the corresponding authors.

Data collection

Samples were collected from patients undergoing standard of care procedures.

Outcomes

n/a

Plants

Seed stocks

n/a

Novel plant genotypes

n/a

Authentication

n/a

Flow Cytometry

Plots

Confirm that:

☒ The axis labels state the marker and fluorochrome used (e.g. CD4-FITC).

☒ The axis scales are clearly visible. Include numbers along axes only for bottom left plot of group (a 'group' is an analysis of identical markers).

☒ All plots are contour plots with outliers or pseudocolor plots.

☒ A numerical value for number of cells or percentage (with statistics) is provided.

Methodology

Sample preparation

Described in the methods of the manuscript on page 32

Instrument	BD Fortessa
Software	FACS Diva and FlowJo
Cell population abundance	Described in the methods of the manuscript on page 32
Gating strategy	Described in the methods of the manuscript on page 32

☒ Tick this box to confirm that a figure exemplifying the gating strategy is provided in the Supplementary Information.
