# Supplementary material for: Spatial cellular order underlies locally-confined mechanisms of immune resistance in oropharyngeal cancer
Source: Nat Commun. 2026 Jun 13;17:7512. doi: 10.1038/s41467-026-74318-z (PMC13408450; doi:10.1038/s41467-026-74318-z)
Supplement: Supplementary file 5 — Transparent Peer Review file [file 41467_2026_74318_MOESM5_ESM.pdf]

nature portfolio

Peer Review File

Spatial cellular order underlies locally-confined mechanisms of immune resistance in oropharyngeal cancer

Corresponding Author: Dr Cem Sievers

This file contains all reviewer reports in order by version, followed by all author rebuttals in order by version.

Attachments originally included by the reviewers as part of their assessment can be found at the end of this file.

Version 0:

Reviewer comments:

Reviewer #1

(Remarks to the Author)
Sievers et al. conducted an in-depth study using single-cell and spatial transcriptomic analyses to dissect the immune architecture and resistance mechanisms in oropharyngeal squamous cell carcinoma (OPSCC). The study distinguishes immune foci, reveals HPV-negative (HPV-) tumor cell phenotypes, and highlights potential mediators of immune evasion. While the work is novel and comprehensive, several critical points require clarification, additional analysis, and improved data presentation.

1. Despite demonstrating HPV gene expression confined to tumor parenchyma (Fig. 1h), many epithelial clusters appear in both tumor and normal conditions (Fig. 2a, Fig. S5), raising concerns about clustering accuracy or insufficient resolution from spatial gene sets (~297 genes). Additional stromal markers (e.g., fibroblast/endothelial markers) in spatial clustering heatmaps (e.g., Fig. 1d) are needed to better illustrate regional demarcation. Further analysis is recommended to clarify cell type classification strategy and consider re-annotation or refinement of epithelial cluster assignments.
2. CXCR2 expression is higher in monocytes/macrophages than in neutrophils (Fig. 3g), yet only neutrophils enrich in CXCL8 foci (Fig. 3f). This discrepancy requires explanation. Since CXCL8 may not be the dominant factor recruiting neutrophils or reducing T cell proliferation (Figs. 2g, 4h), reconsider the naming of "CXCL8-foci" to avoid confusion.
3. Neutrophils express both ligands (CXCL2, CXCL8, CSF3) and receptors (CXCR2, CSF3R). Discuss potential autocrine signaling and self-recruitment mechanisms.
4. Coculture experiments suggest neutrophils suppress T cell proliferation. Validate this function in spatial data by confirming reduced proliferation in T cells localized to CXCL8 foci.
5. HPV- tumor cells in both CXCL10 and CXCL8 foci share features like low HPV gene expression and reduced proliferation. Clarify how much these phenotypes arise from microenvironmental cues vs. intrinsic viral gene regulation loss. Moreover, the differentiation status and lineage relationship between HPV+ and HPV- cells, and whether these differ between foci types are unclear.
6. While myeloid HLA-II expression is linked to T cell exhaustion (Fig. 3h), prior studies (e.g., PMID: 39105803) have reported HLA-II expression in HPV+ tumor cells. Include analysis on HPV+ tumor cell HLA-II expression and its contribution to T cell dysfunction.
Additionally, HPV- tumor cells show stronger antigen presentation (Fig. 5g), which contradicts prior findings. Reassess and clarify result reliability.
7. Since CXCL10 and CXCL8 foci can co-exist in the same tumor, analyze inter-patient variability in foci proportion and whether this correlates with clinical outcomes. Consider using public datasets (e.g., PMIDs: 37012457, 38857913, 39105803) or TCGA-HNSCC to validate key findings and assess prognostic significance.
8. Chemokine signaling analysis is currently limited to tumor and immune cells. Examine the role of stromal cells (e.g., fibroblasts, endothelial cells) in ligand/receptor expression and immune modulation.
9. Conclusions regarding the role of hypoxia and inflammatory signaling in shaping immune foci (CXCL8, CXCL10) require validation in an independent cohort or external datasets.
10. Figure presentation and layout issue:
- The ordering and layout of Figures 1 and 5 disrupt logical flow. Reorganize figures for improved narrative coherence and readability.
- The workflow diagram (Fig. 1a) should include number of patients and clarify sample pairing.

- Use standardized terminology consistently across text and figures (e.g., “malignant/non-malignant” vs. “HPV-OPSCC/normal”).

Reviewer #2

(Remarks to the Author)
I co-reviewed this manuscript with one of the reviewers who provided the listed reports. This is part of the Nature Communications initiative to facilitate training in peer review and to provide appropriate recognition for Early Career Researchers who co-review manuscripts.

Reviewer #3

(Remarks to the Author)
In this manuscript by Sievers and colleagues, they described the spatial transcriptomic landscape of HPV-OPSCC by integrating single cell RNA sequencing (5'-GEM) and 10X Xenium in-situ. In addition, the mechanisms underlying immune escape despite the presence of HPV antigens were examined in the spatial context. A cohort of 15 HPV-OPSCC samples were included in study. Seven HPV16-positive HPV-OPSCC samples and four adjacent non-malignant mucosa samples were profiled spatially for immune cell organization, cancer cell states, and cellular interactions.

While the single cell study is not considered novel, the data generated was primarily for cell type annotations especially the immune cells found in the tumours. Subsequently they used this information to design a custom panel of 297 genes for the spatial annotations and analysis. While HPVoff phenomenon has already been published, they identified distinct CXCL9/10/11 foci and CXCL8 foci which appear to be novel.

I have some comments and suggestions:

1) The majority of the data presented are descriptive and based on a very small number of samples using spatial gene expression data. While some of the observations are potentially interest, my main concern would be of false positives due to the small sample size and lack of statistical consideration for multiple testing. Findings of immune compartment differences between tumor and normal samples, for example in Fig 2e-h, should be validated in an independent cohort or confirmed in an orthogonal assay. Some of the cell types described (Tex, Treg) appear to be very low by proportion.

2) Fig 3h is too complicated to comprehend. They may want to move it to supplementary or replace it with simpler diagrams to highlight the main important observations.

3) For data presented in figures 4 & 5, it is clear that samples NCI-8 and NCI-10, their HPV transcripts count were very low in CXCL9/10/11 foci in addition to CXCL8 foci. Therefore results presented were not consistent across the 7 malignant samples. They did partially attempt to address this in figure 5 but due to the small number of malignant samples (n=7), they need to confirm their findings on CXCL9/10/11 foci and CXCL8 foci in an independent cohort ideally using Xenium. If not, minimum requirement would be to perform immunofluorescence to determine whether:
- CXCL8 foci are depleted of HPV expression, enriched for HIF proteins or other hypoxia markers and depleted of proliferation markers (e.g.MKI-67).
- CXCL9/10/11 foci are enriched for markers for IFN gamma as well as to differentiate HPVon and HPVoff regions as some of their current samples (NCI-8 and NCI-10) showed significant reduction in HPV protein expression.
- If they are able to show that these distinct foci are statistically associated with the phenomenon described, I would be in favour of this manuscript getting published in Nat Comm.

4) In the in vitro study on the effect of hypoxia on T-cell mediated tumor cell killing, what was the corresponding effect of hypoxia on the E7-specific T cells?

MINOR comments
1) Figure 4H: define which group the yellow and white boxes refer to

Reviewer #4

(Remarks to the Author)
I co-reviewed this manuscript with one of the reviewers who provided the listed reports. This is part of the Nature Communications initiative to facilitate training in peer review and to provide appropriate recognition for Early Career Researchers who co-review manuscripts.

Reviewer #5

(Remarks to the Author)
This study by Sievers et al, integrates single-cell RNA sequencing (scRNA-seq) and single-cell spatial gene expression (SGE) profiling to investigate how cellular states and immune organization contribute to HPV-associated oropharyngeal squamous cell carcinoma (HPV-OPSCC). Applying a specific 297-gene panel (derived from scRNAseq) onto biopsies from seven HPV16-positive tumour samples and four non-malignant mucosal regions, using the Xenium spatial platform, the

authors explore various neighbour hood analyses: gross (stroma vs parenchyma), chemokin-derived and traits-based neighbourhoods.They show that spatially distinct chemokine-defined immune “foci” (CXCL9/10/11 vs CXCL8) exist and appear to influence immune and tumor cell composition. They also identify HPV transcript-negative (“HPVoff”) senescent-like cancer cells that may evade HPV-specific immunity. The team conducted a numner of mechanistic experiments that further implicate hypoxia and IFN-γ in shaping these tumor states.
The experiments conducted in this study are interesting, relevant and provide some interesting insights into the spatial organisation of HPV-positive tumors. The data shown here also add a valuable resource for re-analyses (both the scRNAseq and spatial analyses). There are a few issues that need to be addressed as listed below, and listed according to the sequence they appear in the manuscript, and I have used the Figure numbers to refer to these.

1. Figure 1 shows the scRNAseq data and derivation of the panel used for spatial analyses. The author use the latter to divide tumors in to ‘parenchymal’ and ‘stromal’ regions, and how HPV gene expression varies between these, including showing proliferation markers. There are few things to note here:
a. I wonder if the classification of ‘parenchymal’ and ‘stromal’ is the right terminology here..it sounds like tumor core, and tumor adjacent may be more appropriate. ‘Advancing front’ is also a useful term although these are not resections, so I am not sure if that is appropriate as these are biased samples as they are biopsies and may not represent the true tumor-stroma interface.
b. Reliance only on keratin-based classification may oversimplify intermediate states that may be present in the tumor such as cells undergoing EMT, especially at the stromal–epithelial interface, and the biological significance of proliferating stromal cells remains to be clarified.
2. Figure 2 maps scRNA-seq-defined cell states onto single-cell spatial gene expression for cell type annotation. They take the T/NK cells from stromal and parenchymal compartments and cluster and perform subtype annotations. Howere, here the T cells from stromal and parenchymal compartments were clustered and annotated separately prior to proportion comparisons. This biases subsequent analyses and introduces artificial differences that arise because they were analysed separately to begin with. Moreover, annotation consistency across compartments can be difficult to guarantee, direct comparisons of proportions may be biased by context-specific clustering, and cross-compartment relationships between T cell states may be obscured. A combined clustering and annotation strategy followed by stratified comparisons could have provided a more standardized framework for assessing proportional differences.

3. Figure 3 explores the chemokine landscape within HPV-OPSCC parenchyma, highlighting the emergence of spatially distinct chemokine “foci.” The authors show that T- and myeloid-cell attracting chemokines (CXCL9/10/11 and CXCL8) are not uniformly expressed, but instead form focal clusters with variable magnitude across tumours.

a. I fail to understand how they spefically zoomed in on this chemokine niche over other markers or cell types
b. The authors also do not explain details on how they derive their neighbourhood and how they correct for multiple hypothesis testing.
c. The assignment of cells to foci is based on local transcript enrichment, which may be sensitive to resolution limits of the platform.
d. The functional consequences of focal versus uniform chemokine expression are inferred but not directly tested, and whether these spatial chemokine niches actively direct cell migration or simply reflect regions of immune cell residence is unclear.
e. Moreover, the dominance of tumour cells as major chemokine producers complicates interpretation, as it raises the possibility that foci formation may represent tumour-intrinsic programs rather than solely immune-driven processes.
f. Do these chemokine niches correlate with cell type specific niches? Are you showing a difference between a neutrophil rich versus lymphocyte rich neighbourhoods, where the chemokines are the effect and not the cause?
g. Ligand-receptor analyses should be cautioned as being quite speculative
4. Figure 4 demonstrates that tumour-intrinsic gene programs, demonstrating that cancer cells within CXCL9/10/11 foci express IFN-γ–response and antigen presentation genes, whereas CXCL8-foci cells exhibit hypoxia and glycolysis signatures, reduced HPV and proliferation gene expression, and increased inflammatory cytokines that recruit neutrophils. The in vitro experiments support this notion quite nicely, although it is important to caution that we don’t quite understand which is the ‘chicken’ and which is the ‘egg’ (if it even matters!). Nevertheless, these results underscore how tumour cells actively shape spatially segregated immunological niches through conserved, environment-dependent transcriptional programs.
5. Figure 5 identifies HPVoff cells as a senescent-like tumour subpopulation that is enriched within CXCL8 foci and influenced by microenvironmental cues (maybe this is the ‘chicken!). However, while this data highlights hypoxia- and IFN-γ–driven emergence of the HPVoff phenotype, these observations are largely correlative, and additional microenvironmental factors may contribute. The functional consequences of HPVoff cells, including immune evasion and SASP-mediated modulation, require further in vivo validation, and the p53 dependence of IFN-γ–induced senescence may limit generalizability across tumours with variable p53 status or with other HPV types

Minor issues:
1. Issued with batch correction and probe bias in Xenium need to be better discussed and laid out
2. Please confirm all the statistical analyses (Wilcoxon vs t-test) as some may be inconsistently applied across figures; authors should also clarify multiple testing correction.
3. Expanding these to other HPV types- do you expect these to be different? Perhaps a discussion on this
4. Finally, a Discussion could on therapeutic implications, e.g., senolytic therapies, hypoxia-targeting, or LAG-3 blockade.

Version 1:

Reviewer comments:

Reviewer #1

(Remarks to the Author)
The authors have provided a revision with additional data and improved writing, yet several concerns remain to be addressed.
1. Clarification of epithelial cell clusters: The explanation regarding normal versus tumor epithelial cells remains unclear. Normal epithelial cells in non-malignant tissues should differ from tumor epithelial cells in HPV-OPSCC based on HPV expression and transcriptional profiles (e.g., Fig. S5), yet Figure 2A shows similar epithelial cluster distributions in both tissue types. The authors should specify which of the 10 epithelial clusters represent normal versus tumor cells and clarify the differences in their distributions between non-malignant epithelium and OPSCC tissues.
2. HPV+ vs. HPV- Tumor Cell Comparisons: While HPV+ and HPV- OPSCC may represent different diseases, comparisons between HPVon and HPVoff tumor cells (both from HPV+ OPSCC) are still necessary. Previous studies have shown that such distinctions have functional significance, as in EBV+ nasopharyngeal carcinoma (see PMID: 33531485). The authors should conduct a similar analysis and discuss how viral antigen presentation relates to tumor immune responses.
3. The newly added single-gene prognostic and correlation analyses are promising, but module score-based analyses for CXCL2/8 and CXCL9/10/11 should be performed to further strengthen the findings.
4. The authors should integrate their explanation regarding technical limitations directly into the manuscript.
5. Mechanism underlying neutrophil aggregation in CXCL8-foci remains insufficiently explained. Even considering the CSF3-CSF3R axis, macrophages express CSF3R at levels comparable to or higher than Moma_Neu, yet are less prevalent within CXCL8-foci. This discrepancy should be further clarified.
6. While lineage-tracing experiments in animal models are challenging, the authors could explore the differentiation relationship between HPV-on and HPV-off cells using pseudotime differentiation analysis with single-cell or spatial transcriptomic data.
7. HPV-Positive Cells and T-Cell Associations: HPV-positive cells show increased spatial associations with nearly all T-cell subpopulations, including cytotoxic T cells (with higher association than some Tex subclusters). This observation is broad and does not clarify the relationship with T-cell dysfunction. More specific analyses using single-cell or spatial transcriptomic data to investigate interactions or direct correlations with Tex exhaustion features would be more informative.

Reviewer #2

(Remarks to the Author)
I co-reviewed this manuscript with one of the reviewers who provided the listed reports. This is part of the Nature Communications initiative to facilitate training in peer review and to provide appropriate recognition for Early Career Researchers who co-review manuscripts.

Reviewer #3

(Remarks to the Author)
The authors have provided an adequate rebuttal. I have no further comments.

In addition, since Reviewer #5 was unavailable to complete the review this time, I was asked by the handling editor, Dr. Lizhao Wu, to comment on the concerns that Reviewer #5 raised, and to determine whether in my opinion the authors have adequately addressed Reviewer #5's concerns in the revised manuscript. Here is my response:

***
I have read through the comments of Reviewer #5 and the authors' replies. All the replies are well taken, and the comments have been adequately addressed. Several comments concerning internal validation and caution of overinterpreting the data also overlap with those from other reviewers.

These study limitations have been addressed in the Discussion section. Overall, I support publication of this manuscript.
***

Reviewer #4

(Remarks to the Author)
I co-reviewed this manuscript with one of the reviewers who provided the listed reports. This is part of the Nature Communications initiative to facilitate training in peer review and to provide appropriate recognition for Early Career Researchers who co-review manuscripts.

Version 2:

Reviewer comments:

Reviewer #1

(Remarks to the Author)
All previous concerns have been well addressed.

Reviewer #2

(Remarks to the Author)
I co-reviewed this manuscript with one of the reviewers who provided the listed reports. This is part of the Nature Communications initiative to facilitate training in peer review and to provide appropriate recognition for Early Career Researchers who co-review manuscripts.

Open Access This Peer Review File is licensed under a Creative Commons Attribution 4.0 International License, which permits use, sharing, adaptation, distribution and reproduction in any medium or format, as long as you give appropriate credit to the original author(s) and the source, provide a link to the Creative Commons license, and indicate if changes were made.
In cases where reviewers are anonymous, credit should be given to 'Anonymous Referee' and the source.
The images or other third party material in this Peer Review File are included in the article's Creative Commons license, unless indicated otherwise in a credit line to the material. If material is not included in the article's Creative Commons license and your intended use is not permitted by statutory regulation or exceeds the permitted use, you will need to obtain permission directly from the copyright holder.
To view a copy of this license, visit https://creativecommons.org/licenses/by/4.0/

Spatial cellular order underlies locally-confined mechanisms of immune resistance in oropharyngeal cancer (NCOMMS-25-38811-T)

Reviewer comments:

First, we would like to express our sincere gratitude to the anonymous reviewers. We highly appreciate the thoughtful and constructive feedback we received, which, in our opinion, significantly improved this manuscript and supported our conclusions.

Reviewer #1 (Remarks to the Author)

Sievers et al. conducted an in-depth study using single-cell and spatial transcriptomic analyses to dissect the immune architecture and resistance mechanisms in oropharyngeal squamous cell carcinoma (OPSCC). The study distinguishes immune foci, reveals HPV-negative (HPV-) tumor cell phenotypes, and highlights potential mediators of immune evasion. While the work is novel and comprehensive, several critical points require clarification, additional analysis, and improved data presentation.

1. Despite demonstrating HPV gene expression confined to tumor parenchyma (Fig. 1h), many epithelial clusters appear in both tumor and normal conditions (Fig. 2a, Fig. S5), raising concerns about clustering accuracy or insufficient resolution from spatial gene sets (~297 genes). Additional stromal markers (e.g., fibroblast/endothelial markers) in spatial clustering heatmaps (e.g., Fig. 1d) are needed to better illustrate regional demarcation. Further analysis is recommended to clarify cell type classification strategy and consider re-annotation or refinement of epithelial cluster assignments.

We agree with the reviewer that number of genes available for single-cell spatial gene expression (SGE) profiling (297) may only allow for limited resolution of different cell types and/or physiological states. In addition, these genes were selected with a particular focus on different immune cells and states. To assign major cell types (e.g. epithelial, myeloid endothelial cells) within the SGE data, we used reference gene expression profiles derived from HPV-OPSCC single-cell RNA-seq (scRNA-seq) data sets. Notably, these scRNA-seq reference gene expression profiles were able to resolve major cell types in both HPV-OPSCC and non-malignant epithelium suggested by specific expression of select marker genes (Figure 2b). Importantly, we would also like to emphasize that we have ensured that the computational annotation of stromal and epithelial/parenchymal compartments agreed well with the histologic annotation performed by a board-certified pathologist.

To address this comment, we have now included the expression of epithelial and stromal markers across the different compartments in the revised Extended Data Figure 2c, as suggested by the reviewer. This analysis revealed a clear separation by compartment further supporting the validity of our keratin-based classification of the stromal and epithelial/parenchymal compartments.

The following text was included in the revised manuscript on page 5:

“We classified individual cells with low or high proximal keratin-positivity as stromal or parenchymal cells, respectively, in agreement with histologic sample annotation and expression of epithelial and stromal marker genes (Fig, 1e,f, Extended Data Fig. 2a-c, 3)”

We thank the reviewer for this suggestion.

2. CXCR2 expression is higher in monocytes/macrophages than in neutrophils (Fig. 3g), yet only neutrophils enrich in CXCL8 foci (Fig. 3f). This discrepancy requires explanation. Since CXCL8 may not be the dominant factor recruiting neutrophils or reducing T cell proliferation (Figs. 2g, 4h), reconsider the naming of "CXCL8-foci" to avoid confusion.

We thank the reviewer for this comment. We named both *CXCL8*- and *CXCL9/10/11*-chemokine foci solely based on the focal accumulation of the respective chemokine transcripts. The naming of these foci reflects our initial motivation for examining the spatial distribution of the respective chemokines rather than encompassing downstream findings. However, we do not intend to suggest that these chemokines are the only relevant chemotactic factors within these parenchymal regions. We focused on *CXCL8* and *CXCL9/10/11* to better understand mechanisms underlying immune cell infiltration into the parenchyma. We agree with the reviewer that the mechanisms leading to the enrichment of different immune cell populations within either *CXCL8* or *CXCL9/10/11* foci are not fully understood and likely depend on an interplay of chemotactic signaling pathways. While our analysis indicates that the main sources of *CXCL8* are both epithelial as well as neutrophilic cells within CXCL8-foci, the recruitment of neutrophils may depend on additional factors. To better understand such dynamics, future work may focus on a more comprehensive spatial expression characterization of additional chemokines beyond those included in our gene panel to systematically identify and study additional chemokine foci.

To further clarify this important point, we included the following text in the revised discussion on page 18:

“The mechanisms underlying selective enrichment of distinct myeloid cell populations within both *CXCL8* and *CXCL9/10/11* foci are incompletely understood and may involve combinatorial signaling of distinct chemokine receptors. For instance, relatively low expression of *CXCR2* on neutrophilic myeloid cells within *CXCL8* foci suggests that additional chemotactic signaling pathways, such as CSF3/CSF3R, may be required for the focal accumulation. Similarly, accumulation of myeloid cells within *CXCL9/10/11* foci despite expression of CXCR2 may indicate the involvement of other chemokine receptors, such as CSF1R, in this process. Furthermore, concurrent expression of ligands (*CXCL2*, *CXCL8* and *CSF3*) and cognate receptors (*CXCR2* and *CSF3R*) on neutrophilic cells within *CXCL8* foci could indicate autocrine signaling and self-recruitment potential.”

3. Neutrophils express both ligands (CXCL2, CXCL8, CSF3) and receptors (CXCR2, CSF3R). Discuss potential autocrine signaling and self-recruitment mechanisms.

Please see our response to comment 2.

4. Coculture experiments suggest neutrophils suppress T cell proliferation. Validate this function in spatial data by confirming reduced proliferation in T cells localized to CXCL8 foci.

We appreciate this suggestion. To address this comment, we quantified the proliferative proportion of T cells within different compartments of the parenchyma. Consistent with our experimental data, we observed a significant reduction in T cell proliferation within CXCL8 foci when compared to both CXCL9/10/11 foci or the remaining parenchyma. The results of this analysis were included as revised Extended Data Fig. 13c.

We included the following text in the revised results on page 14:

"Consistently, T cells within *CXCL8* foci displayed a significant reduction in proliferative proportion compared to other regions within the parenchyma (Extended Data Fig. 13c)."

5. HPV- tumor cells in both CXCL10 and CXCL8 foci share features like low HPV gene expression and reduced proliferation. Clarify how much these phenotypes arise from microenvironmental cues vs. intrinsic viral gene regulation loss.

We agree with the reviewer that intrinsic and extrinsic factors may be responsible for reduced HPV gene expression in a subset of HPV-OPSCC cells. The HPVoff phenotype, characterized by lack of HPV gene expression and reduced proliferation, was evident in both CXCL8 and CXCL9/10/11 foci. Our experiments suggest that different extrinsic factors may contribute to the HPVoff phenotype. While hypoxia may drive the HPVoff phenotype within CXCL8 foci, elevated IFN-gamma signaling is associated with HPVoff cells within CXCL9/10/11 foci. However, it is possible that HPVoff cells also arise in the absence of any external factors either stochastically or by epigenetic predisposition.

To address the reviewer's concern, we discussed this possibility in the revised discussion on page 19:

"Whether cell-intrinsic factors, such as stochastic HPV gene expression or epigenetic predisposition, can promote the HPVoff phenotype requires further study."

Moreover, the differentiation status and lineage relationship between HPV+ and HPV- cells, and whether these differ between foci types are unclear.

Point well taken. A better understanding of the lineage relationships between HPVon and HPVoff cells *in vivo* is crucial to further characterize the mechanisms underlying the

formation of HPVoff cells. However, such studies will likely depend on lineage tracing experiments in animal models, which we think are beyond the scope of this study.

To address the first part of the reviewer's comment, we considered epithelial cell types that were assigned to cancer cells based on the single-cell RNA-seq reference profiles. This analysis revealed clear differences between *CXCL8* and *CXCL9/10/11* HPVoff as well as HPVon cells, suggesting an association between differentiation status and HPV phenotype. These results are now included as revised Extended Data Fig. 14m.

Additionally, we included the following text in the revised results:

"Notably, HPVoff cells within *CXCL8* foci did not display characteristics of increased IFN-γ signaling, further supporting the idea that different mechanisms may give rise to HPVoff cells (Extended Data Fig. 14l), potentially further affected by distinct differentiation states (Extended Data Fig. 14m)."

6. While myeloid HLA-II expression is linked to T cell exhaustion (Fig. 3h), prior studies (e.g., PMID: 39105803) have reported HLA-II expression in HPV+ tumor cells.

We thank the reviewer for bringing the publication by Yan et al. to our attention. The study by Yan et al. compared HPV+ and HPV- OPSCCs and demonstrated that carcinoma cells derived from HPV+ OPSCCs are characterized by increased HLA-II and antigen presentation signature expression when compared to HPV- tumors.
First, we would like to emphasize that HPVoff cells, i.e. carcinoma cells from HPV+ OPSCC that lack detectable HPV transcripts, represent a distinct physiological entity within HPV+ OPSCC tumors that is different from carcinoma cells derived from HPV- tumors as shown by Puram et al (PMID: 37012457). Therefore, we would not expect the differences between HPVon and HPVoff cells from HPV+ OPSCC to reflect differences observed between HPV+ and HPV- OPSCC.

Include analysis on HPV+ tumor cell HLA-II expression and its contribution to T cell dysfunction.

To address this point, we considered HLA-II expression on HPVon cells and potential implications on T cell dysfunction as suggested by the reviewer. This analysis revealed an increased spatial association of HLA-II$^+$ HPVon cells with various T cell subsets when compared to HLA-II$^-$ HPVon cells, suggesting that HLA-II expression on HPVon cells may reflect increased exposure to IFN-gamma as well as contribute to T cell dysfunction within treatment-naïve HPV-OPSCC. This data is now included as revised Extended Data Fig. 10a.

The following text was included in the revised results on page 11:

"Consistently, HLA class II-expressing carcinoma cells displayed increased spatial association with different T cell subsets (Extended Data Fig. 10a)."

Additionally, HPV- tumor cells show stronger antigen presentation (Fig. 5g), which contradicts prior findings. Reassess and clarify result reliability.

Our analysis suggests that HPVoff cells within *CXCL9/10/11* foci display increased proximity to different T cell subsets, including IFN-gamma expressing T cells, and a concomitant upregulation of gene expression programs reflective of IFN-gamma response, such as antigen processing and presentation signatures. To address the reviewer's concern, we also performed a similar analysis considering HPVoff cells within *CXCL8* foci, which did not display characteristics of increased IFN-gamma levels. This analysis showed that, while *CXCL8* foci HPVoff cells displayed significantly lower expression of cell cycle genes, no significant differences were observed in gene signatures related to IFN-gamma signaling and antigen presentation as shown in revised Extended Data Fig. 14l. This suggests that elevated expression of the antigen presentation signature is restricted to HPVoff cells within *CXCL9/10/11* foci and is likely a result of IFN-gamma exposure.

We included the following text in the revised manuscript on page 16:

"Notably, HPVoff cells within *CXCL8* foci did not display characteristics of increased IFN-γ signaling, further supporting the idea that different mechanisms may give rise to HPVoff cells (Extended Data Fig. 14l), potentially further affected by distinct differentiation states (Extended Data Fig. 14m)."

Yan et al. further demonstrated that overexpression of HPV genes within head and neck cancer cell lines leads to upregulation of HLA-II in vitro. We also evaluated expression of HLA-II genes within HPVon and HPVoff carcinoma as well as non-malignant epithelial cells. Although no significant differences were detected, possibly due to the relatively small sample number, HPVon cells displayed a trend towards higher expression of HLA-II as shown in the revised Extended Data Fig. 14d, consistent with observations made by Yan et al.

The following text was included in the revised manuscript on page 15:

"HPVoff cells also displayed a tendency towards reduced HLA class II expression, consistent with previous observations[30] (Extended Data Fig. 14d)."

Lastly, we have now included a reference to Yan et al. in the revised manuscript.

7. Since CXCL10 and CXCL8 foci can co-exist in the same tumor, analyze inter-patient variability in foci proportion and whether this correlates with clinical outcomes. Consider using public datasets (e.g., PMIDs: 37012457, 38857913, 39105803) or TCGA-HNSCC to validate key findings and assess prognostic significance.

We thank the reviewer for this comment. As suggested by the reviewer, we analyzed the TCGA-HNSCC bulk RNA-seq data set and observed substantial variability in the expression of *CXCL2/8* and *CXCL9/10/11*. In addition, we did not observe a clear association between the expression of *CXCL8* and *CXCL10*, possibly indicating that *CXCL8* and *CXCL9/10/11* foci may form independently as shown in the revised Extended Data Fig. 13d-f. We also performed survival analysis considering expression-based stratification of the HPV-HNSCC samples. This analysis revealed significantly reduced survival of patients that are characterized by high expression of both CXCL2 and CXCL8. In contrast, CXCL9 displayed the opposite trend, but did not reach statistical significance. We included these new results as revised Figure 4i and Extended Data Fig. 13g.

The following text was included in the revised results on page 14:

"To further explore the clinical significance of distinct chemokine foci, we analyzed additional HPV-HNSCC samples from The Cancer Genome Atlas (TCGA). Correlation analysis of chemokine expression agreed well with expression patterns within our SGE data (Extended Data Fig. 13d,e). Furthermore, we did not observe a correlation between *CXCL10* and *CXCL8* expression within the HPV-HNSCC TCGA cohort potentially indicating independent formation of different chemokine foci classes (Extended Data Fig. 13f). Notably, chemokine expression - based survival analysis showed that elevated expression of both *CXCL8* and *CXCL2* associates with reduced overall survival in HPV-HNSCC (Fig. 4i, Extended Data Fig. 14g)."

The following text was included in the revised discussion on page 18:

"Analysis of TCGA data revealed that elevated intratumoral expression of both *CXCL2* and *CXCL8* was predictive of reduced overall survival. Consistently, higher serum CXCL8 levels have been associated with reduced overall survival in patients with HPV-associated cancers[41]. If focal *CXCL8* expression within the tumor parenchyma contributes to serum CXCL8 requires further evaluation."

8. Chemokine signaling analysis is currently limited to tumor and immune cells. Examine the role of stromal cells (e.g., fibroblasts, endothelial cells) in ligand/receptor expression and immune modulation.

This is a very good suggestion. However, considering the design of the 297 gene panel, which was tailored towards the characterization of different immune cell types and states, we don't think a meaningful analysis is possible in this regard. Of these 297 genes only five and seven were included as fibroblast and endothelial markers, respectively. We are hopeful that the latest Xenium versions, which support substantially greater numbers of probes, will enable a more comprehensive analysis of the various relevant cell types found within the TME.

9. Conclusions regarding the role of hypoxia and inflammatory signaling in shaping

immune foci (CXCL8, CXCL10) require validation in an independent cohort or external datasets.

This point is well taken. Unfortunately, there are no published Xenium HNSCC datasets. However, to address this comment, we obtained and analyzed publicly available HNSCC Visium data sets. The results of this independent analysis agreed well with our original analysis and further support our findings. More specifically, we observed that the hypoxia signature exhibits higher expression in parenchymal Visium spots with detectable *CXCL8* levels, whereas *CXCL9/10/11* marks spots that are characterized by elevated IFN-gamma signature expression. These findings were consistent across multiple samples obtained from independent studies. We included these supporting findings as revised Extended Data Fig. 12.

The following text was included in the revised Results section on page 13:

“In addition, analysis of multiple independent published HNSCC spatial transcriptomic data sets further supports the link between hypoxia and IFN-γ signaling in the formation of *CXCL8* and *CXCL9/10/11* foci, respectively (Extended Data Fig. 12a-c).”

10. Figure presentation and layout issue:
- The ordering and layout of Figures 1 and 5 disrupt logical flow. Reorganize figures for improved narrative coherence and readability.

The figures were reorganized as suggested in the revised manuscript.

- The workflow diagram (Fig. 1a) should include number of patients and clarify sample pairing.

Data availability for each patient was included in the revised Figure 1.

- Use standardized terminology consistently across text and figures (e.g., “malignant/non-malignant” vs. “HPV-OPSCC/normal”).

We thank the reviewer for pointing this out. We changed all names to non-malignant and HPV-OPSCC, respectively.

Reviewer #2 (Remarks to the Author)

I co-reviewed this manuscript with one of the reviewers who provided the listed reports. This is part of the Nature Communications initiative to facilitate training in peer review and to provide appropriate recognition for Early Career Researchers who co-review manuscripts.

Reviewer #3 (Remarks to the Author):

In this manuscript by Sievers and colleagues, they described the spatial transcriptomic landscape of HPV-OPSCC by integrating single cell RNA sequencing (5'-GEM) and 10X Xenium in-situ. In addition, the mechanisms underlying immune escape despite the presence of HPV antigens were examined in the spatial context. A cohort of 15 HPV-OPSCC samples were included in study. Seven HPV16-positive HPV-OPSCC samples and four adjacent non-malignant mucosa samples were profiled spatially for immune cell organization, cancer cell states, and cellular interactions.

While the single cell study is not considered novel, the data generated was primarily for cell type annotations especially the immune cells found in the tumours. Subsequently they used this information to design a custom panel of 297 genes for the spatial annotations and analysis. While HPVoff phenomenon has already been published, they identified distinct CXCL9/10/11 foci and CXCL8 foci which appear to be novel.

I have some comments and suggestions:

1) The majority of the data presented are descriptive and based on a very small number of samples using spatial gene expression data. While some of the observations are potentially interest, my main concern would be of false positives due to the small sample size and lack of statistical consideration for multiple testing. Findings of immune compartment differences between tumor and normal samples, for example in Fig 2e-h, should be validated in an independent cohort or confirmed in an orthogonal assay. Some of the cell types described (Tex, Treg) appear to be very low by proportion.

This point is well taken. We validated the immune-related differences that were observed while comparing non-malignant and HPV-OPSCC stroma using immunofluorescence (IF). Consistent with our Xenium data analysis, the IF data confirmed a clear enrichment of exhausted T cells within the stroma of HPV-OPSCC.

To address the reviewer’s comment, we included the new IF analysis as revised Extended Data Fig. 7 and included the following text edit in the revised manuscript on page 7/8:

“We observed a greater proportion of Tex and Tex_prol cells in HPV-OPSCC stroma compared to the mucosal stroma (Fig. 2e,f). Increased T cell exhaustion within the carcinoma-associated stroma was further supported by multiplex immunofluorescence, potentially reflecting enhanced T cell TCR engagement and activation (Extended Data Fig. 7).”

2) Fig 3h is too complicated to comprehend. They may want to move it to supplementary or replace it with simpler diagrams to highlight the main important observations.

We apologize for not making it clearer. To address the reviewer's concern, we expanded the revised figure legend of Fig. 3h to include the following explanation:

"The width of the alluvia/flows between the first and second axis or the third and fourth axis represent the number of cells of a given cell type that express a specific ligand or receptor, respectively. The alluvia between the second and the third axis correspond to the number of instances the indicated ligand/receptor-expressing cells localize in close proximity to satisfy the nearest neighbor relationship."

3) For data presented in figures 4 & 5, it is clear that samples NCI-8 and NCI-10, their HPV transcripts count were very low in CXCL9/10/11 foci in addition to CXCL8 foci. Therefore results presented were not consistent across the 7 malignant samples.

We appreciate the reviewer's observation and agree that HPV gene expression displays variability across samples. However, we would also like to point out that the expression values shown in original Figure 4a were row-standardized for each sample. The values therefore quantify HPV gene levels relative to the other chemokine compartments within the same sample. This was done to remove sample-specific expression effects, for instance resulting from patient-specific copy number alterations. Reduced relative levels of HPV genes in *CXCL9/10/11* foci could for instance result from higher relative expression in other parenchymal compartments. Furthermore, pseudo-bulk expression values, which were used in this analysis, can be susceptible to outliers, similar to mean expression values. Therefore, to study heterogeneity related to HPV gene expression, we instead considered the HPVoff rate, which is less affected by the problems outlined above. This analysis revealed that the HPVoff rate is significantly higher in *CXCL8* foci compared to both CXCL9/10/11 and remaining parenchyma as shown in the original Figure 5c. Notably, the HPVoff rates within *CXCL9/10/11* foci of NCI_8 and NCI_10 are comparable to those of other samples:

Sample_id	HPVoff rate within *CXCL9/10/11* foci
NCI_6	0.05760297
NCI_9	0.06517349
**NCI_8**	**0.08262295**
NCI_15	0.08563392
**NCI_10**	**0.18991017**
NCI_21	0.19984434
NCI_12	0.42285983

They did partially attempt to address this in figure 5 but due to the small number of malignant samples (n=7), they need to confirm their findings on CXCL9/10/11 foci and CXCL8 foci in an independent cohort ideally using Xenium.
If not, minimum requirement would be to perform immunofluorescence to determine whether:

We agree that it would be ideal to validate our findings in other HPV-OPSCC Xenium data sets. Unfortunately, we are not aware of any publicly available HPV-OPSCC Xenium data sets. We also agree with the reviewer that immunofluorescence would be a powerful alternative strategy. However, visualizing HPV proteins in immunohistology assays, such as immunohistochemistry or immunofluorescence, is very challenging as there are no validated antibodies commercially available. For instance, we have not been able to validate specificity of commercially available HPV16 E6 and E7 antibodies. We agree that our critical finding should be validated in orthogonal assays. To validate our findings, we considered both scRNA-seq of this study and publicly available Visium data from multiple independent studies as explained in more detail below. Of note, the HPV status of the samples used to generate the published Visium data was either unclear or negative. Furthermore, the spot-based Visium does not allow to resolve single-cell expression profiles. Concerns related to HPVoff rates were therefore addressed using our scRNA-seq data.

- CXCL8 foci are depleted of HPV expression, enriched for HIF proteins or other hypoxia markers and depleted of proliferation markers (e.g.MKI-67).

To address the reviewer's comment, we analyzed our scRNA-seq data and were able to validate that epithelial cells positive for *CXCL8* expression are characterized by increased HPVoff rates, express increased levels of hypoxia markers and display transcriptional signs of reduced proliferation.

These new results were included as revised Extended Data Figures 11c-e and 14e.

We also included the following text in the revised manuscript on page 15:

"The enrichment of HPVoff cells within *CXCL8* foci is further supported by our scRNA-seq analysis (Extended Data Fig. 13e)."

and on page 12 of the revised manuscript:

"In agreement with the single-cell SGE data, analysis of HPV-OPSCC scRNA-seq data revealed increased IFN-γ signature expression within *CXCL9/10/11*-positive epithelial cells, while *CXCL8* expression was associated with reduced cell cycle scores and elevated hypoxia signature levels (Extended Data Fig. 11b-e)."

These associations were further supported by the analysis of publicly available Visium data, which showed elevated hypoxia signature expression within multiple samples from independent studies. These results were included as revised Extended Data Fig. 12a-c. Please also see our response to reviewer #1 comment 9.

- CXCL9/10/11 foci are enriched for markers for IFN gamma as well as to differentiate HPVon and HPVoff regions as some of their current samples (NCI-8 and NCI-10) showed significant reduction in HPV protein expression.

To address this valid concern, we first analyzed our scRNA-seq data. This analysis confirmed a significantly higher expression of IFN-gamma signature within *CXCL9/10/11*-positive epithelial cells as shown in the revised Extended Data Fig. 11b.

As in our previous response, the following text was included in the revised manuscript on page 12:

"In agreement with the single-cell SGE data, analysis of HPV-OPSCC scRNA-seq data revealed increased IFN-γ signature expression within *CXCL9/10/11*-positive epithelial cells, while *CXCL8* expression was associated with reduced cell cycle scores and elevated hypoxia signature levels (Extended Data Fig. 11b-e)."

We further distinguished HPVon and HPVoff *CXCL9/10/11*-positive epithelial cells, as suggested by the reviewer, and observed a consistent trend towards elevated IFN-gamma signature expression within HPVoff cells ($p = 0.064$).

The following text was included in the revised manuscript on page 16:

"A trend towards increased IFN-γ signaling signature expression within HPVoff cells was also seen in scRNA-seq data (Extended Data Fig. 14k)."

Elevated expression of the IFN-gamma signature was further observed within *CXCL9/10/11*-positive Visium spots.

These results were included as revised Extended Data Fig. 11a-c. Please also see our response to reviewer #1 comment 9.

- If they are able to show that these distinct foci are statistically associated with the phenomenon described, I would be in favour of this manuscript getting published in Nat Comm.

We thank the reviewer for the supportive comment.

4) In the in vitro study on the effect of hypoxia on T-cell mediated tumor cell killing, what was the corresponding effect of hypoxia on the E7-specific T cells?

We apologize for not making this clearer in the manuscript. It is well established that hypoxia reduces T cell-mediated cytotoxicity by impairing T cell function. However, we would like to clarify that the T cells used for the in vitro studies were not exposed to hypoxia. In these experiments, only HNSCC cells were exposed to hypoxia prior to co-culture, while the T cells were kept under normoxic conditions.

To further clarify the reviewer's comment, we included the following text in the revised figure legends of Fig. 5f and Extended Data Fig. 14h:

“Of note, the T cells were not exposed to hypoxia and were maintained in normoxic conditions throughout the experiments.”

We thank the reviewer for bringing this to our attention.

MINOR comments
1) Figure 4H: define which group the yellow and white boxes refer to

We updated the revised Figure 4h to include an explanation.

Reviewer #4 (Remarks to the Author):

I co-reviewed this manuscript with one of the reviewers who provided the listed reports. This is part of the Nature Communications initiative to facilitate training in peer review and to provide appropriate recognition for Early Career Researchers who co-review manuscripts.

Reviewer #5 (Remarks to the Author):

This study by Sievers et al, integrates single-cell RNA sequencing (scRNA-seq) and single-cell spatial gene expression (SGE) profiling to investigate how cellular states and immune organization contribute to HPV-associated oropharyngeal squamous cell carcinoma (HPV-OPSCC). Applying a specific 297-gene panel (derived from scRNAseq) onto biopsies from seven HPV16-positive tumour samples and four non-malignant mucosal regions, using the Xenium spatial platform, the authors explore various neighbour hood analyses: gross (stroma vs parenchyma), chemokin-derived and traits-based neighbourhoods.They show that spatially distinct chemokine-defined immune “foci” (CXCL9/10/11 vs CXCL8) exist and appear to influence immune and tumor cell composition. They also identify HPV transcript-negative (“HPVoff”) senescent-like cancer cells that may evade HPV-specific immunity. The team conducted a numner of mechanistic experiments that further implicate hypoxia and IFN-γ in shaping these tumor states.
The experiments conducted in this study are interesting, relevant and provide some interesting insights into the spatial organisation of HPV-positive tumors. The data shown here also add a valuable resource for re-analyses (both the scRNAseq and spatial analyses). There are a few issues that need to be addressed as listed below, and listed according to the sequence they appear in the manuscript, and I have used the Figure numbers to refer to these.

1. Figure 1 shows the scRNAseq data and derivation of the panel used for spatial analyses. The author use the latter to divide tumors in to ‘parenchymal’ and ‘stromal’ regions, and how HPV gene expression varies between these, including showing proliferation markers. There are few things to note here:
a. I wonder if the classification of ‘parenchymal’ and ‘stromal’ is the right terminology here..it sounds like tumor core, and tumor adjacent may be more appropriate.

‘Advancing front’ is also a useful term although these are not resections, so I am not sure if that is appropriate as these are biased samples as they are biopsies and may not represent the true tumor-stroma interface.

We thank the reviewer for this comment. These biopsies were obtained under direct visualization of the tumor mass itself. Tumors are comprised of both regions of interconnected carcinoma cells and intervening stroma within what is generally called the tumor. We agree that since these biopsies were not obtained from the tumor-adjacent normal tissue margin of a surgical resection that it would not be appropriate to refer to them as the ‘invasive front’ and for this reason in our opinion it would not be correct to refer to the stroma as ‘tumor adjacent’. Considering how these samples were obtained and consistent with established literature, we think that tumor parenchyma (referring to the regions of interconnected carcinoma cells) and stroma is the correct terminology to use in the context of the tumor microenvironment.

b. Reliance only on keratin-based classification may oversimplify intermediate states that may be present in the tumor such as cells undergoing EMT, especially at the stromal–epithelial interface, and the biological significance of proliferating stromal cells remains to be clarified.

We agree with the reviewer that keratin-based analysis may only allow for a broad classification of epithelial and non-epithelial cells, while a more resolved analysis of epithelial cell states was limited by the number of genes available for single-cell spatial gene expression (SGE) profiling (297). The genes included in the Xenium panel were selected with a particular focus on different immune cells and states. Although we agree with the reviewer that physiological processes such as EMT are of great importance, we don’t think this data set would allow for a meaningful analysis in this regard.

It is correct that we observed an increased proportion of proliferating stromal cells within the stroma of HPV-OPSCC compared to the non-malignant epithelium.

Interestingly, we also observed an enrichment of different proliferative T cell subsets within the HPV-OPSCC stroma, suggesting that the enrichment of proliferating cells may, in part, result from increased activation of distinct immune cell populations.

To address the reviewer’s comment, we added the following text to the revised manuscript on page 8:

“The enrichment of proliferative T cell subsets may in part explain the increased proportion of proliferating cells within the HPV-OPSCC stroma (Fig. 1k).”

2. Figure 2 maps scRNA-seq-defined cell states onto single-cell spatial gene expression for cell type annotation. They take the T/NK cells from stromal and parenchymal compartments and cluster and perform subtype annotations.

We thank the reviewer for this observation. It is correct that we performed the subset analysis of stromal and parenchymal T/NK and myeloid cells separately. The analysis was performed separately to overcome technical limitations of the Xenium technology. When we initially performed the analysis on stromal and parenchymal cells combined, we noticed that the parenchymal immune cells were characterized by relatively high expression of epithelial markers, such as keratins, while this was not the case for stromal immune cells. This led to clustering of cells based on the origin, i.e. stroma vs parenchyma and less by immune cell subset, complicating the analysis. It is a known problem that assignment of transcripts to individual cells, which depends on algorithmic image segmentation of the original FFPE section micrograph, can be challenging when multiple cells overlap. 10X Genomics is actively working on different solutions to overcome these problems. To mitigate this issue, we restricted the analysis to nuclear transcripts which are less affected by this problem. However, despite only considering nuclear transcripts, we still observed differences between stromal and parenchymal immune cells that likely resulted from misassigned transcripts (e.g. keratins).

Howere, here the T cells from stromal and parenchymal compartments were clustered and annotated separately prior to proportion comparisons. This biases subsequent analyses and introduces artificial differences that arise because they were analysed separately to begin with. Moreover, annotation consistency across compartments can be difficult to guarantee, direct comparisons of proportions may be biased by context-specific clustering, and cross-compartment relationships between T cell states may be obscured. A combined clustering and annotation strategy followed by stratified comparisons could have provided a more standardized framework for assessing proportional differences.

The immune cell sub-clustering was performed to further refine the initial cell-type calls. Separate analysis of stromal and parenchymal cells allowed us to exclude technical noise from the analysis and resolve distinct immune cell subsets more accurately and consistently, as we concluded from the consistent expression of marker genes within both stromal and parenchymal cells. To avoid any kind of bias that could result from separate classification of immune cell subsets, we did not compare proportions of individual immune subsets across compartments, i.e. the comparison of proportions/frequencies was always performed within the same compartment considering cells that were jointly analyzed. As an example, Figure 2f shows the comparison of stromal T/NK subset proportions between HPV-OPSCC and non-malignant samples that were jointly analyzed during T/NK subset classification. Similarly, Figure 3f focuses on immune cell subsets that were characterized within the parenchyma. The comparison between stroma and parenchyma (Figure 2g) does not consider T-cell subsets, but rather all cells classified as T cells based on scRNA-seq expression profiles and further quantifies the expression of indicated marker genes within all T cells.

To address the reviewer’s comment, in the revised manuscript we moved the following text from the methods to the discussion on page 21 for enhanced visibility:

“Despite restricting our single-cell SGE analysis to nuclear transcripts, SGE quantification may be affected by misassigned transcripts. Development of more accurate cellular segmentation methods to allow quantification of transcripts across entire cells is warranted.”

3. Figure 3 explores the chemokine landscape within HPV-OPSCC parenchyma, highlighting the emergence of spatially distinct chemokine “foci.” The authors show that T- and myeloid-cell attracting chemokines (CXCL9/10/11 and CXCL8) are not uniformly expressed, but instead form focal clusters with variable magnitude across tumours.

a. I fail to understand how they spefically zoomed in on this chemokine niche over other markers or cell types

In the present study we decided to focus on both myeloid and lymphoid cells within the context of HPV-OPSCC, which can both play important roles in the context of carcinogenesis. Furthermore, infiltration of immune cells into the tumor parenchyma is thought to be impaired in a variety of cancers leading to a dysfunctional immune phenotype generally referred to as ‘immune excluded’. To better understand parenchymal immune cell organization and the mechanisms underlying infiltration of different immune cells into the parenchyma we decided to focus on CXCL8 and CXCL9/10/11, which play well established roles in recruiting myeloid and T cells, respectively.

b. The authors also do not explain details on how they derive their neighbourhood and how they correct for multiple hypothesis testing.

We thank the reviewer for bringing this to our attention and allowing us to further clarify. The neighborhood was based on spatial proximity. The neighborhood of a specific cell is defined by it’s k-nearest neighboring cells, i.e. the k cells with the smallest Euclidean distance. A k-nearest neighbor analysis was used for both the classification of the stroma and parenchyma as well as the identification of the chemokine foci.

To address the reviewer’s comment, we included the following text in the revised methods section on page 27:

“Nearest neighbors were identified using Seurat::FindNeighbors considering Euclidean distances.”

All p-values associated with differential gene expression resulting from the comparison of groups of single cells were adjusted for multiple testing.

To clarify this, the following text was included in the revised methods on page 28:

“Differential gene expression analysis was performed using Seurat::FindAllMarkers considering adjusted p-values.”

We further clarified in the revised legends of Figures 4e-g:

“P-values; Wilcoxon rank-sum test; adjusted for multiple testing.”

c. The assignment of cells to foci is based on local transcript enrichment, which may be sensitive to resolution limits of the platform.

Yes, we agree with this point. Future Xenium advancements, that enable for a substantially greater number of probes, may further improve resolution of these chemokine foci.

d. The functional consequences of focal versus uniform chemokine expression are inferred but not directly tested, and whether these spatial chemokine niches actively direct cell migration or simply reflect regions of immune cell residence is unclear.

We agree with the reviewer that various aspects of the functional consequences of chemokine foci still require further characterization. Our interpretation of the data is that these chemokine foci may in fact both direct migration of distinct immune cells as well as reflect regions of immune cell residence. Recruitment of immune cells through chemokines/chemokine receptors is well established. For instance, it is widely accepted that *CXCL9/10/11* direct migration of $CXCR3^+$ T cells. Consistently, we find an enrichment of different T-cell subsets within *CXCL9/10/11* foci. However, *CXCL9/10/11* are known IFN-gamma response genes. Since IFN-gamma is produced by T cells, *CXCL9/10/11* foci could reflect increased local levels of IFN-gamma-producing T cells. Hence, local enrichment of T cells could explain focal expression of *CXCL9/10/11* which in turn leads to the recruitment of additional T cells in a positive feedback loop.

However, we also recognize that the process of chemokine foci maintenance and immune cell recruitment could be more complex and involve the interplay of multiple chemokines/chemokine receptors as it seems to be the case for *CXCL8* foci. In this regard, please see our response to reviewer #1 comment 2.

e. Moreover, the dominance of tumour cells as major chemokine producers complicates interpretation, as it raises the possibility that foci formation may represent tumour-intrinsic programs rather than solely immune-driven processes.

We agree with the reviewer’s assessment that chemokine foci are not solely immune-driven and can also result from the physical conditions within the tumor microenvironment as exemplified by focal *CXCL8* expression. Our analysis suggests that hypoxia represents a dominant factor driving the expression of *CXCL8* within carcinoma and potentially myeloid cells. However, the mechanisms underlying the formation of different classes of chemokine foci can differ as our analysis demonstrates. A comprehensive analysis of the mechanisms underlying the focal expression of various chemokines, for instance using advanced spatial transcription technologies, would be of great interest. We thank the reviewer for this remark.

f. Do these chemokine niches correlate with cell type specific niches?

Our enrichment analysis (Figure 3f) suggests that different classes of chemokine foci are characterized by the enrichment of distinct immune cell populations. This enrichment may be the result from the interplay of various signaling pathways.

Are you showing a difference between a neutrophil rich versus lymphocyte rich neighbourhoods, where the chemokines are the effect and not the cause?

Unfortunately, we are not able to infer cause and consequence within our data as these are clinical samples and we have no ability to perturb the system. Although we think this may be beyond the scope of this study, it will be of great interest to dissect these mechanisms in the future.

g. Ligand-receptor analyses should be cautioned as being quite speculative

Although in our analysis we have only considered ligand-receptor pairs for which a ligand-receptor relationship has been well established in the literature, we agree that our data is limited to establishing spatial proximity of expressed ligand-receptor pairs and does not demonstrate functional signaling. Future studies based on improved single-cell spatial transcriptome technologies may employ downstream gene expression signatures to further demonstrate productive receptor ligation.

To address the reviewer's comment, we included the following text in the revised discussion on page 19:

"Mechanistic studies designed to further assess productive proximal and distal signaling events affecting individual immune cells are warranted."

4. Figure 4 demonstrates that tumour-intrinsic gene programs, demonstrating that cancer cells within CXCL9/10/11 foci express IFN-γ–response and antigen presentation genes, whereas CXCL8-foci cells exhibit hypoxia and glycolysis signatures, reduced HPV and proliferation gene expression, and increased inflammatory cytokines that recruit neutrophils. The in vitro experiments support this notion quite nicely, although it is important to caution that we don't quite understand which is the 'chicken' and which is the 'egg' (if it even matters!). Nevertheless, these results underscore how tumour cells actively shape spatially segregated immunological niches through conserved, environment-dependent transcriptional programs.

We thank the reviewer for these supportive comments. We also agree that our data does not provide information related to the initiation of the chemokine foci. For instance, while maintenance of *CXCL9/10/11* foci may depend on T cell – derived IFN-gamma, the initial recruitment of T cells may depend on other factors, such as type I interferons, which can also drive expression of *CXCL9/10/11*.

To address the reviewer's comment, we included the following text in the revised discussion on page 19:

"Similarly, events underlying the initiation of chemokine foci formation require further analysis."

5. Figure 5 identifies HPVoff cells as a senescent-like tumour subpopulation that is enriched within CXCL8 foci and influenced by microenvironmental cues (maybe this is the 'chicken!). However, while this data highlights hypoxia- and IFN-γ–driven emergence of the HPVoff phenotype, these observations are largely correlative, and additional microenvironmental factors may contribute. The functional consequences of HPVoff cells, including immune evasion and SASP-mediated modulation, require further in vivo validation, and the p53 dependence of IFN-γ–induced senescence may limit generalizability across tumours with variable p53 status or with other HPV types

This point is well taken. The HPVoff phenotype may be induced by different factors. In the present study we focused on hypoxia and IFN-gamma signaling, which emerged from our Xenium data analysis and were further validated *in vitro.* While we agree that it is of great importance to further study the functional consequences of HPVoff cells, for instance with regard to their ability to further modulate the tumor microenvironment, such mechanistic HPVoff-focused studies likely require extensive animal experiments and we think are beyond the scope of this work.

We would also like to point out that majority of HPV-OPSCC is characterized by wild-type p53 status regardless of HPV type. Hence, if these observations can be further related to functional p53, the implications of the HPVoff phenotype described in this study are likely to be shared across the majority of patients with HPV-associated oropharyngeal carcinoma. Of note, all tumor samples in this study harbored wild-type *TP53*.

Minor issues:
1. Issued with batch correction and probe bias in Xenium need to be better discussed and laid out

Batch correction of both scRNA-seq and SGE data was performed prior to data integration using Harmony as outlined in the methods.

We also included updated text in the revised discussion to include the possibility of probe bias and off-target effects:

"Probe-based quantification of gene expression in SGE data may be affected by characteristics of transcripts and probes and, therefore, contain bias, such as over- or underestimation of actual transcript counts and off-target binding."

We would also like to point out that the majority of gene expression quantification performed in this study resulted in relative/comparative expression values resulting from the comparisons of different cell subsets, which should be similarly affected by any technical issues related to the probes. Hence, comparative gene expression quantification should reduce the effects of any probe biases.

We also included the estimated number of false positive transcripts per cell for each sample, which could indicate technical problems related to the probes, such as off-target binding, in the revised methods section on page 27:

"The estimated number of false positive transcripts per cell for each sample, computed based on negative control probes/codewords by the manufacturer's companion analysis software, is as follows: NCI_3: 0.101; NCI_4: 0.159; NCI_14: 0.0316; NCI_22: 0.0412; NCI_6: 0.0878; NCI_8: 0.0492; NCI_9: 0.0559; NCI_10: 0.0488; NCI_12: 0.0781; NCI_15: 0.0672; NCI_21: 0.0862."

2. Please confirm all the statistical analyses (Wilcoxon vs t-test) as some may be inconsistently applied across figures; authors should also clarify multiple testing correction.

We clarified how multiple testing was corrected and specified which statistical tests were performed in the corresponding figure legends.

3. Expanding these to other HPV types- do you expect these to be different? Perhaps a discussion on this

The following sentence was included in the revised manuscript on page 20:

"Studies that evaluate whether similar mechanisms exist in the context of other high-risk HPV types, benign low-risk HPV-associated tumors[43] or other virus-associated malignancies are warranted[44]."

4. Finally, a Discussion could on therapeutic implications, e.g., senolytic therapies, hypoxia-targeting, or LAG-3 blockade.

We thank the reviewer for this suggestion and included the following text in the revised discussion on page 19:

"Whether reduction of hypoxia levels, for instance through cytoreductive therapy, or LAG3 blockade could result in clinical benefit in HPV-OPSCC requires further investigation."

Senolytic therapies had already been discussed in the context of HPVoff cells on page 20 of the manuscript.

Reviewer #6

Cem Siever et al. demonstrated that spatial cellular organization underpins localized mechanisms of immune resistance in oropharyngeal cancer, utilizing integrated spatial and single-cell transcriptomic analyses. The study revealed that spatial positioning defines discrete immune foci, each characterized by unique immune and stromal cell compositions, and associated with locally restricted immunosuppressive mechanisms. These findings underscore the complex cellular interactions that establish immunosuppressive niches in HPV-associated oropharyngeal squamous cell carcinoma (HPV-OPSCC). However, several key concerns should be addressed before the manuscript can be considered for acceptance

Major Concerns:

1. The content of Figure 3E is unclear. It is not specified whether each point represents a cell cluster, a sample, or another biological or computational unit. Furthermore, the method by which these values were calculated is not adequately described. A detailed explanation should be included in the Methods section to clarify the computational approach and biological meaning of the plot should be mentioned in the figure legend.

We apologize for not describing the analysis more clearly.

We updated the revised legend of Fig. 3e to read:

"Scatter plot showing the ratio of the observed and expected proportion of cells classified as both CXCL9/10/11 and CXCL8 foci and the corresponding p-values for each HPV-OPSCC sample"

We also added the following text to the revised methods on page 28/29:

"Mutual exclusivity between *CXCL8* and *CXCL9/10/11* foci was evaluated considering the number of parenchymal cells assigned to *CXCL8* foci only, *CXCL9/10/11* foci only, *CXCL8* and *CXCL9/10/11* foci and neither *CXCL8* nor *CXCL9/10/11* foci. Assuming independence the probability of a cell belonging to both *CXCL8* and *CXCL9/10/11* foci was obtained as the product of the two marginal probabilities to reflect expected events and compared to the observed probability estimated based on the observed event counts. The resulting probabilities were compared to obtain the logodds = log(p_observed / p_expected). Corresponding p-values were obtained considering the complete event-count contingency table and a Fisher's exact test."

2. To support the conclusion that HPV infection induces CXCL8/9/10/11 gene expression specifically on tumor cells on HPV-positive patients, the authors should include comparative analyses using scRNA-seq and validate the findings with immunohistochemical staining in HPV-negative tumor samples. Additionally, the spatial and cellular specificity of CXCL8/9/10/11 expression should be clarified

using both single-cell and spatial transcriptomic analyses.

We apologize for not stating our conclusions more clearly. It is not a conclusion of our study that HPV infection induces CXCL8/9/10/11 gene expression. Based on our Xenium data analysis and in vitro validation, we concluded that two major drivers of *CXCL8* and *CXCL9/10/11* foci development are hypoxia and IFN-gamma signaling within the tumor parenchyma. We did not mean to suggest that chemokine foci only form in HPV-associated malignancies. In fact, accumulations of immune cells that bear similarities to the chemokine foci we describe in this study have been reported in both colorectal and lung cancer as we discuss in the manuscript.

However, we do agree with the reviewer that validation of our key findings would strengthen our conclusions.

As an orthogonal data set, we considered both our scRNA-seq data and published Visium data from HNSCC. Of note, we were able to validate our key finds in multiple independent HNSCC data sets, including HPV-negative HNSCC as shown in revised Extended Data Fig. 11c.

For additional information regarding the validation, we would like to draw the reviewer's attention to our responses to reviewer #1, comment 9 and reviewer #3, comment 3.

3. The interaction analysis presented (e.g., in Figures 3H and S9A) should also be supported by single-cell-based cell–cell communication inference (e.g., CellPhoneDB, NicheNet, or similar tools), covering all major ligand-receptor interactions and associated signaling pathways. Likewise, the genes shown in Figure 3G related to cell–cell communication require validation and elaboration through single-cell analysis.

This is an excellent idea. We performed ligand-receptor interaction analysis using the R package CellChat. This analysis largely confirmed the interactions we identified within our spatial data. Of note, some interactions were not detected by CellChat likely because expression was low in the scRNA-seq data (e.g. CXCR2). However, this analysis is consistent with our spatial analysis and also suggests robust interactions between LAG3 expressed on T cells and MHC class 2 on both myeloid and T cells.

We included the results of this analysis as revised Extended Data Fig. 10b.

The following text was added to the revised manuscript on page 11:

"Further supporting these findings, similar ligand-receptor interactions were also identified within scRNA-seq data (Extended Data Fig. 10b)."

We thank the reviewer for this suggestion.

4. In the section "Cancer cells within CXCL9/10/11 and CXCL8 foci express

conserved gene programs," the features highlighted in Figure 4A should be further validated using single-cell transcriptomic data. This would help to confirm the presence and conservation of the identified gene programs at single-cell resolution, and clarify their association with specific tumor subpopulations.

This point is well taken. To address the reviewer's comment, we analyzed our scRNA-seq data and were able to validate that epithelial cells characterized by the expression of *CXCL8* express increased levels of hypoxia markers and display transcriptional signs of reduced of proliferation. Similarly, we observed significantly higher expression of IFN-gamma signature within *CXCL9/10/11*-positive epithelial cells. The results obtained from the scRNA-seq data analysis are consistent with our spatial analysis and were included were included as revised Extended Data Figures 11b-e.

We also included the following text in the revised manuscript on page 12:

"In agreement with the single-cell SGE data, analysis of HPV-OPSCC scRNA-seq data revealed increased IFN-γ signature expression within *CXCL9/10/11*-positive epithelial cells, while *CXCL8* expression was associated with reduced cell cycle scores and elevated hypoxia signature levels (Extended Data Fig. 11b-e)."

These associations were further supported by the analysis of publicly available Visium data, which showed elevated hypoxia signature expression within CXCL8-positive spots within multiple samples across independent studies. Simiarly, elevated expression of the IFN-gamma signature was further observed within *CXCL9/10/11*-positive Visium spots. These new results were included as revised Extended Data Fig. 12.

Please also see our responses to reviewer #1 comment 9 and reviewer #3 comment 3.

5. The authors demonstrate that HPV gene expression is suppressed under hypoxic conditions and upon IFN-γ stimulation. However, the underlying mechanisms remain unclear and require further investigation with additional supporting evidence.

We fully agree with the reviewer that a detailed analysis of the mechanisms underlying regulation of HPV gene expression is of great importance. However, elucidating such mechanisms will likely require extensive and comprehensive experimentation using various experimental systems and assays. Considering the extent of such mechanistic studies, which may take multiple years to complete, we think that this is beyond the scope of this study.

Minor Concerns:
1. In the Methods section, the criterion "3) 250 > genes with non-zero UMI counts > 5000" is ambiguous and unclear. It is recommended to revise this phrasing for clarity—for example, "cells with < 250 and >5000 detected genes (genes with non-zero UMI counts)"
—to ensure unambiguous interpretation.

We clarified the statement based on the reviewer's recommendation.

2. The discussion regarding the limitations of spatial gene expression analysis is currently placed at the end of the Methods section. This content should be moved to the Discussion section where it would be more appropriately contextualized.

The entire paragraph on limitations was moved to the revised discussion.

3. The resolution of several figures is too low to discern fine details, especially when zooming in. Higher-resolution images should be provided for clarity and to meet publication standards.

We apologize for low-quality figures that were included to comply with file size restrictions. We will make all original PDFs available to ensure highest resolution of the figures.

4. The figure legend for Figure 3D does not match the labels or content displayed in the figure. The legend should be revised to accurately reflect the figure panels and the data they represent.

We reviewed Figure 3d and the corresponding legend. Unfortunately, we were unable to detect any apparent mismatches. We found a mismatch between the legends of Figures 2e and f, which we corrected.

5. Statistical comparisons should be included in Figure 4H, as well as in other figures throughout the manuscript wherever applicable. This is necessary to support the validity of observed differences and conclusions drawn from the data.

Statistical comparisons have been added to Fig. 4H, as well as to Fig. 5k, as recommended.

Second round of revision of manuscript entitled:

Spatial cellular order underlies locally-confined mechanisms of immune resistance in oropharyngeal cancer (NCOMMS-25-38811-T)

REVIEWER COMMENTS

Reviewer #1 (Remarks to the Author):

The authors have provided a revision with additional data and improved writing, yet several concerns remain to be addressed.

1. Clarification of epithelial cell clusters: The explanation regarding normal versus tumor epithelial cells remains unclear. Normal epithelial cells in non-malignant tissues should differ from tumor epithelial cells in HPV-OPSCC based on HPV expression and transcriptional profiles (e.g., Fig. S5), yet Figure 2A shows similar epithelial cluster distributions in both tissue types. The authors should specify which of the 10 epithelial clusters represent normal versus tumor cells and clarify the differences in their distributions between non-malignant epithelium and OPSCC tissues.

We apologize for the lack of clarity and agree with the reviewer that non-malignant and malignant epithelium, e.g. HPV-OPSCC, display profound differences. However, while malignant transformation involves acquisition of various abnormal features that are absent in normal epithelial cells, malignant carcinoma cells still retain an epithelial character as exemplified by the continued expression of various epithelial lineage markers. To exemplify this notion reviewer figure 1 shows an unrelated HPV-OPSCC tissue sample (not included in this study) that contains both malignant and non-malignant epithelium. As this example shows, while HPV gene expression is mostly restricted to the malignant cells, the epithelial marker KRT15 is uniformly expressed across both malignant and non-malignant epithelial cells and largely absent in the subepithelial stromal compartments.

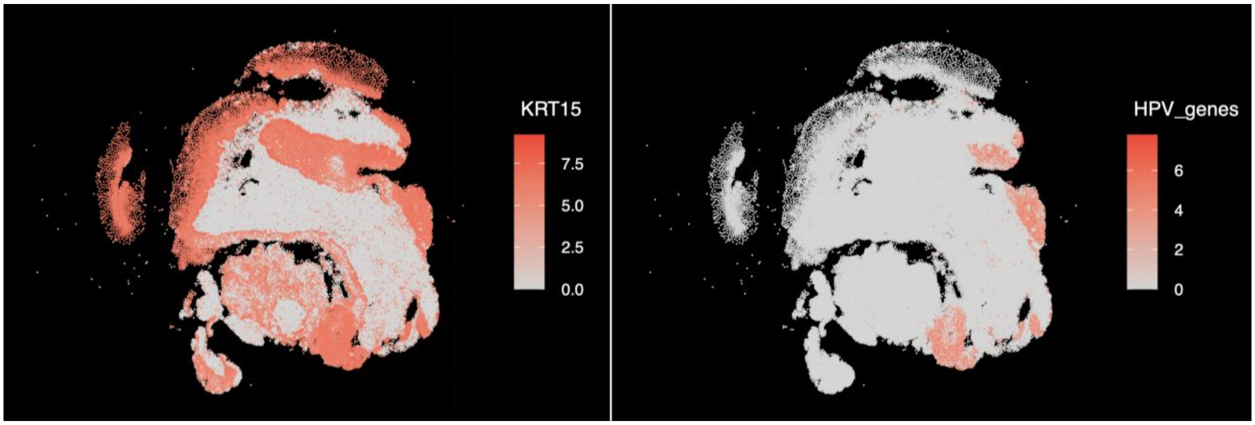

Reviewer figure 1. Retention of epithelial marker gene expression in carcinoma cells. Scatter plots showing an unrelated HPV-OPSCC sample containing both non-malignant and malignant epithelial cells colored by KRT15 (left) or HPV gene (right) expression. Malignant epithelial cells are characterized by KRT15 and HPV gene expression, while non-malignant epithelial cells lack HPV gene expression.

Hence, due to the retained epithelial character of carcinoma cells, i.e. expression of epithelial marker genes, our scRNA-seq derived cell-type expression profiles enabled us to identify epithelial cells regardless of malignancy state. We also agree with the reviewer that scRNA-seq epithelial clusters may contain both malignant and non-malignant cells. Based on our spatial analysis we concluded that HPV gene expression is highly indicative of malignancy.

To address the reviewer's comment, we quantified HPV gene expression within the scRNA-seq epithelial clusters and observed profound variability potentially indicating varying degrees of malignant cells. As the reviewer correctly suspected, considering the composition of the singleR classification results within our spatial data revealed a clear separation between malignant and non-malignant epithelial tissues, suggesting that some scRNA-seq epithelial profiles more strongly associate with non-malignant states while others reflect a greater degree of malignancy. Consistently, we observed that HPV gene expression tended to be greater in scRNA-seq clusters that associated with malignant spatial gene expression samples.

These results are now included as revised Extended Data Figure 5b.

In addition, the following text was included in the revised manuscript (page 6): "HPV-OPSCC and non-malignant epithelium displayed clear differences related to epithelial cell-type classification possibly explained by variable proportions of malignant and non-malignant epithelial cells within individual scRNA-seq clusters as suggested by different HPV gene expression levels (Extended Data Fig. 5b)."

We thank the reviewer for this comment.

2. HPV+ vs. HPV- Tumor Cell Comparisons: While HPV+ and HPV- OPSCC may represent different diseases, comparisons between HPVon and HPVoff tumor cells (both from HPV+ OPSCC) are still necessary. Previous studies have shown that such distinctions have functional significance, as in EBV+ nasopharyngeal carcinoma (see PMID: 33531485). The authors should conduct a similar analysis and discuss how viral antigen presentation relates to tumor immune responses.

We appreciate this suggestion and agree with the reviewer viral gene expression within tumor cells may have functional consequences. The publication shared by the reviewer demonstrates clear expression differences between EBV-positive and EBV-negative nasopharyngeal carcinoma cells. Similarly, Puram et al (Nature Genetics, 2023), who first described HPVoff cells in HPV-OPSCC have comprehensively characterized these cells in their original publication and describe profound transcriptional variability associated with the HPVoff phenotype. For instance, HPVoff cells are characterized by reduced expression of cell cycle genes and increased expression of an epithelial senescence signature. Furthermore, the work of Puram et al. also includes a comparison of HPVon/HPVoff cells from HPV-OPSCC with HPV-negative HNSCC. In contrast, the focus of our study is on the spatial aspects of HPVon/HPVoff cells within HPV-OPSCC. Our analysis suggests that HPVoff cells can be induced by different external factors such as IFN-gamma exposure or hypoxia. Considering these observations and adding to the initial characterization of HPVoff cells by Puram et al, we experimentally demonstrate in this study that hypoxia treatment of HPV-positive HNSCC cell lines leads to reduced HPV gene expression and reduced susceptibility towards HPV-specific T cell - mediated killing.

As a kind reminder the relevant text in this regard is provided below: “Although substantial tumor cell killing was observed under normoxic conditions, hypoxic conditioning of tumor cells prior to co-culture with T cells led to a significant reduction in tumor cell killing (Figure 5e-f, Extended Data Fig. 14g-i). These results suggest that hypoxia may directly promote evasion of HPV-specific T cell immunity, possibly through the combination of hypoxia-induced reduction in HPV gene expression and reduced tumor cell HLA class I.”

In the discussion we had included: “Furthermore, prolonged exposure of HPV-OPSCC cell lines to hypoxia led to reduced susceptibility to HPV-specific T cell-mediated cytotoxicity, consistent with a significant reduction in HPV gene and MHC class I and II expression. Therefore, it is possible that the HPVoff phenotype provides protection from anti-tumor immunity specifically directed against HPV-associated antigens, reminiscent of an adaptive immune resistance mechanism[42].”

To address the reviewers comment we performed differential gene expression analysis comparing HPVon and HPVoff cells for both CXCL8 and CXCL9/10/11 foci separately. The results

are consistent with our previous analysis and show an enrichment of IFN-gamma related terms, such as 'Antigen Presentation: Folding, assembly and peptide loading of class I MHC' or 'Interferon gamma signaling', within genes differentially upregulated with CXCL9/10/11-HPVoff cells. Of note, enrichment of IFN-gamma related terms was substantially less pronounced in CXCL8-HPVoff cells. The results of this analysis were included as revised Extended Data Figure 14k.

We agree with the reviewer that a detailed understanding of how enhanced antigen processing and presentation and reduced expression of HPV genes/antigens affects immunogenicity of HPVoff cells is of great importance.

Therefore, we included the following text in the revised discussion (page 20): "How long HPVoff cells retain presentation of immunogenic HPV antigens following termination of HPV gene transcription *in vivo* requires additional analysis."

We also thank the reviewer for bringing the publication to our attention, which we referenced in our revised manuscript.

3. The newly added single-gene prognostic and correlation analyses are promising, but module score-based analyses for CXCL2/8 and CXCL9/10/11 should be performed to further strengthen the findings.

To address this comment, we performed the survival analysis using stratification based on the combined expression of CXCL2/8 or CXCL9/10/11. Interestingly, the combined expression of CXCL2/8 shows a significant association with survival.

These results were included in the revised Extended Data Figure 13g. Accordingly, the text in the results section (page 15) was revised to read: "Notably, chemokine expression - based survival analysis showed that elevated expression of both *CXCL8* and *CXCL2*, alone or in combination, associates with reduced overall survival in HPV-HNSCC (Fig. 4i, Extended Data Fig. 13g)."

We thank the reviewer for this suggestion.

4. The authors should integrate their explanation regarding technical limitations directly into the manuscript.

We appreciate this comment. However, it is our understanding that Nature Communications generally prefers discussion of the limitations within the Discussion section. We had therefore included one paragraph discussing potential technical limitations of the technology within the Discussion section.

As a kind reminder, the following paragraph was included in the Discussion section: “Limitations to spatial gene expression analysis exist. Low transcript counts in SGE can also complicate cell type and state analysis. Probe-based quantification of gene expression in SGE data may be affected by characteristics of transcripts and probes and, therefore, contain bias, such as over- or underestimation of actual transcript counts and off-target binding. Despite restricting our single-cell SGE analysis to nuclear transcripts, SGE quantification may be affected by misassigned transcripts. Development of more accurate cellular segmentation methods to allow quantification of transcripts across entire cells is warranted.”

Since we had initially included this paragraph in the Discussion section under the impression that this complies with the requirements/preferences of the journal, we would like to defer this decision to the editorial team.

5. Mechanism underlying neutrophil aggregation in CXCL8-foci remains insufficiently explained. Even considering the CSF3-CSF3R axis, macrophages express CSF3R at levels comparable to or higher than Moma_Neu, yet are less prevalent within CXCL8-foci. This discrepancy should be further clarified.

This point is well taken. We agree with the reviewer that our data, due to inherent limitations, does not fully explain accumulation of neutrophils within CXCL8-foci. Based on our analysis (Figure 3g), highest expression of CSF3R at the transcript level is observed in CXCL8-foci Neu cells, while CXCL9/10/11-foci MoMa cells also express relatively high levels. Since the spatial gene expression data sets only measures transcript levels of a select number of genes (297 out of about 25,000), it is possible that additional relevant chemokine receptors are not represented in our gene panel. It is also possible that interactions between chemokine receptors determine migration behavior of myeloid cells. For instance, it is possible that higher CSF1R expression levels on MoMa cells retain these cells within CXCL9/10/11 foci, while dominant CSF3R expression (combined with reduced CSF1R expression) drives neutrophilic cells towards CXCL8 foci. In addition, cell surface protein levels of chemokine receptors may be subject to post-translational regulation. Hence, it is possible that detectable expression at the transcript level does not accurately reflect chemokine receptor levels on the cell surface. A better understanding of the underlying mechanisms will likely depend on different experimental technologies involving measurements at the protein level and/or surveying expression of a greater number of genes. Alternatively, such mechanisms could be studied using animal models. For instance, to determine the relevant receptors, cell-type specific knock-out could be performed while observing migratory behavior of different myeloid populations. However, this would first require identification of a model that recapitulates these finding made in human HPV-OPSCC. Although we fully agree with the reviewer that these are crucial questions, which think such experiments are beyond the scope this study.

As a kind reminder, to highlight these limitations we had already included the following text in the Discussion of the manuscript: “The mechanisms underlying selective enrichment of distinct myeloid cell populations within both *CXCL8* and *CXCL9/10/11* foci are incompletely understood

and may involve combinatorial signaling of distinct chemokine receptors. For instance, relatively low expression of *CXCR2* on neutrophilic myeloid cells within *CXCL8* foci suggests that additional chemotactic signaling pathways, such as CSF3/CSF3R, may be required for the focal accumulation. Similarly, accumulation of myeloid cells within *CXCL9/10/11* foci despite expression of *CXCR2* may indicate the involvement of other chemokine receptors, such as CSF1R, in this process. Furthermore, concurrent expression of ligands (*CXCL2*, *CXCL8* and *CSF3*) and cognate receptors (*CXCR2* and *CSF3R*) on neutrophilic cells within *CXCL8* foci could indicate autocrine signaling and self-recruitment potential."

To address the reviewer's concern, we expanded our discussion of these inherent limitations to include the following text in the revised discussion (page 18-19): "In addition, chemokine receptor transcript counts may not accurately reflect effective cell-surface receptor protein levels due to post-translational regulation. Further study of the mechanisms underlying selective enrichment of distinct myeloid populations within different tumor-parenchymal regions, for instance using protein-level analysis, is warranted."

6. While lineage-tracing experiments in animal models are challenging, the authors could explore the differentiation relationship between HPV-on and HPV-off cells using pseudotime differentiation analysis with single-cell or spatial transcriptomic data.

We agree with the reviewer that transitions between HPVon and HPVoff cells are of great interest. A finding of our study is that different external factors can promote the HPVoff phenotype. More specifically, we find that both IFN-gamma signaling and hypoxia enhance the HPVoff phenotype. Pseudotime analysis has generally been developed to study continuous developmental processes. Considering that the HPVoff phenotype does not represent a distinct stable developmental state, but rather a rapidly inducible physiological state that displays characteristics of reversibility, we don't know how a meaningful trajectory analysis can be performed. For instance, a pseudotime analysis would require that HPVon and HPVoff cells form distinct clusters. Unfortunately, in our analysis the HPVoff cells do not stand out as a developmentally distinct and distinguishable population that would allow us to perform this kind of analysis.

7. HPV-Positive Cells and T-Cell Associations: HPV-positive cells show increased spatial associations with nearly all T-cell subpopulations, including cytotoxic T cells (with higher association than some Tex subclusters). This observation is broad and does not clarify the relationship with T-cell dysfunction. More specific analyses using single-cell or spatial transcriptomic data to investigate interactions or direct correlations with Tex exhaustion features would be more informative.

First, we would like to apologize for this misunderstanding and clarify that our analysis does not show that HPV-positive cells associate with various T-cell subpopulations. Similarly, we also do not find that cytotoxic T cells are spatially associated with HPV-positive (HPVon) cells to a greater degree than exhausted T cells.

Our analysis shows that there is increased association between HPVoff cells, not HPVon/HPV-positive cells, and different immune cell subsets, including exhausted T-cell subsets.

The dysfunctional or exhaustion T cell phenotype, characterized by co-expression of exhaustion and effector marker genes, within the tumor microenvironment is generally considered to be associated with anti-tumor immunity. For instance, in a previous study we have experimentally demonstrated that exhausted T cells are enriched for anti-tumor specificities directed against tumor neoantigens (Sievers et al. Cancer Cell 2023). Similarly, Oliveira et al. have more comprehensively demonstrated that both tumor-specific $CD4^+$ and $CD8^+$ T cells predominantly display an exhausted phenotype (Nature 2021, PMID: 34290406, Nature 2022, PMID: 35508657). In contrast, T cells specific for tumor-unrelated antigens predominantly exhibited a memory T cell phenotype also characterized by relatively high expression of TCF-1. Consistently, we find that the exhausted T-cell phenotype is significantly enriched within the tumor parenchyma (please see Figure 2c,g). As the exhausted T cell phenotype can result from chronic TCR stimulation, the parenchymal enrichment could reflect increased antigen availability and TCR signaling within the tumor parenchyma. Other factors that contribute to T-cell exhaustion are, for instance, hypoxia or acidosis.

Considering the various HPV-OPSCC and immune cell subsets, our spatial neighborhood analysis shows that HPVoff cells within CXCL9/10/11 foci display increased co-localization with the following lymphocyte subsets: IFN-gamma-high Tex_infg, Tex_int and NK cells (please see Figure 5h). Although similar tendencies were present, we did not observe statistically significant co-localization with other lymphocyte populations such as Tregs, Tem, Tex or Tcd4_n.cm, possibly due to our limited sample size (n = 7 HPV-OPSCC samples). These finding are consistent with an IFN-gamma mediated reduction of HPV gene expression.

However, the reviewer raises a very important point. An additional interpretation of this data could be that HPVoff cells actively contribute to T-cell exhaustion, for instance, through the production of immunosuppressive cytokines (please see Figure 5m).

To highlight this possibility, we included the following text in the revised discussion: “Whether HPVoff cells can promote T-cell exhaustion, for instance through production of immunosuppressive cytokines, requires further analysis.”

We thank the reviewer for this comment, for the thorough review of our manuscript and the other excellent remarks.

Reviewer #2 (Remarks to the Author):

I co-reviewed this manuscript with one of the reviewers who provided the listed reports. This is part of the Nature Communications initiative to facilitate training in peer review and to provide appropriate recognition for Early Career Researchers who co-review manuscripts.

Reviewer #3 (Remarks to the Author):

The authors have provided an adequate rebuttal. I have no further comments.

We would like to thank the reviewer for the positive response.

In addition, since Reviewer #5 was unavailable to complete the review this time, I was asked by the handling editor, Dr. Lizhao Wu, to comment on the concerns that Reviewer #5 raised, and to determine whether in my opinion the authors have adequately addressed Reviewer #5's concerns in the revised manuscript. Here is my response:

***

I have read through the comments of Reviewer #5 and the authors' replies. All the replies are well taken, and the comments have been adequately addressed. Several comments concerning internal validation and caution of overinterpreting the data also overlap with those from other reviewers.

These study limitations have been addressed in the Discussion section. Overall, I support publication of this manuscript.

***

We are grateful to the anonymous reviewer for the additional help/efforts with evaluating our responses to reviewer #5 and appreciate the positive feedback.

Reviewer #4 (Remarks to the Author):

I co-reviewed this manuscript with one of the reviewers who provided the listed reports. This is part of the Nature Communications initiative to facilitate training in peer review and to provide appropriate recognition for Early Career Researchers who co-review manuscripts.

Thank you!

Cem Siever et al. demonstrated that spatial cellular organization underpins localized mechanisms of immune resistance in oropharyngeal cancer, utilizing integrated spatial and single-cell transcriptomic analyses. The study revealed that spatial positioning defines discrete immune foci, each characterized by unique immune and stromal cell compositions, and associated with locally restricted immunosuppressive mechanisms. These findings underscore the complex cellular interactions that establish immunosuppressive niches in HPV-associated oropharyngeal squamous cell carcinoma (HPV-OPSCC). However, several key concerns should be addressed before the manuscript can be considered for acceptance

Major Concerns:

1. The content of Figure 3E is unclear. It is not specified whether each point represents a cell cluster, a sample, or another biological or computational unit. Furthermore, the method by which these values were calculated is not adequately described. A detailed explanation should be included in the Methods section to clarify the computational approach and biological meaning of the plot should be mentioned in the figure legend.
2. To support the conclusion that HPV infection induces CXCL8/9/10/11 gene expression specifically on tumor cells on HPV-positive patients, the authors should include comparative analyses using scRNA-seq and validate the findings with immunohistochemical staining in HPV-negative tumor samples. Additionally, the spatial and cellular specificity of CXCL8/9/10/11 expression should be clarified using both single-cell and spatial transcriptomic analyses.
3. The interaction analysis presented (e.g., in Figures 3H and S9A) should also be supported by single-cell-based cell–cell communication inference (e.g., CellPhoneDB, NicheNet, or similar tools), covering all major ligand-receptor interactions and associated signaling pathways. Likewise, the genes shown in Figure 3G related to cell–cell communication require validation and elaboration through single-cell analysis.
4. In the section “Cancer cells within CXCL9/10/11 and CXCL8 foci express conserved gene programs,” the features highlighted in Figure 4A should be further validated using single-cell transcriptomic data. This would help to confirm the presence and conservation of the identified gene programs at single-cell resolution, and clarify their association with specific tumor subpopulations.

5. The authors demonstrate that HPV gene expression is suppressed under hypoxic conditions and upon IFN-γ stimulation. However, the underlying mechanisms remain unclear and require further investigation with additional supporting evidence.

Minor Concerns:

1. In the Methods section, the criterion “3) 250 > genes with non-zero UMI counts > 5000” is ambiguous and unclear. It is recommended to revise this phrasing for clarity—for example, “cells with < 250 and >5000 detected genes (genes with non-zero UMI counts)”—to ensure unambiguous interpretation.
2. The discussion regarding the limitations of spatial gene expression analysis is currently placed at the end of the Methods section. This content should be moved to the Discussion section where it would be more appropriately contextualized.
3. The resolution of several figures is too low to discern fine details, especially when zooming in. Higher-resolution images should be provided for clarity and to meet publication standards.
4. The figure legend for Figure 3D does not match the labels or content displayed in the figure. The legend should be revised to accurately reflect the figure panels and the data they represent.
5. Statistical comparisons should be included in Figure 4H, as well as in other figures throughout the manuscript wherever applicable. This is necessary to support the validity of observed differences and conclusions drawn from the data.
